# Synthesis of Dithienylcycloalkene Molecular Switches Enabled by a Bench‐Stable Ti‐Complex

**DOI:** 10.1002/chem.202502730

**Published:** 2025-10-17

**Authors:** Marcell M. Bogner, Márton Hegedűs, Péter P. Kalapos, Attila Kunfi, Gábor London

**Affiliations:** ^1^ Institute of Organic Chemistry HUN‐REN Research Centre for Natural Sciences Budapest 1117 Hungary

**Keywords:** cyclobutenes, dithienylethenes, fatigue resistance, McMurry coupling, photoswitch

## Abstract

Herein, we report a set of cycloalkene‐bridged dithienylethene (DTE) molecular switches with ring sizes ranging from 4‐ to 7‐membered rings. These analogs were accessed via McMurry carbonyl coupling using a bench‐stable Ti(IV)‐complex reagent instead of the generally applied fuming, liquid TiCl_4_, making the synthesis more practical and reproducible. The efficiency of the reaction was dependent on the ring size, providing the lowest yield for the largest, 7‐membered ring. Structures having 3‐membered cyclopropene bridges were prepared using an alternative strategy. Remarkably, the comparison of the photoswitching properties of the series showed that molecules bearing a strained cyclobutene bridge outperform the other switches in terms of reversibility and fatigue resistance.

## Graphical abstract

Herein we report a set of cycloalkene‐bridged DTEs with ring sizes ranging from 4‐ to 7‐membered rings. The synthesis of these analogs was performed via a key McMurry coupling step, which was enabled by a practical, bench‐stable Ti(IV)‐complex. Remarkably, it is shown that the strained cyclobutene bridge outperforms other rings, even the most commonly featured cyclopentene, in terms of reversibility and fatigue resistance.

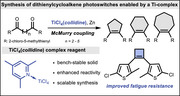

## 1 Introduction

**Figure 1:**
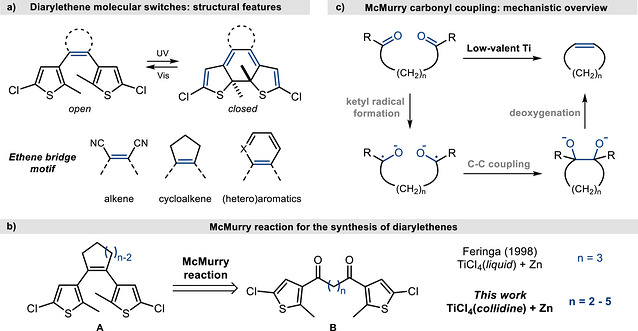
a) Diarylethene‐type molecular switches with different generations of ethene bridge motifs. b) Key disconnection in the synthesis of cycloalkene‐bridged dithienylethenes; previously reported and improved McMurry reaction conditions. c) Mechanistic overview of the intramolecular McMurry carbonyl coupling reaction.

Diarylethenes (DAE) are a widely studied and highly optimized class of molecular switches^[^ ^1^, ^2^, ^3^ ^]^ (Figure 1a) with multidisciplinary applications ranging material science,^[^ ^4^, ^5^, ^6^, ^7^ ^]^ chemical biology,^[^ ^8^, ^9^, ^10^, ^11^ ^]^ and catalysis.^[^ ^12^, ^13^, ^14^ ^]^ Structural modifications for tuning DAE photoswitches are possible through either editing the ethene bridge or the side aryl groups.^[^ ^1^, ^15^, ^16^ ^]^ Both approaches have been taken previously to describe a variety of different DAEs all aiming for a set of essential properties such as thermal stability, reversibility, and fatigue resistance. Historically, switches having noncyclic ethene bridges^[^ ^17^, ^18^, ^19^, ^20^ ^]^ (alkene, Figure 1a) quickly evolved to structures with cycloalkene motifs of different kinds, including cyclopentene‐derivatives^[^ ^21^, ^22^ ^]^ (cycloalkene, Figure 1a), (hetero)aromatic^[^ ^23^, ^24^, ^25^, ^26^, ^27^ ^]^ and numerous other functionally designed systems.^[^ ^15^, ^16^ ^]^ As a result, DAEs are now available as a robust and versatile platform for photochromic applications. Despite the continuous interest in novel diarylethene scaffolds cyclopentene‐type DAEs remain the most widely used structures, while other members of the perhydro‐cycloalkene bridged dithienylethenes (DTEs) have been mostly neglected.^[^ ^28^ ^]^

**Scheme 1:**
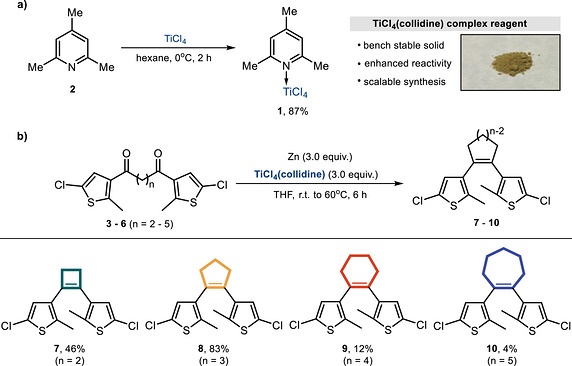
a) Single‐step synthesis of TiCl_4_(*collidine*) complex for the McMurry carbonyl coupling. b) Syntheses of cycloalkene‐type dithienylethenes **7**–**10**.

Synthetic access to DAEs of type **A** having a cyclopentene‐bridge (Figure 1b, n = 3, compound **8**) was first described by Feringa and coworkers^[^ ^29^ ^]^ in 1998 via a key carbonyl coupling step under McMurry conditions. Notably, at the same time, Fan and coworkers published a similar synthesis of closely related structures.^[^ ^30^ ^]^ Compound **8** features fast and reversible photoswitching, alongside with good synthetic accessibility. The synthetic route of Feringa and coworkers proved to be so robust and efficient that although countless DAE photoswitches have been produced since, state‐of‐the‐art synthetic campaigns still feature the McMurry reaction to form the pentacyclic ethene bridge. This key disconnection of the cycloalkenes simplifies the tricyclic ring systems to diaryl‐diketones of type **B**, which are easily accessible in a few steps via conventional transformations. Intramolecular McMurry carbonyl coupling leverages in situ generated low‐valent titanium species for ketyl radical formation, which is followed by C─C coupling resulting in a pinacol intermediate, and subsequent deoxygenation affords the olefinic product^[^ ^31^, ^32^ ^]^ (Figure 1c). Widely adopted conditions usually feature TiCl_4_ or TiCl_3_ and an internal reductant^[^ ^31^, ^32^ ^]^ (mostly Zn, or other metals/metal hydrides such as Li, Mg or LiAlH_4_). Although these reagent systems have proved to be highly efficient in carbonyl couplings, the use of extremely reactive titanium reagents raises practicality issues. In this communication we aimed to provide an alternative source of Ti(IV), a stable complex, offering easy reaction setup without loss of efficiency. TiCl_4_(*collidine*) (**1**, Scheme 1a), a bench‐stable Ti(IV)‐complex was first used in organic synthesis by Suga, Ukaji, and coworkers in 2018 for a conjugate addition reaction of benzyl radicals to electron‐deficient olefins.^[^ ^33^ ^]^ Collidine (2,4,6‐trimethylpyridine, **2**) was expected to serve as a multipurpose additive: it forms a bench‐stable, solid complex with TiCl_4_, acts as a base in the McMurry reaction (neutralizing acidic species) and also stabilizes low‐valent titanium species (Ti^0^, Ti^II^) via coordination, based on previous screening of additives.^[^ ^34^ ^]^ Despite its potential, it has never been used in carbonyl couplings before and ultimately remained hidden from wide‐range adoption.

## 2 Results and Discussion

First, we set out to test the TiCl_4_(*collidine*) complex (**1**) as a reagent in the McMurry reaction for the synthesis of DTE photoswitches with cycloalkene bridges. As a result, we disclose the syntheses and photochromic properties of a series of perhydro‐cycloalkene bridged DTEs and explore the effect of the bridge‐ring size on their photoswitching efficiency. Finally, we highlight the increased fatigue resistance in DTEs bearing a strained cyclobutene‐bridge.

Our synthetic approach to perhydro‐cycloalkene derived DAEs were based on the previous reports of Feringa^[^ ^35^ ^]^ leveraging a key McMurry cyclization step to form the desired photoswitches (Scheme 1b). This route involves diketone intermediates **3**–**6**, which were easily accessible from the corresponding dicarboxylic acids in 2 steps (see section S2, Supporting Information). Our initial experiments for the preparation of **8** were carried out in the presence of TiCl_4_ and Zn yielding the desired product in 41–70% yield, depending on the scale of the reaction (Table 1, Entry 1). Due to the high reactivity of liquid TiCl_4_ small scale preparations were challenging and resulted in decreased isolated yields (Table S1, Supporting Information). To overcome the apparent difficulties of this procedure, we prepared the TiCl_4_(*collidine*) complex (**1**) in a single step using 2,4,5‐trimethylpyridine (**2**) and TiCl_4_ (Scheme 1a). This bench‐stable, yellow solid Ti(IV)‐complex is easily accessible in large scales applying the procedure described by Gui and coworkers via precipitating the complex from hexanes.^[^ ^36^ ^]^ By using this complex reagent to generate the low‐valent titanium active species in situ with Zn as a reducing agent **8** was isolated in good yield, comparable to the best yields obtained with the original TiCl_4_/Zn system (Table 1). Notably, under identical conditions, intermolecular carbonyl couplings did not yield the desired targets (for further details see section S10.4, Supporting Information). We explored other conditions using different Ti sources and reducing agents (Table 1), however, they all failed to give the desired cyclized product.

**Table 1:** Optimization of McMurry reaction conditions.

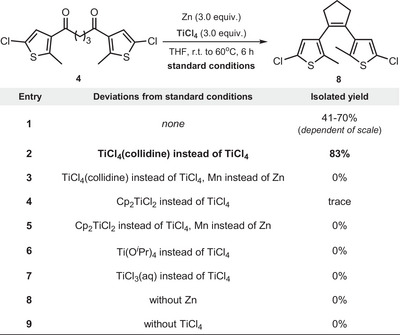

The identified reaction conditions enabled the robust and scalable (up to > 1 g) synthesis of cyclopentene‐bridged DTE **8**, demonstrating the reactivity and practicality of TiCl_4_(*collidine*) in carbonyl coupling. Furthermore, cyclobutene, cyclohexene, and cycloheptene (**7**, **9**, and **10**, respectively), analogs (Scheme 1b) were prepared to showcase the generality of this method. All the DTE photoswitches were accessed from the corresponding diketones (**3**, **5**, and **6**) in differing yields with respect to the newly formed ring size. Despite the more strained 4‐membered cyclobutene ring, **7** was isolated in a significantly higher yield than the ring‐expanded cyclohexene (**9**) or cycloheptene (**10**) derivatives. These are in line with previous observations on intramolecular McMurry reactions.^[^ ^31^ ^]^ Each analogue was synthesized bearing a chlorine on the thiophene ring, which enabled flexible late‐stage functionalization via umpolung reactivity or cross‐coupling strategies. Notably, DTEs with 4‐ and 7‐membered cycloalkene bridges have not been described so far.

**Figure 2:**
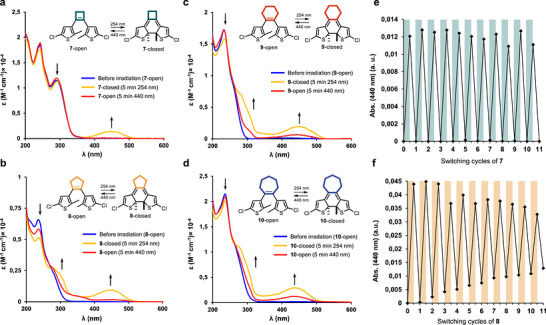
UV–vis spectroscopic characterization of dithienylcycloalkenes (MeCN, c = 3 × 10^−5^ M, rt) a) **7**, b) **8**, c) **9**, and d) **10**. e) Absorbance of **7** monitored at 440 nm upon alternating irradiation with UV (254 nm, 60 s) and visible (440 nm, 60 s) light sources. f) Absorbance of **8** monitored at 440 nm upon alternating irradiation with UV (254 nm, 60 s) and visible (440 nm, 60 s) light sources.

The isolation of compounds **7**–**10** allowed the assessment of the effect of bridge‐ring size on photoswitching properties. Notably, systematic comparison of the ring‐size effect has been done only for 5‐ and 6‐membered ring systems.^[^ ^37^ ^]^ The photochemical properties of each product were investigated by UV–vis spectroscopy under identical conditions. UV‐vis absorption spectra were recorded in a range of solvents with different polarities, including acetonitrile, dichloromethane and methanol (see section S3, Supporting Information). As expected, due to the lack of functional groups and the generally hydrophobic structures no significant solvent effect was observed. Similarly, the expansion of bridge‐ring size did not alter the spectra significantly compared to cyclopentene‐based DTE **8** that exhibited a high‐intensity band at 240 nm (Figure 2b–d). Notably, an additional band appeared at 300 nm in case of **7** (Figure 2a), featuring the strained tetracarbon bridge. Subsequently, photoswitching properties were explored by UV–vis measurements following irradiation at different wavelengths. Characteristic changes in the spectra that are indicative of conrotatory electrocyclic isomerization of DTEs were detected in all cases (Figure 2a–d) upon irradiation with UV light (*λ*_max_ = 254 nm). Appearance of a low‐intensity band in the visible region (400–500 nm) indicated the formation of the conjugated π‐system of the closed isomers with smaller HOMO‐LUMO gaps. Cycloreversion could be induced by irradiation with visible light (*λ*_max_ = 440 nm), however, differences were found in reversibility of photoswitching and thus fatigue resistance for the examined derivatives. In case of cyclopentene‐bridged **8**, even after extended visible‐light irradiation, the original spectrum could not be recovered completely (see section S2, Supporting Information). The remaining absorption band that was observed at 300–400 nm and at 400–500 nm range indicated the irreversible formation of a side‐product, which is likely a dihydrodithiaacenaphthylene derivative^[^ ^38^, ^39^ ^]^ that is a major source of fatigue in this process. Although slightly shifted, these irreversible bands were also apparent in the spectra of **9** and **10** after the first switching cycle. DTE **7**, however, made an exception with complete reversibility of the absorption band in the visible region. This prompted us to explore fatigue resistance more carefully. By assessing absorption intensities at 440 nm for **7** and **8** upon alternating UV and visible light irradiation, the fatigue resistance of the mentioned derivatives was investigated and compared. After 11 cycles of alternating UV (60 s) and visible (60 s) light irradiation, the difference between the two systems was clearly apparent (Figure 2e, 2f for **7** and **8**, respectively).

While the photoswitching efficiency of **8** decreased rapidly over the course of the experiment, the reversibility of **7** remained comparable to the original state, aside from small deviations in the absorbance of the closed form, due to low change of intensities. These results suggest that the incorporation of a strained cycloalkene bridge provides increased resistance against fatigue caused by byproduct formation in DTEs. This property can be attributed to the band separation in the UV–vis spectrum. Upon irradiation of **7** with 254 nm UV light, the photoisomerization could be selectively induced, however, when prolonged irradiation was applied with a 312 nm light source, the loss of reversibility was similar to the other analogs (see section S4, Supporting Information).

**Scheme 2:**
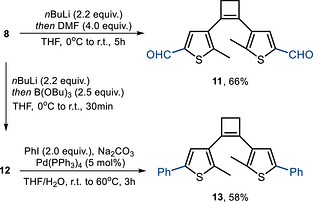
Syntheses of cyclobutene‐bridged DTE derivatives.

To showcase the versatility of the new cyclobutene‐bridged photoswitch two further analogs were built on this scaffold (Scheme 2). Dichloro derivative **7** was subjected to lithiation by *n*‐butyllithium to enable umpolung reactivity with different electrophiles such as DMF or tributyl borate.^[^ ^35^ ^]^ Accordingly, dialdehyde **11** and bis(dibutyl‐boronate) **12** were prepared. This latter compound was converted (without isolation) into the diphenylated derivative **13** via a subsequent Suzuki coupling with iodobenzene. Both compounds showed reversible photoswitching upon irradiation in a range of different solvents (see section S2, Supporting Information). The substituents introduced also affected the spectral properties of the resulting photoswitches. Absorption bands in the visible region significantly redshifted in the case of dialdehyde **11** compared to **7**, with a local maximum absorption peak at 590 nm. ^1^H NMR measurements showed the reversible isomerization of compound **11** with maximum open/closed ratio of 1:0.15 and 1:0.25 upon UV‐irradiation of the open form in C_6_D_6_ and CD_3_CN, respectively (see section S6, Supporting Information). Thermal ring opening was not observed after 60 minutes at rt (see section S5, Supporting Information).

**Figure 3:**
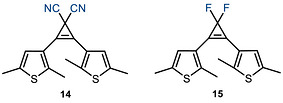
Prepared cyclopropene‐bridged dithienylethenes.

In addition, we explored the syntheses of cyclopropene‐bridged DTEs as well. Unfortunately, the general synthetic sequence (shown in Scheme 1b) did not afford the corresponding diketone from malonyl chloride, so we took a different approach. Via a formal C1 insertion strategy^[^ ^40^, ^41^ ^]^ we successfully prepared the highly strained cyclopropene derivatives **14** and **15** (Figure 3), featuring electron‐withdrawing substituents on the ethene bridge (for further details, see section S8, Supporting Information).

Based on our previous results regarding enhanced fatigue resistance of more strained cyclobutene‐bridged DTEs, we anticipated **14** and **15** to be highly reversible photoswitches. Additionally, it has been explored in the literature^[^ ^42^ ^]^ how electron‐withdrawing substituents on the ethene‐bridge further improve photoswitching efficiency. On the contrary, both molecules (**14** and **15**) exhibited an irreversible photoreaction upon prolonged irradiation with UV light (for further details see section S8, Supporting Information), likely resulting in decomposition.

## 3 Conclusion

In summary, we have successfully introduced TiCl_4_(*collidine*) as a bench‐stable reagent, and a safer alternative of liquid TiCl_4_, for intramolecular McMurry carbonyl coupling to access DTEs with cycloalkene bridges. This simple and practical method enabled the synthesis and characterization of a series of derivatives with different bridge ring sizes ranging from 4‐ to 7‐membered rings, and the uncovering of the improved fatigue resistance of strained cyclobutene‐based DTEs. These results complement previous studies by Hecht and coworkers on fatigue resistance^[^ ^42^ ^]^ by bringing bridge‐ring size in molecular design.

## Supporting Information

The authors have cited additional references within the Supporting Information.^[^ ^43^, ^44^, ^45^ ^]^

## Conflict of Interest

The authors declare no conflict of interest.

## Supporting information



Supporting Information

## Data Availability

The data that support the findings of this study are available in the supplementary material of this article.
