# Supplementary material for: Synthesis of Dithienylcycloalkene Molecular Switches Enabled by a Bench‐Stable Ti‐Complex
Source: Chemistry. 2025 Oct 17;32(29):e02730. doi: 10.1002/chem.202502730 (PMC13451566; doi:10.1002/chem.202502730)

Supporting Information

Synthesis of dithienylcycloalkene molecular switches enabled by a bench-stable Ti-complex

Marcell M. Bogner[1], Márton Hegedűs[1], Péter P. Kalapos[1], Attila Kunfi[1] and Gábor London[1]*

*Corresponding Author Email: london.gabor@ttk.hu

[1]Institute of Organic Chemistry, HUN-REN Research Centre for Natural Sciences, 1117 Budapest, Magyar tudósok krt. 2., Hungary

Table of Content

S1 Materials and methods S2
S2 Experimental Protocols and Product Characterization S3
S2.11 Product characterization S9
S3 UV-Vis analyses S15
S4 Switching cycles – Analysis of fatigue resistance S25
S5 Kinetic studies on the thermal ring opening reaction S30
S6 ^{1}H NMR spectroscopic characterization of the photochemical transformation of compound 11 .... S31
S7 Photoswitching in solid phase S34
S8 Cyclopropene-bridged dithienylethenes S35
S8.1 Experimental Protocols and Product Characterization S36
S8.2 UV-Vis analyses S38
S9 LED emission spectra S41
S10 Background S42
S10.1 Previous uses of $TiCl_4$(collidine) S42
S10.2 Photochemical side-reactivity of DTEs S42
S10.3 Improving fatigue resistance of DTEs S43
S10.4 Limitations and alternatives S44
S11 References S46
S12 NMR spectra S47

S1 Materials and methods

Commercial reagents, solvents, and catalysts (Sigma-Aldrich, Fluorochem, and VWR) of reagent grade were purchased and used without further purification. Solvents for extraction or column chromatography were of a technical quality. Organic solutions were concentrated by rotary evaporation at 40°C, unless otherwise noted. Thin-layer chromatography was carried out on “Merck silica gel 60 F_{254}” or “Merck aluminum oxide 60 F_{254} neutral” type UV-active silica or alumina sheets. Column chromatography was performed using a Teledyne Isco CombiFlash Rf+ automated flash chromatographer with “RediSep R_f GOLD” silica gel or basic alumina column at 25(±1)°C. The cartridge was filled with Zeochem “ZEOprep 60 25–40 µm” silica gel or EcoChrom “MP Alumina B - Super I” basic alumina. Analytical RP-HPLC-UV/vis-MS measurements were carried out using a Shimadzu LCMS 2020 instrument applying a Gemini C18 column (100 mm × 2.00 mm I.D.) in which the stationary phase was 5 µm silica with a pore size of 110 Å. The chromatograms were recorded with a UV–vis diode array (190–800 nm) and an ESI-MS detector. The following linear gradient elution profile was applied for the LC–MS measurements: 0% → 100% B in 6.5 min then 100% → 0% B in 0.5 min, then 0% B for 1 min with eluents A (2% HCOOH, 5% CH_3CN and 93% water) and B (2% HCOOH, 80% CH_3CN and 18% water) at a flow rate of 0.8 mL/min at 40 °C. Room temperature refers to 25(±1)°C.

NMR spectra were acquired on a Varian 500 (^1H 500 MHz, ^{13}C 126 MHz) or a Varian 300 (^1H 300 MHz, ^{13}C 75 MHz) NMR spectrometer. The residual solvent peaks were used as the internal reference. Chemical shifts (δ) are reported in ppm. The following abbreviations are used to indicate the multiplicity in ^1H NMR spectra: s, singlet; d, doublet; t, triplet; m, multiplet. ^{13}C NMR spectra were acquired in a broadband decoupled mode. All NMR spectra were recorded at 30°C.

UV–vis absorption spectroscopy was executed on a Jasco V-750 or Perkin-Elmer 465 spectrophotometers. Data were collected from 800 to 200 nm using a 0.5 nm data interval and a 400 nm/s scan speed. Hellma Analytics high-precision quartz cuvettes were used with an optical path length of 1.0 cm. Spectra were recorded in 3×10^{-5} M solutions at 25(±1)°C. Baseline correction was used for each solvent.

High-resolution mass spectrometry measurements were performed on a Sciex TripleTOF 5600+ high-resolution tandem mass spectrometer equipped with a DuoSpray ion source. APCI or ESI ionization was applied in positive ion detection mode. Samples were dissolved in acetonitrile and flow injected into the acetonitrile/water 1:1 flow. The flow rate was 0.2 mL/min. The resolution of the mass spectrometer was 35,000.

S2 Experimental Protocols and Product Characterization

S2.1 Synthetic overview for the targeted dithienylethenes

Our synthetic approach to perhydro-cycloalkene derived diarylethenes is shown in Fig. S1. First, chlorine as potential functional handle for late-stage modification was introduced via conventional chlorination of thiophene **S1**. Subsequently, diketones **3 - 6** were prepared in a Friedel-Crafts acylation in the presence of the corresponding acyl chloride (**S3 - S6**) and Lewis acid catalyst ($AlCl_3$). Acyl chlorides were previously accessed from the carboxylic acid derivatives using either thionyl chloride or oxalyl chloride. The final carbonyl coupling step was performed under optimized McMurry conditions with the use of $TiCl_4$(*collidine*) complex for practical simplicity and enhanced reactivity.

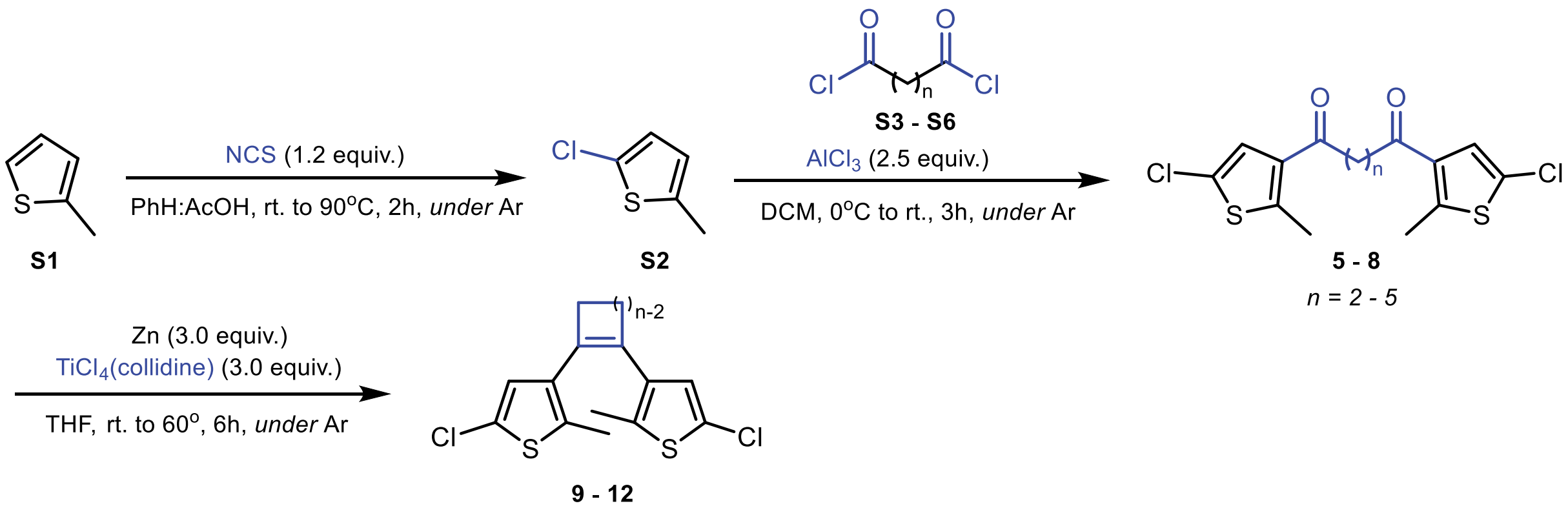

Fig. S1. General synthetic route for the targeted dithienylethenes.

S2.2 General Procedure A: Synthesis of dicarboxylic acid chlorides

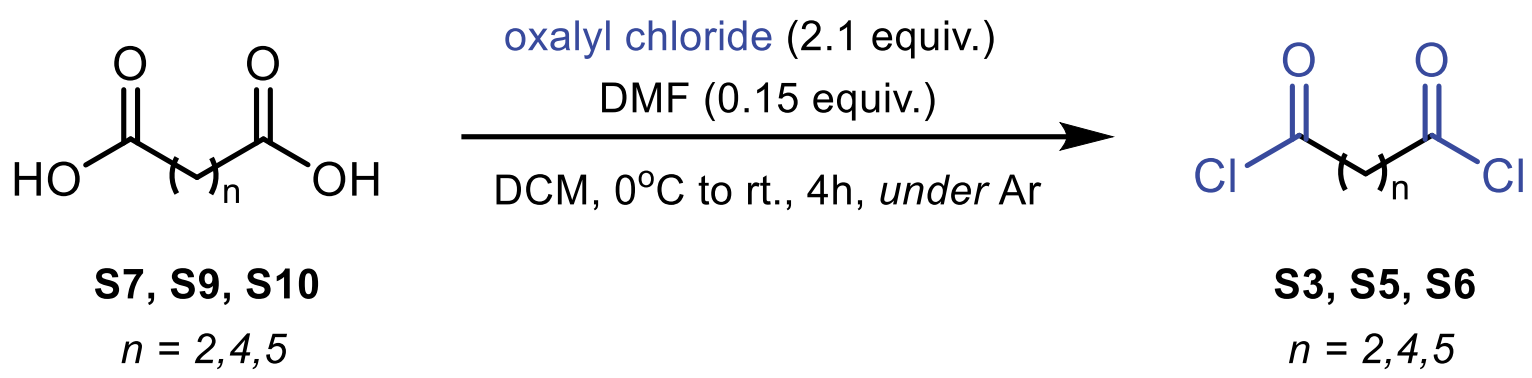

A stirred solution of the dicarboxylic acid (**S7**, **S9**, **S10**, 50.0 mmol) in DCM (20 mL) was cooled to 0°C. Oxalyl chloride (105 mmol, 2.1 equiv.) and DMF (7.50 mmol, 0.15 equiv.) were added portionwise. After stirring for 30 minutes the mixture was allowed to warm to room temperature and was stirred for a total of 4 hours. After completion the mixture was concentrated under reduced pressure to afford crude acyl chlorides **S3**, **S5**, and **S6** as oils. These products were used in the next step without further purification.

[Note]: Dicarboxylic acid chlorides (**S3** – **S6**) should be stored in dry, dark medium to avoid unwanted decomposition/discoloration. Glutaryl chloride (**S4**), if treated properly, can be stored for up to 6 months in a freezer without significant loss of purity due to hydrolysis.

S2.3 Synthesis of glutaryl chloride

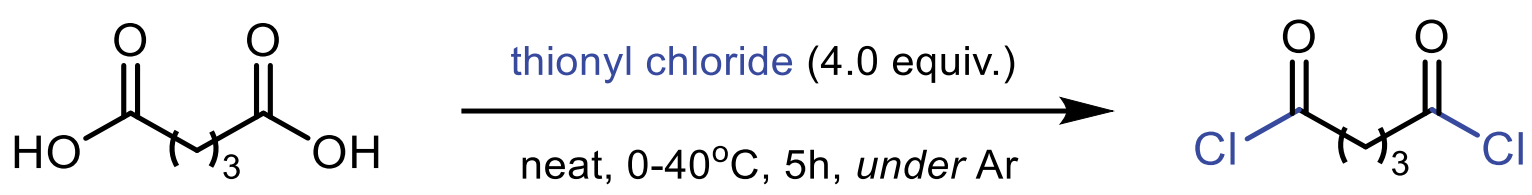

S8 **S4**

An oven-dried 250 mL round-bottom flask was charged with glutaric acid (**S8**) (10.0 g, 76 mmol) under argon. Subsequently, thionyl chloride (36.0 g, 300 mmol, 4.0 equiv.) at 0°C and the resulting mixture was heated to 40°C under rigorous stirring. After 5 hours the mixture was concentrated under reduced pressure (using a water aspirator). The remaining crude oil was dissolved in DCM and was again concentrated under vacuo using a rotary evaporator. The crude product was further purified by vacuum distillation (10 mbar, bp. 84°C, at 120°C oil temp.) yielding the desired product as a colorless oil (9.6 g, 57 mmol, 75%).

S2.4 Synthesis of 2-chloro-5-methylthiophene

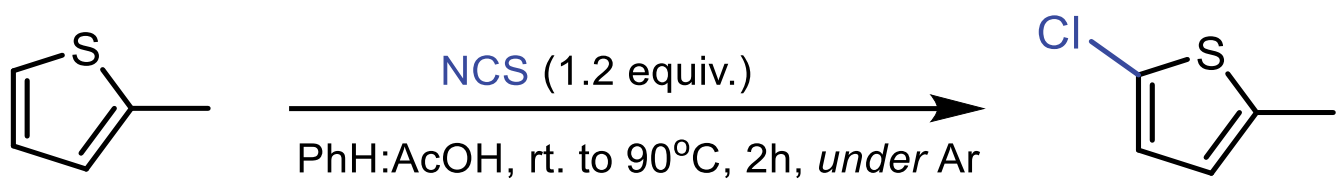

S1 **S2**

A 100 mL round-bottom flask was charged with 2-methylthiophene (**S1**) (10.0 g, 102 mmol) and was dissolved in a mixture of benzene (40 mL) and glacial acetic acid (40 mL). N-chlorosuccinimide (14.96 g, 112 mmol, 1.1 equiv.) was added as one portion, and the mixture was left at ambient temperature under rigorous stirring for 30 minutes. Subsequently, the mixture was heated to reflux temperature (90°C) and was stirred for a further 1 hour. After completion based on TLC monitoring) the mixture was added to an aqueous solution of NaOH (30 mL, 3 M) and was extracted with EtOAc (3 x 30 mL). The combined organic phase was washed with 1M NaOH solution, brine and was dried over anhydrous $MgSO_4$. After evaporation of the solvent under reduced pressure the crude oil was further purified by atmospheric distillation (bp. 155°C, at 180°C oil temp.) affording the desired product as a colorless oil (9.65 g, 72.8 mmol, 71%).

[Note]: Use of toluene over benzene ass solvent should be avoided as it makes separation during atmospheric distillation more challenging.

^{1}H NMR ($CDCl_3$, 500 MHz): δ = 6.69 (d, *J* = 3.6 Hz, 1H), 6.50-6.54 (m, 1H), 2.41 (s, 3H) ppm. (see Spectrum)

^{13}C NMR ($CDCl_3$, 125.5 MHz): δ = 138.7, 126.7, 126.0, 124.5, 15.6 ppm. (see Spectrum)

EI-MS (*m/z, relative intensity*): 132 (M^+, 73), 97 (100), 134 (26), 133 (24).

Rf: 0.78 (hexanes)

HRMS (APCI-TOF): *m/z* calcd. for $[C_5H_5ClS]^+$ ($[M]^+$) 131.9795, found 131.9779.

S2.5 General Procedure B: Synthesis of diketones via Friedel-Crafts acylation

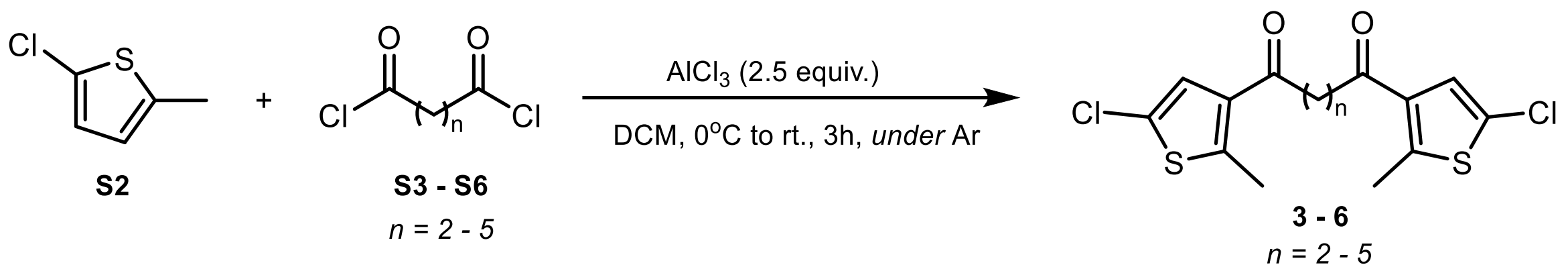

An oven-dried 250 mL round-bottom flask was charged with 2-chloro-5-methylthiophene (0.5 equiv.), anhydrous DCM (0.8 M) and dicarboxylic acid chloride (**S3 – S6**) (1.0 equiv.), respectively. The solution was cooled to 0°C and $AlCl_3$ (2.5 equiv.) was added in portions under rigorous stirring. Subsequently, the mixture was stirred at 0°C for 30 minutes then was allowed to warm to room temperature over the course of 2 hours. After completion (based on TLC monitoring) the mixture was poured over ice and was extracted with DCM. The combined organic phase was washed with brine and was dried over anhydrous $MgSO_4$. The solvents were removed under reduced pressure and the crude product was further purified by column chromatography (SiO_2, hexanes / EtOAc) affording the desired diketones as yellow-white crystalline solids.

[Note 1]: If column chromatography was performed on large scales (> 2 g) crystalline product precipitated during running the method on some occasions. This can lead to clogging in the tubing and accordingly should be avoided.

[Note 2]: In case of reversed order of reagent addition (pre-stir $AlCl_3$ and acyl chloride, then addition of thiophene **S2**) we experienced decreased yields, compared to *General Procedure B* described above.

[Note 3]: Diketone products **3 – 6** could be stained with DNP on TLC, resulting in characteristic orange spots upon gentle heating.

S2.6 Preparation of $TiCl_4$(collidine) complex reagent[1]

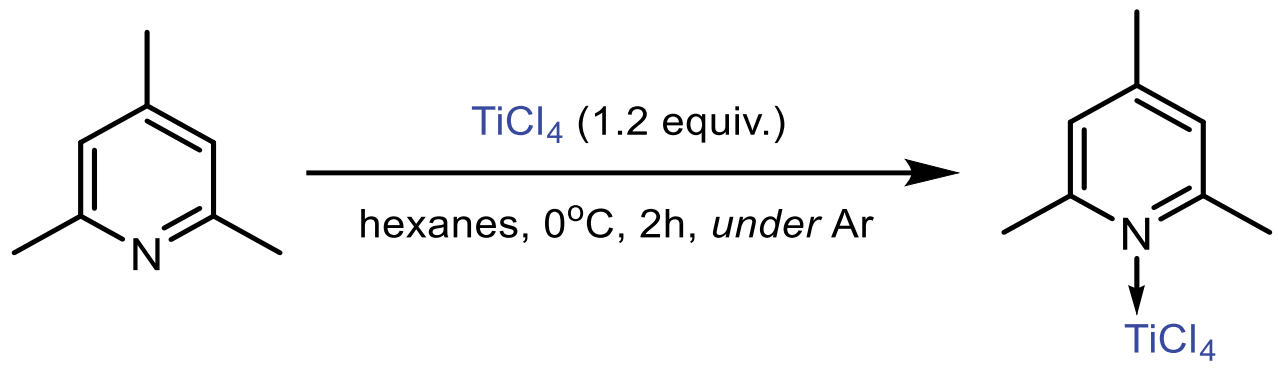

2 **1**, 87%

An oven-dried three-neck round-bottom flask (fitted with thermometer, reflux condenser, addition funnel) was charged with anhydrous hexanes (170 mL) and $TiCl_4$ (10.0 mL, 17.3 g, 91.2 mmol, 1.0 equiv.), respectively. The mixture was cooled to 0°C and 2,4,6-trimethylpyridine (**2**) (collidine) (14.5 mL, 13.3 g, 109 mmol, 1.2 equiv.) was added dropwise using the addition funnel. Following the addition, formation of a yellow precipitate was visible, and the resulting suspension was stirred for a further 1 hour. After completion the precipitate was filtered, washed with dry hexanes and was transferred to a round-bottom flask to dry the solid under high-vacuum affording the product as a yellow powder (24.6 g, 87%).

[Note 1]: Although bench-stable, $TiCl_4$(*collidine*) should be stored in a dry container, under inert (Ar or N_2) atmosphere to avoid unwanted hydrolysis. $TiCl_4$(*collidine*), if treated properly, can be stored for up to 9 months in a freezer without signs of degradation/hydrolysis.

[Note 2]: The yellow color of the complex can be attributed to the yellowish, discolored $TiCl_4$ reagent. These trace contaminants could not be efficiently removed.

S2.7 General Procedure C: McMurry cyclization using $TiCl_4$(collidine) complex

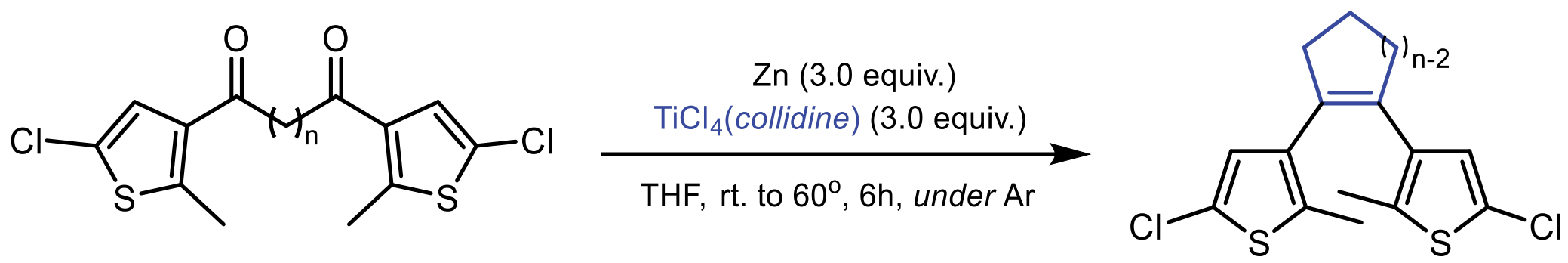

3 - 6
n = 2 - 5

7 - 10

An oven-dried scintillation vial was charged with $TiCl_4$(*collidine*) (**1**) (3.0 equiv.) and zinc powder (3.0 equiv.) and was suspended in anhydrous THF (0.5 M). The resulting mixture was refluxed at 60°C for 5 hours before the addition of **3 – 6** (1.0 equiv.), which was followed by a further 12 hours of stirring at 60°C. After completion the reaction mixture was quenched with sat. Na_2CO_3 solution, filtered (to remove titanium salt precipitates) and extracted with diethyl ether. The combined organic phase was washed with brine, dried over anhydrous $MgSO_4$ and was concentrated under reduced pressure. The crude product was further purified by column chromatography (SiO_2, hexanes) affording the desired products as yellow-white crystalline solids.

S2.8 General Procedure D: McMurry cyclization using $TiCl_4$

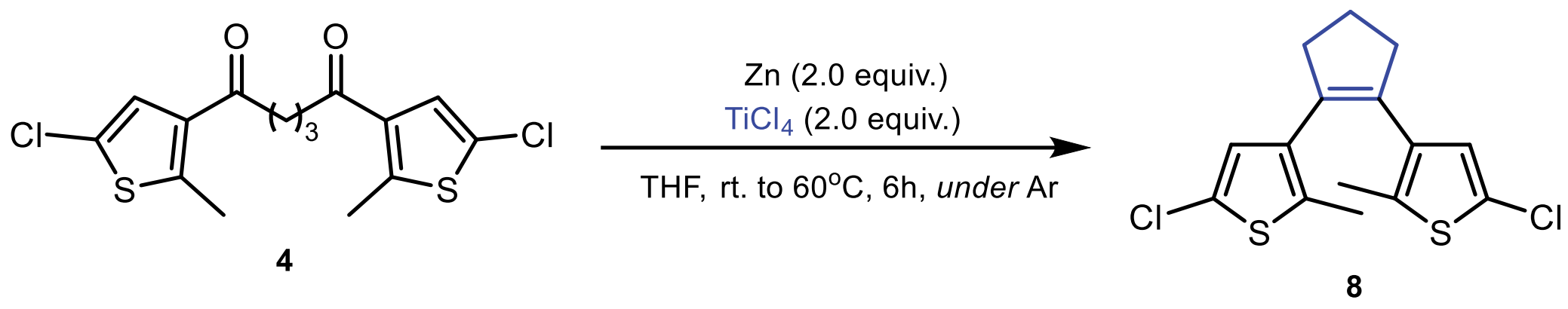

An oven-dried 20 mL scintillation vial was charged with zinc (36.2 mg, 0.55 mmol, 2.0 equiv.) and was suspended in anhydrous THF (1.5 mL). To this suspension was added $TiCl_4$ (157 mg, 91 µL, 0.83 mmol, 3.0 equiv.) and the mixture was refluxed at 60°C for 45 minutes. Subsequently, **4** (100.0 mg, 0.27 mmol, 1.0 equiv.) was added and the mixture was stirred for a further 2 hours at the same temperature. After completion (based on TLC monitoring) the reaction was quenched with sat. Na_2CO_3 solution (2 mL), filtered (to remove titanium salt precipitates) and extracted with diethyl ether (3 x 10 mL). The combined organic phase was washed with brine, dried over anhydrous $MgSO_4$ and was concentrated under reduced pressure. The crude product was further purified by column chromatography (SiO_2, hexanes) affording the desired product as yellow-white crystalline solid (37.4 mg, 41%).

[Note]: Handling of $TiCl_4$ requires ubiquitous precautions. $TiCl_4$ is a volatile, toxic and corrosive liquid. It reacts with water violently to release HCl. We highly recommend using a luer lock syringe for transferring to reduce danger of spilling due to pressure build up in the syringe.

S2.9 Scale-dependence of the isolated yields in the McMurry reaction

Table S1. Isolated yields of the McMurry reaction of **6** on different scales using different reagents.

$TiCl_4$ [0.3 mmol]	*$TiCl_4$ [7.75 mmol]*	*$TiCl_4$ [12.5 mmol]*	*$TiCl_4$(collidine) [0.3 mmol]*	*$TiCl_4$(collidine) [2.0 mmol]*	*$TiCl_4$(collidine) [5.5 mmol]*
40%	***63%***	***70%***	***83%***	***83%***	***63%***

When using $TiCl_4$ yellow precipitation and white smoke was often apparent even in case of using oven-dried glassware under an inert atmosphere. The $TiCl_4$ reagent cannot be stored/purchased in a sealed (rubber septum) compartment, therefore these hydrolytic processes could not be completely avoided. This becomes a significant difficulty when working on small scales, where a very small volume of volatile liquid is needed. As highlighted above, this lowers reaction yields by up to 30%. On the other

hand, $TiCl_4$(*collidine*) offers significant ease of handling and storing, while it performs with the same (or even better) efficiency under the McMurry coupling conditions.

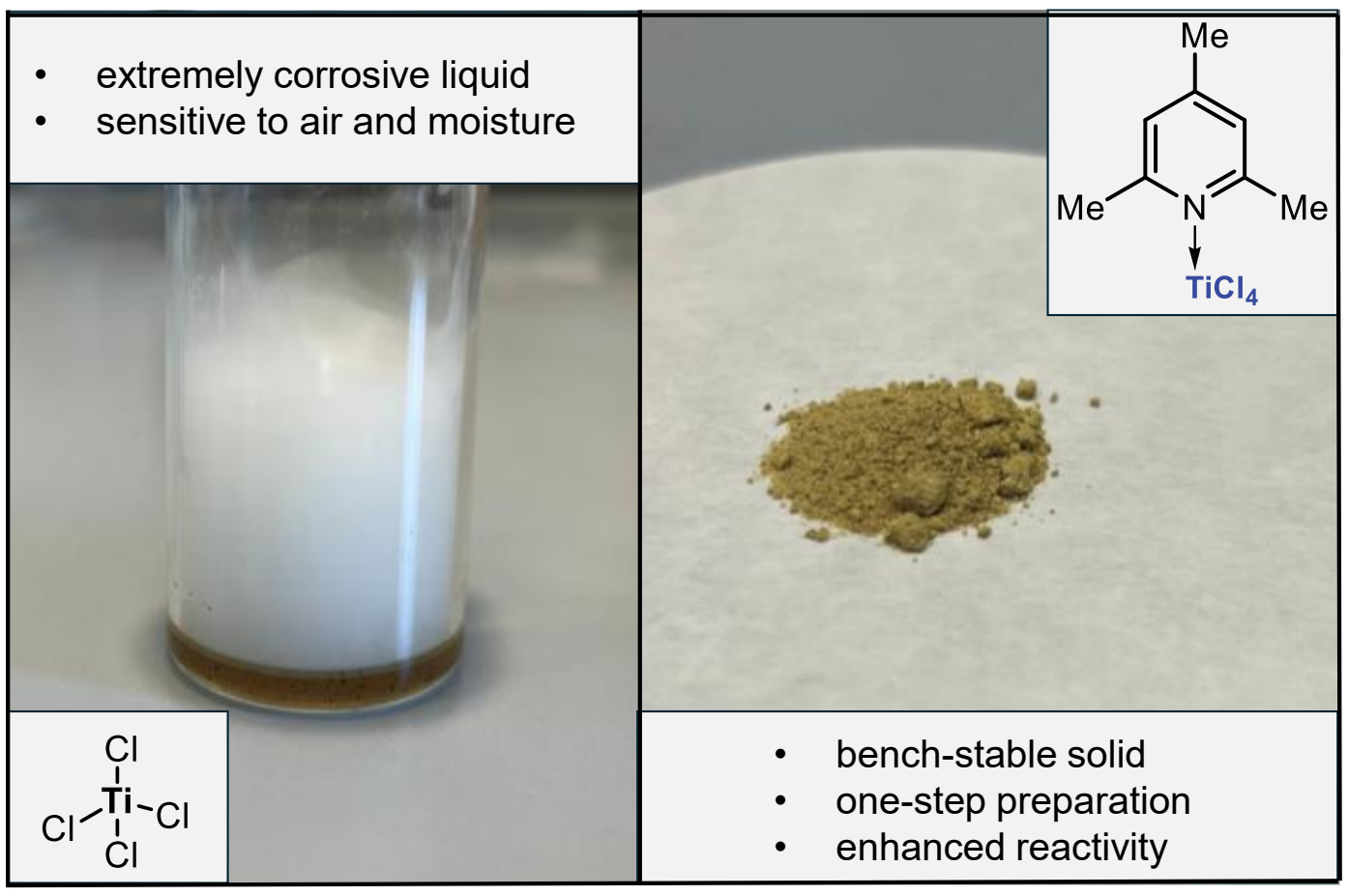

Fig. S2. Comparison of $TiCl_4$ and $TiCl_4$(*collidine*) reagents' physical properties.

S2.10 Use of $TiCl_4$(collidine) results in cleaner crude product mixtures

We also found that the use of $TiCl_4$(*collidine*) over $TiCl_4$ results in a cleaner crude product mixture as illustrated in Fig. S3. This feature of the reaction is remarkably beneficial as purification via column chromatography is performed in 100% hexanes so conditions for separation are limited.

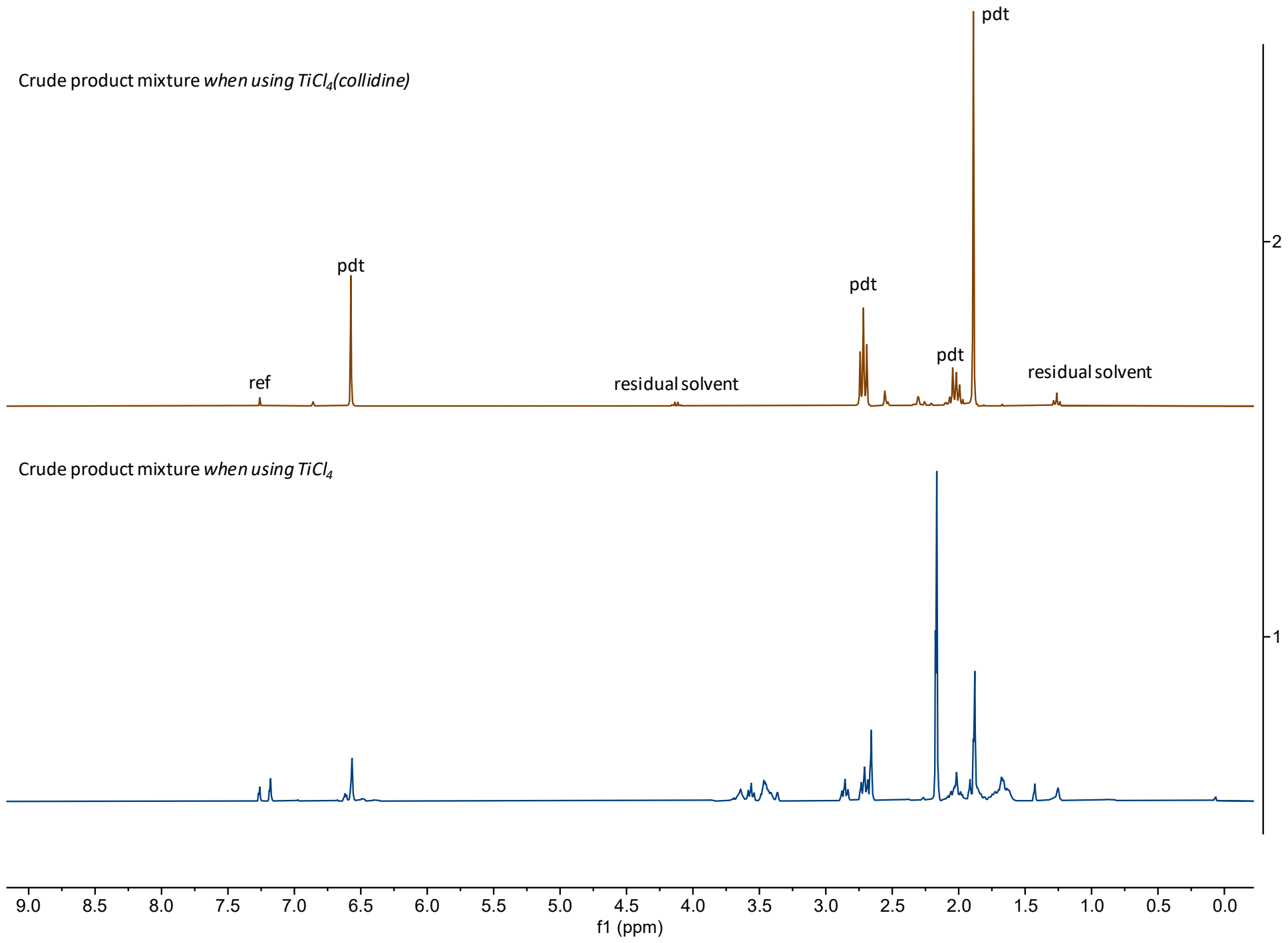

Fig. S3. Comparison of crude product mixture ^{1}H NMR spectra when using $TiCl_4$(*collidine*) vs $TiCl_4$.

S2.11 Product characterization

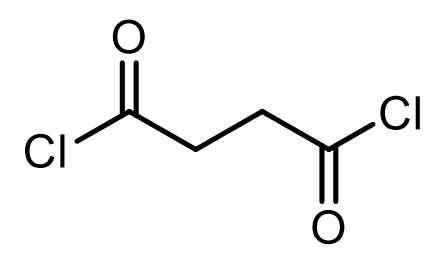

S3
succinyl dichloride

Compound S3: Prepared from succinic acid (**S7**) according to *General Procedure A*. Crude product was used without further purification. **Yield:** *estimated quant*.

Physical state: yellow oil

^{1}H NMR ($CDCl_3$, 500 MHz): δ = 3.26 (s, 4H) ppm. (see Spectrum)

^{13}C NMR ($CDCl_3$, 125.5 MHz): δ = 172.1, 41.7 ppm. (see Spectrum)

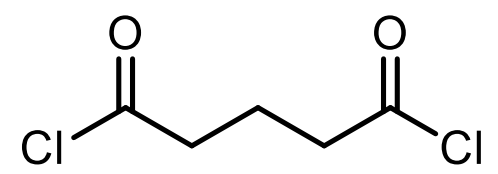

S4
glutaroyl dichloride

Compound S4: Prepared from glutaric acid (**S8**) according to *S2.3* (*Synthesis of glutaryl chloride*). Crude product was further purified by vacuum distillation (*see details above: S2.3*). **Yield:** 75%.

Physical state: colorless oil

^{1}H NMR ($CDCl_3$, 500 MHz): δ: 3.01 (t, J = 7.04 Hz, 4H), 2.07 (p, J = 7.05 Hz, 2H) ppm. (see Spectrum)

^{13}C NMR ($CDCl_3$, 125.5 MHz): δ: 173.21, 45.21, 20.49 ppm. (see Spectrum)

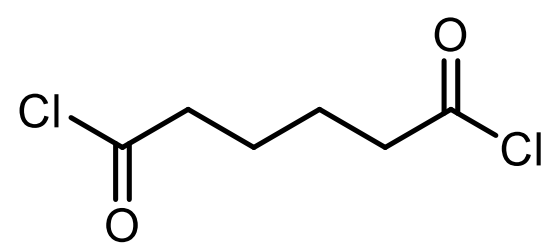

S5
adipoyl dichloride

Compound S5: Prepared from adipic acid (**S9**) according to *General Procedure A*. Crude product was used without further purification. **Yield:** *estimated quant*.

Physical state: yellow oil

[Note]: Clean NMR spectra were unattainable due to hydrolytic instability.

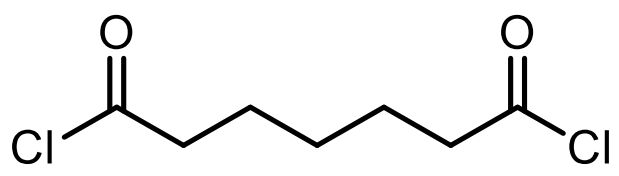

S6
heptanedioyl dichloride

Compound S6: Prepared from pimeric acid (**S10**) according to *General Procedure A*. Crude product was used without further purification. **Yield:** *estimated quant*.

Physical state: pale-yellow oil

[Note]: Clean NMR spectra were unattainable due to hydrolytic instability.

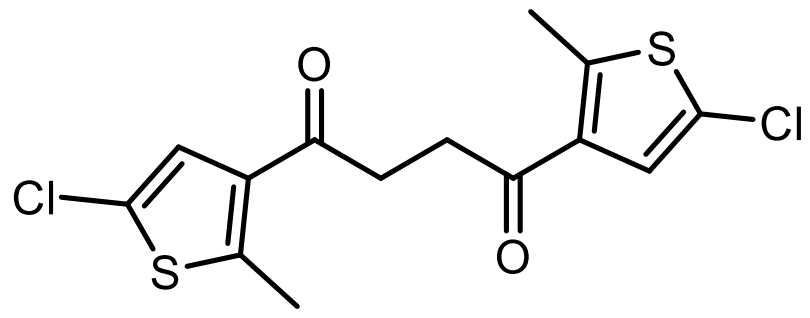

3
1,4-bis(5-chloro-2-methylthiophen-3-yl)butane-1,4-dione

Compound 3: Prepared according to *General Procedure B*. Crude product was further purified by column chromatography (SiO_2, hex/EtOAc, 9:1). **Yield:** 23%.

Physical state: pale-pink crystalline solid

^{1}H NMR ($CDCl_3$, 500 MHz): δ = 7.27 (s, 2H), 3.15 (s, 4H), 2.65 (s, 6H) ppm. (see Spectrum)

^{13}C NMR ($CDCl_3$, 125.5 MHz): δ = 193.6, 148.0, 134.9, 127.0, 125.6, 35.3, 16.1 ppm. (see Spectrum)

Rf: 0.47 (hex/EtOAc, 9:1)

HRMS (APCI-TOF): *m/z* calcd. for $[C_{14}H_{13}Cl_2S_2O_2]^+$ ($[M+H]^+$) 346.9734, found 346.9750.

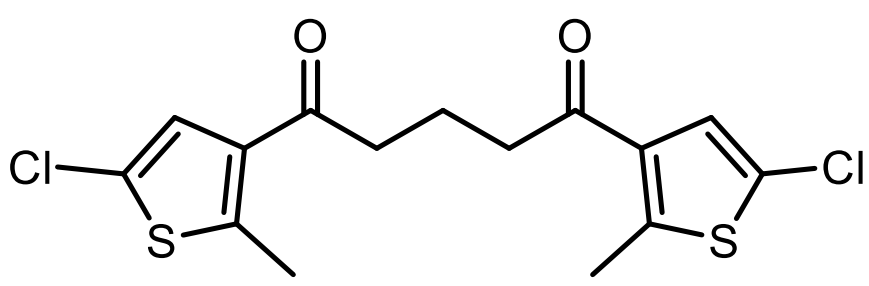

4
1,5-bis(5-chloro-2-methylthiophen-3-yl)pentane-1,5-dione

Compound 4: Prepared according to *General Procedure B*. Crude product was further purified by column chromatography (SiO_2, hex/EtOAc, 9:1). **Yield:** 52%

Physical state: white crystalline solid

^{1}H NMR ($CDCl_3$, 500 MHz): δ = 7.10 (s, 2H), 2.79 (t, J = 6.9 Hz, 2H), 2.57 (s, 6H), 1.99 (hept, J = 6.9 Hz, 2H) ppm. (see Spectrum)

^{13}C NMR ($CDCl_3$, 125.5 MHz): δ = 194.7, 147.5, 134.8, 126.8, 125.1, 40.4, 18.0, 15.9 ppm. (see Spectrum)

Rf: 0.41 (hex/EtOAc, 9:1)

HRMS (APCI-TOF): *m/z* calcd. for $[C_{15}H_{15}Cl_2S_2O_2]^+$ ($[M+H]^+$) 360.9890, found 360.9864.

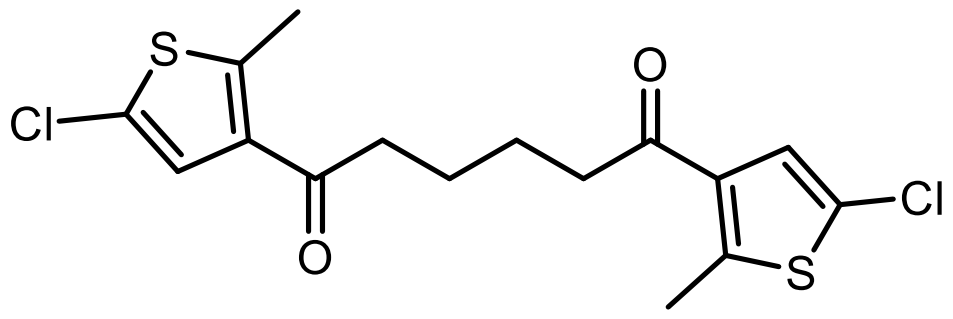

5
1,6-bis(5-chloro-2-methylthiophen-3-yl)hexane-1,6-dione

Compound 5: Prepared according to *General Procedure B.* Crude product was further purified by column chromatography (SiO_2, hex/EtOAc, 9:1). **Yield:** 22%.

Physical state: white crystalline solid

^{1}H NMR ($CDCl_3$, 500 MHz): δ = 7.15 (s, 2H), 2.80 (m, 4H), 2.65 (s, 6H), 1.73 (m, 4H) ppm. (see Spectrum)

^{13}C NMR ($CDCl_3$, 125.5 MHz): δ = 195.3, 147.7, 135.1, 126.9, 125.4, 41.6, 23.6, 16.1 ppm. (see Spectrum)

Rf: 0.38 (hex/EtOAc, 9:1)

HRMS (ESI-TOF): *m/z* calcd. for $[C_{16}H_{16}Cl_2S_2O_2Na]^+$ ($[M+Na]^+$) 396.9861, found 396.9854.

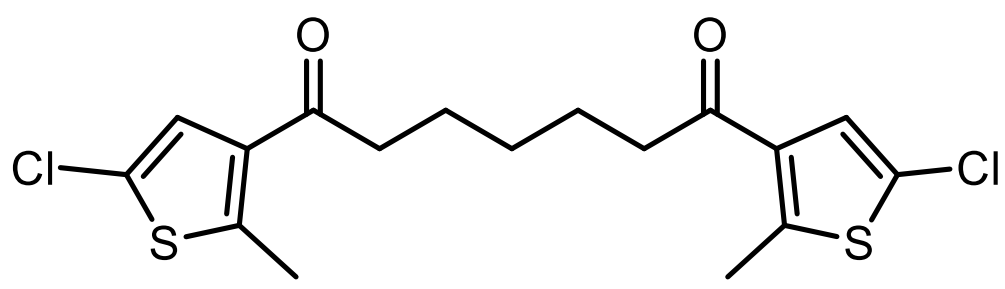

6
1,7-bis(5-chloro-2-methylthiophen-3-yl)heptane-1,7-dione

Compound 6: Prepared according to *General Procedure B.* Crude product was further purified by column chromatography (SiO_2, hex/EtOAc, 9:1). **Yield:** 65%.

Physical state: white crystalline solid

^{1}H NMR ($CDCl_3$, 500 MHz): δ = 7.14 (s, 2H), 2.75 (t, J = 7.25 Hz, 4H), 2.63 (s, 6H), 1.70 (p, J = 7.38 Hz, 4H), 1.41 (m, 2H) ppm. (see Spectrum)

^{13}C NMR ($CDCl_3$, 125.5 MHz): δ = 195.5, 147.6, 135.2, 126.9, 125.3, 41.5, 28.9, 23.8, 16.1 ppm. (see Spectrum)

Rf: 0.49 (hex/EtOAc, 9:1)

HRMS (APCI-TOF): *m/z* calcd. for $[C_{17}H_{19}Cl_2S_2O_2]^+$ ($[M+H]^+$) 389.0203, found 389.0188.

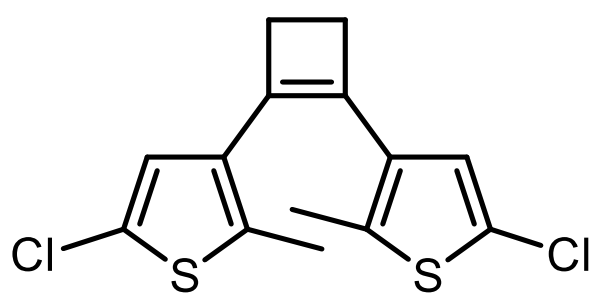

7
1,2-bis(5-chloro-2-methylthiophen-3-yl)cyclobut-1-ene

Compound 7: Prepared according to *General Procedure C.* Crude product was further purified by column chromatography (SiO_2, hexanes). **Yield:** 46%.

Physical state: yellow, amorphous solid

^{1}H NMR ($CDCl_3$, 500 MHz): δ = 6.68 (s, 2H), 2.79 (s, 4H), 2.25 (s, 6H) ppm. (see Spectrum)

^{13}C NMR ($CDCl_3$, 125.5 MHz): δ = 134.7, 134.6, 133.6, 126.2, 125.7, 30.1, 14.4 ppm. (see Spectrum)

Rf: 0.68 (hexanes)

HRMS (APCI-TOF): *m/z* calcd. for $[C_{14}H_{12}S_2Cl_2]^+$ ($[M+H]^+$) 313.9757, found 313.9735.

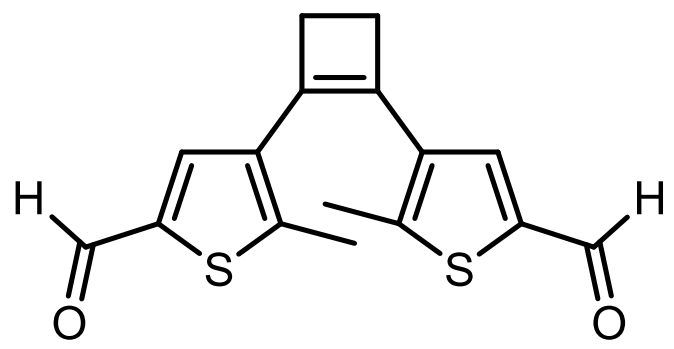

11

4,4'-(cyclobut-1-ene-1,2-diyl)bis(5-methylthiophene-2-carbaldehyde)

Compound 11: A 20 mL oven-dried scintillation vial was charged with **7** (100 mg, 0.31 mmol, 1.0 equiv.) and was dissolved in anhydrous THF (2 mL) under Argon atmosphere. The solution was cooled to 0°C and *n*-butyllithium (0.28 mL, 2.5 M, 0.7 mmol, 2.2 equiv.) was added dropwise resulting in a dark green solution, which was stirred for a further 60 minutes. Subsequently, anhydrous DMF (92.8 mg, 98 µL, 1.27 mmol, 4.0 equiv.) was added slowly and the mixture turned brown-yellow. After stirring the mixture for 4 hours at room temperature the reaction was quenched with the addition of water (5 mL) and was extracted with diethyl ether (3 x 10 mL). The combined organic phase was dried over $MgSO_4$ and concentrated under reduced pressure affording the crude product as a yellow solid. This residue was further purified by column chromatography (SiO_2, hex/EtOAc). **Yield:** 66%.

Physical state: yellow, amorphous solid

^{1}H NMR ($CDCl_3$, 500 MHz): δ = 9.77 (s, 2H), 7.54 (s, 2H), 2.92 (s, 4H), 2.43 (s, 6H) ppm. (see Spectrum)

^{13}C NMR ($CDCl_3$, 125.5 MHz): δ = 182.6, 147.4, 140.4, 136.5, 135.7, 135.1, 30.2, 15.6 ppm. (see Spectrum)

Rf: 0.18 (hex/EtOAc, 9:1)

HRMS (APCI-TOF): *m/z* calcd. for $[C_{16}H_{15}S_2O_2]^+$ ($[M+H]^+$) 303.0507, found 303.0495.

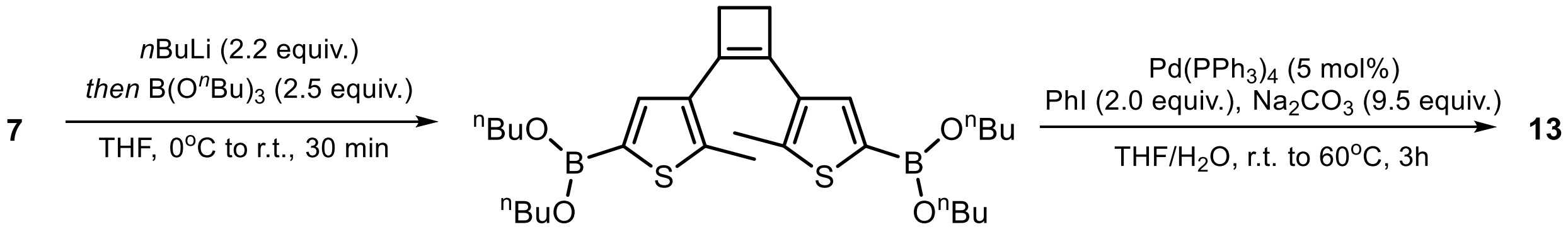

12

not isolated to avoid deborylation

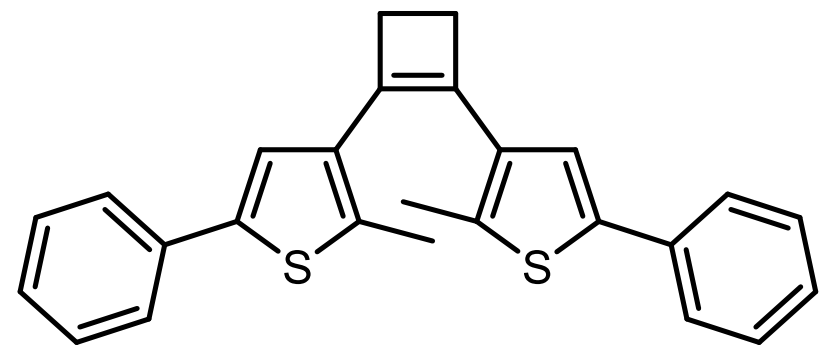

13

1,2-bis(2-methyl-5-phenylthiophen-3-yl)cyclobut-1-ene

Compound 13: A 20 mL oven-dried scintillation vial was charged with **7** (100 mg, 0.31 mmol, 1.0 equiv.) and was dissolved in anhydrous THF (2 mL) under inert (Ar) atmosphere. The solution was cooled to 0°C and *n*-butyllithium (0.28 mL, 2.5 M, 0.7 mmol, 2.2 equiv.) was added dropwise resulting in a dark green solution, which was stirred for a further 30 minutes. Subsequently, tributyl borate (183 mg, 0.79 mmol, 2.5 equiv.) was added and the reaction mixture was stirred for a further 60 minutes at room temperature. In a separate 20 mL scintillation vial iodobenzene (130 mg, 0.63 mmol, 2.0 equiv.) was dissolved in THF (1 mL), $Pd(PPh_3)_4$ (18.3 mg, 16 µmol, 5 mol%) was added and the resulting solution was stirred for 15 minutes. Then aqueous Na_2CO_3 solution (1.5 mL, 2 M) and 2 drops of ethylene glycol were added. This two-phase system was heated to 60°C and the solution of the boronic ester was added dropwise, then the reaction mixture was refluxed for a further 3 hours. After completion the mixture was cooled to room temperature and diluted with diethyl ether. The organic layer was separated, washed with brine and dried over $MgSO_4$, then concentrated under reduced pressure. The remaining residue was further purified by column chromatography (SiO_2, hex/EtOAc) affording the desired product as a yellow amorphous solid. **Yield:** 58%.

Physical state: yellow, amorphous solid

^{1}H NMR ($CDCl_3$, 500 MHz): δ = 7.58 (m, 4H), 7.39 (t, *J* = 7.73 Hz, 5H), 7.30 (m, 3H), 2.97 (s, 4H), 2.46 (s, 6H) ppm. (see Spectrum)

^{13}C NMR ($CDCl_3$, 125.5 MHz): δ = 139.9, 135.7, 135.3, 134.8, 134.4, 30.0, 14.5 ppm. (see Spectrum)

Rf: 0.28 (hexanes)

HRMS (APCI-TOF): *m/z* calcd. for $[C_{26}H_{22}S_2]^+$ ($[M]^+$) 398.1157, found 398.1140.

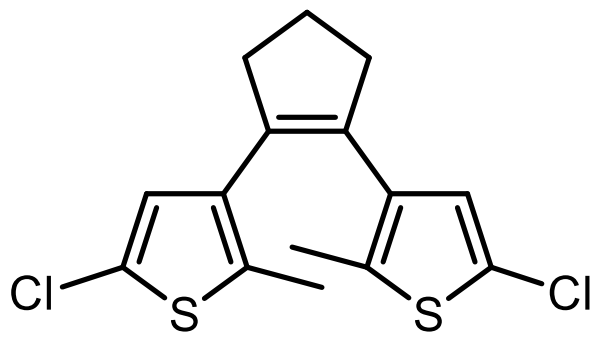

8

1,2-bis(5-chloro-2-methylthiophen-3-yl)cyclopent-1-ene

Compound 8: Prepared according to *General Procedures C* or *D.* Crude product was further purified by column chromatography (SiO_2, hexanes). **Yield:** 83%

Physical state: white crystalline solid

^{1}H NMR ($CDCl_3$, 500 MHz): δ = 6.58 (s, 2H), 2.72 (t, *J* = 7.5 Hz, 4H), 2.05 (p, *J* = 7.37 Hz, 2H), 1.98 (s, 6H) ppm. (see Spectrum)

^{13}C NMR ($CDCl_3$, 125.5 MHz): δ = 135.0, 134.6, 133.4, 126.8, 125.3, 38.5, 23.0, 14.3 ppm. (see Spectrum)

Rf: 0.69 (hexanes)

HRMS (APCI-TOF): *m/z* calcd. for $[C_{15}H_{14}S_2Cl_2]^+$ ($[M]^+$) 327.9913, found 327.9927.

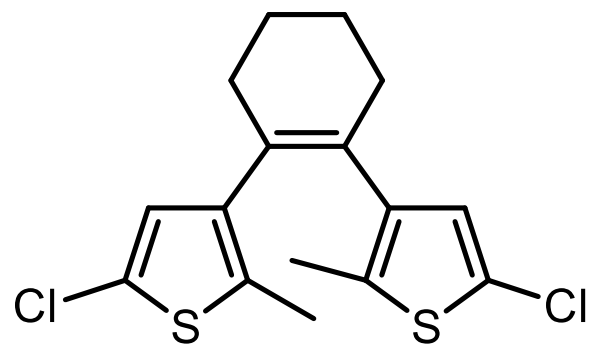

9

1,2-bis(5-chloro-2-methylthiophen-3-yl)cyclohex-1-ene

Compound 9: Prepared according to *General Procedure C.* Crude product was further purified by column chromatography (SiO_2, hexanes). **Yield:** 12%.

Physical state: white crystalline solid

^{1}H NMR ($CDCl_3$, 500 MHz): δ = 6.48 (s, 2H), 2.25 (m, 4H), 1.96 (s, 6H), 1.76 (m, 4H) ppm. (see Spectrum)

^{13}C NMR ($CDCl_3$, 125.5 MHz): δ = 139.4, 132.4, 131.7, 127.2, 124.7, 31.6, 23.1, 14.2 ppm. (see Spectrum)

Rf: 0.64 (hexanes)

HRMS (APCI-TOF): *m/z* calcd. for $[C_{16}H_{17}S_2Cl_2]^+$ ($[M+H]^+$) 343.0148, found 343.0163.

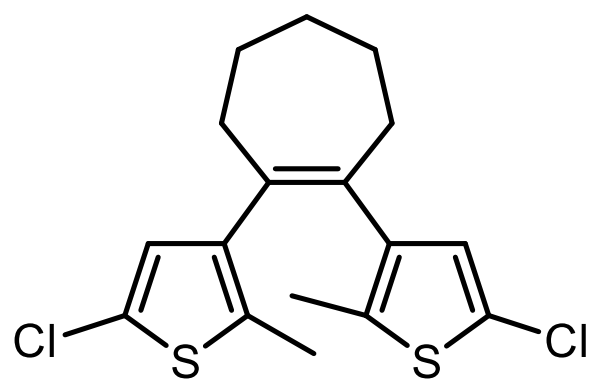

10

1,2-bis(5-chloro-2-methylthiophen-3-yl)cyclohept-1-ene

Compound 10: Prepared according to *General Procedure C.* Crude product was further purified by column chromatography (SiO_2, hexanes). **Yield:** 4%.

Physical state: white crystalline solid

^{1}H NMR ($CDCl_3$, 500 MHz): δ = 6.45 (s, 2H), 2.49 (m, 4H), 1.93 (s, 6H), 1.87 (p, *J* = 5.9 Hz, 2H), 1.63 (p, *J* = 5.9 Hz, 4H) ppm. (see Spectrum)

^{13}C NMR ($CDCl_3$, 125.5 MHz): δ = 140.8, 137.7, 132.1, 127.1, 124.8, 36.0, 32.8, 26.8, 14.3 ppm. (see Spectrum)

Rf: 0.67 (hexanes)

HRMS (APCI-TOF): *m/z* calcd. for $[C_{17}H_{18}S_2Cl_2]^+$ ($[M]^+$) 356.0226, found 356.0230.

S3 UV-Vis analyses

In the following section detailed UV-vis measurements will be displayed featuring effects of solvents, used irradiation wavelengths and times. The spectra *on the left* shows irradiation of open form (open → closed), while spectra shown *on the right* displays irradiation of closed form to induce cycloreversion (closed → open), unless otherwise noted.

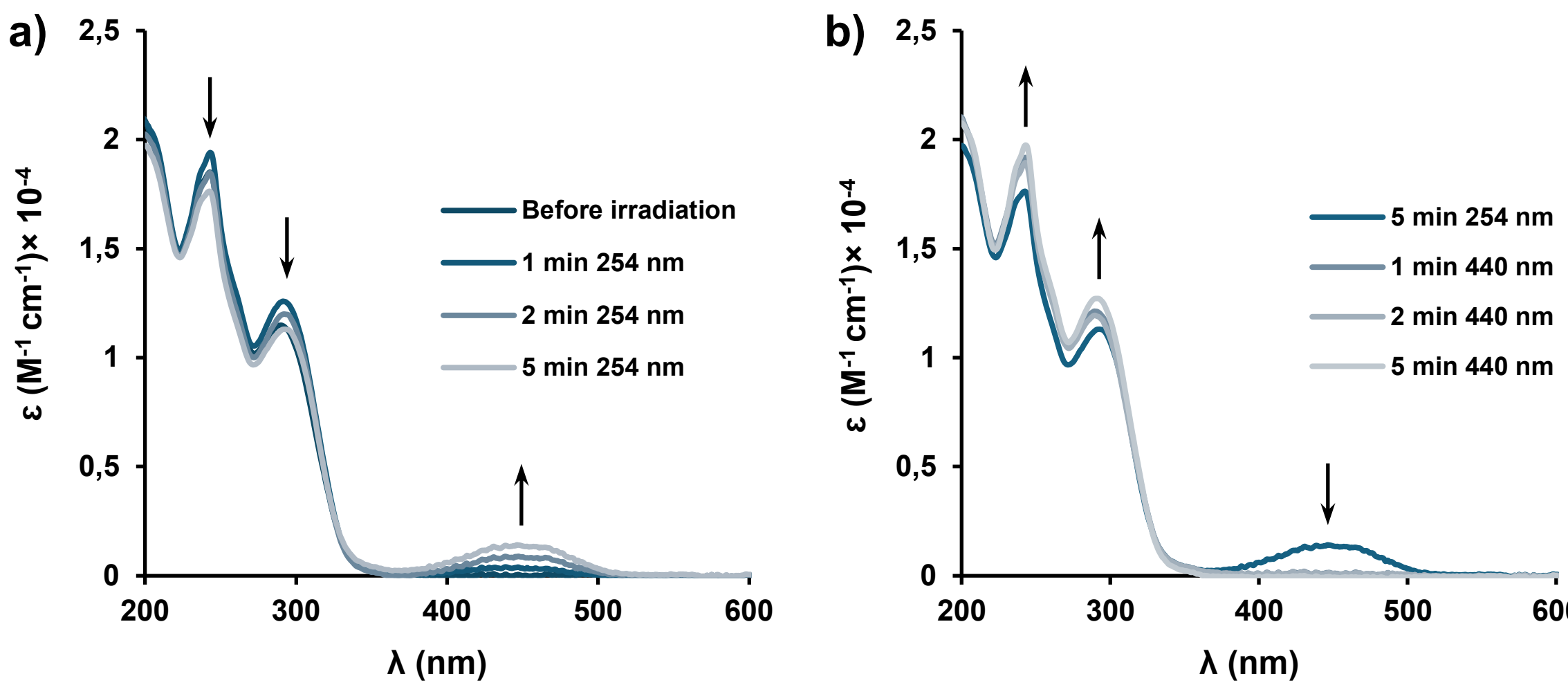

Fig. S4. Changes in the UV-vis spectra of **7** upon irradiation with **a)** UV (λ_{max} = 254 nm) and subsequently with **b)** visible (λ_{max} = 440 nm) light in solution (MeCN, c = 3 × 10^{-5} M).

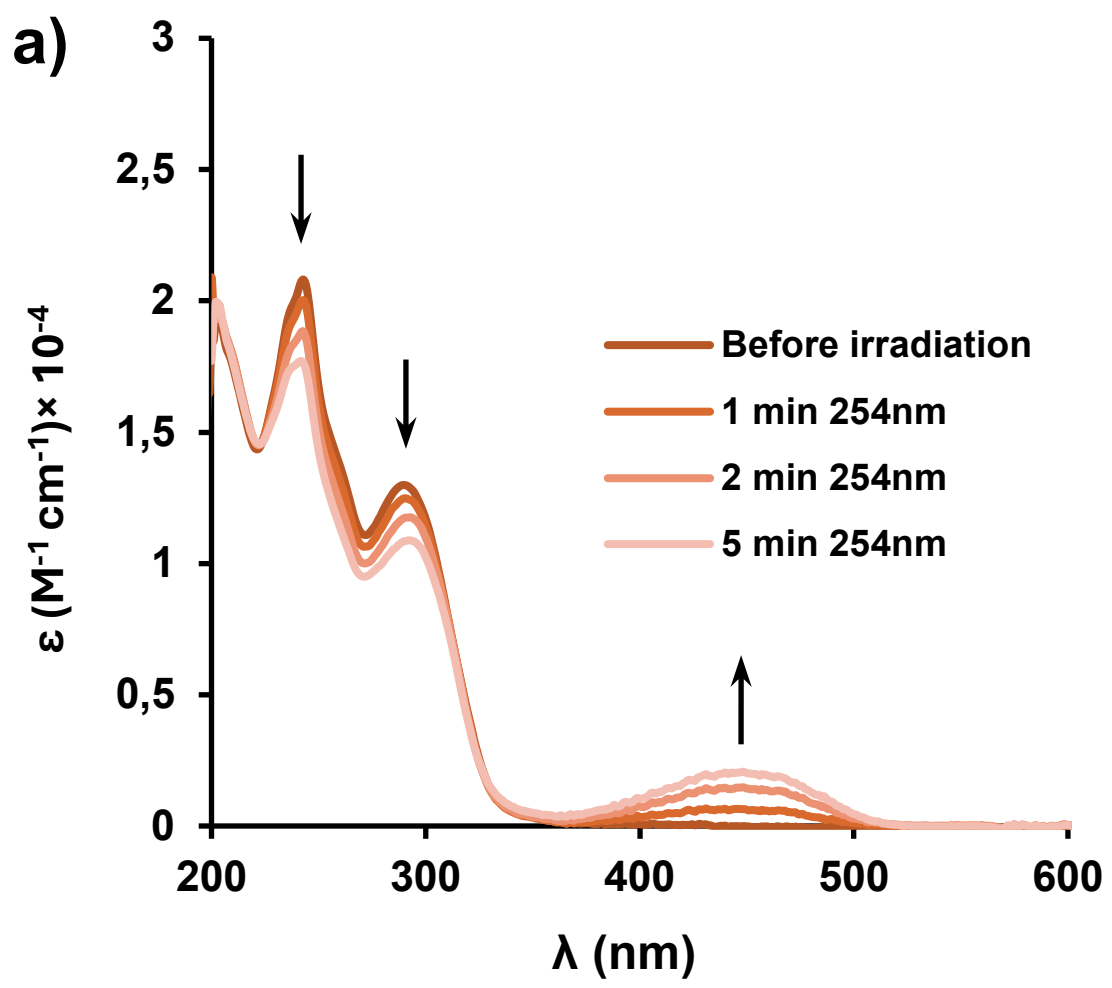

Fig. S5. Changes in the UV-vis spectra of **7** upon irradiation with **a)** UV (λ_{max} = 254 nm) and subsequently with **b)** visible (λ_{max} = 440 nm) light in solution (MeOH, c = 3 × 10^{-5} M).

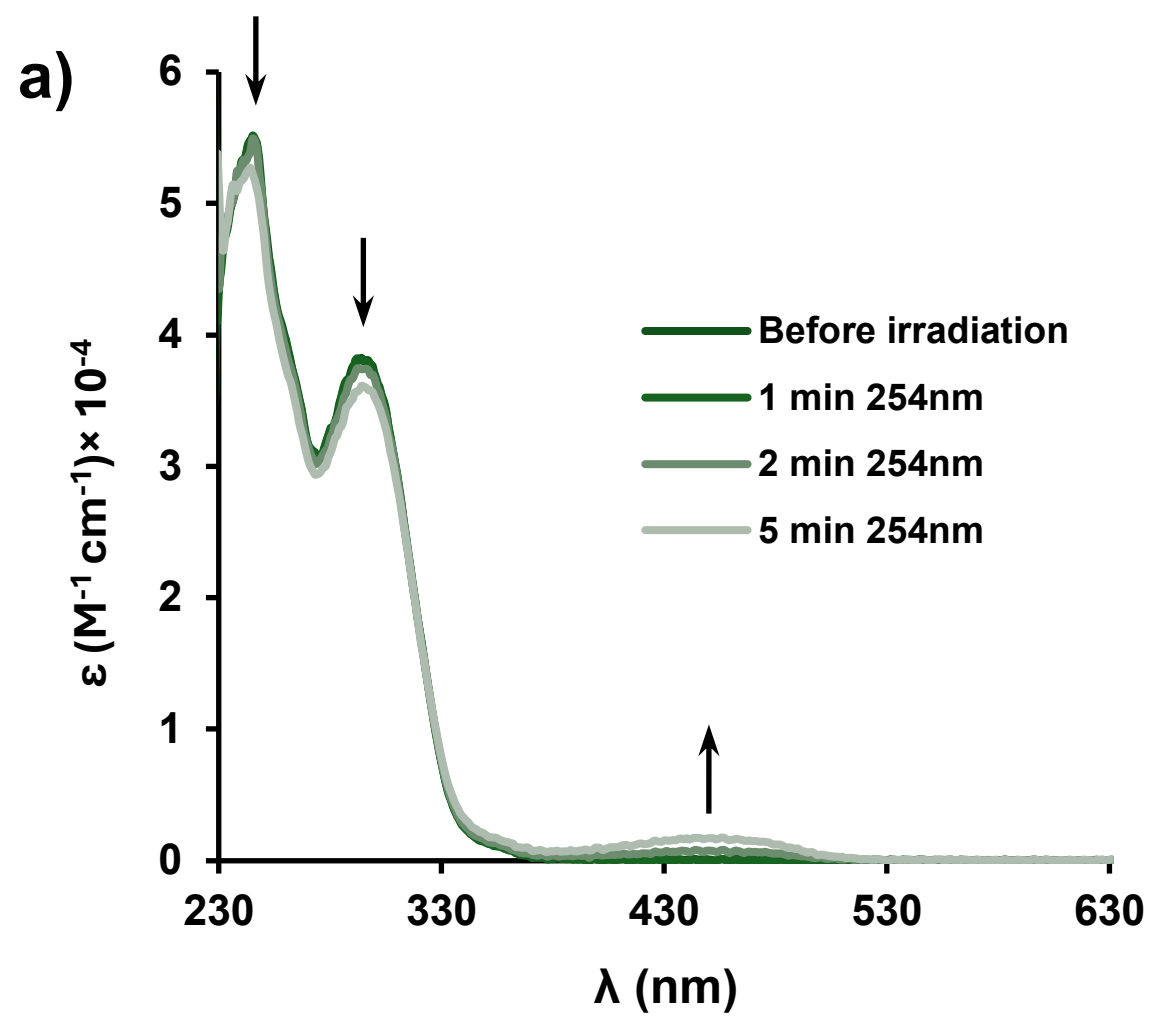

Fig. S6. Changes in the UV-vis spectra of **7** upon irradiation with **a)** UV (λ_{max} = 254 nm) and subsequently with **b)** visible (λ_{max} = 440 nm) light in solution (DCM, c = 3 × 10^{-5} M).

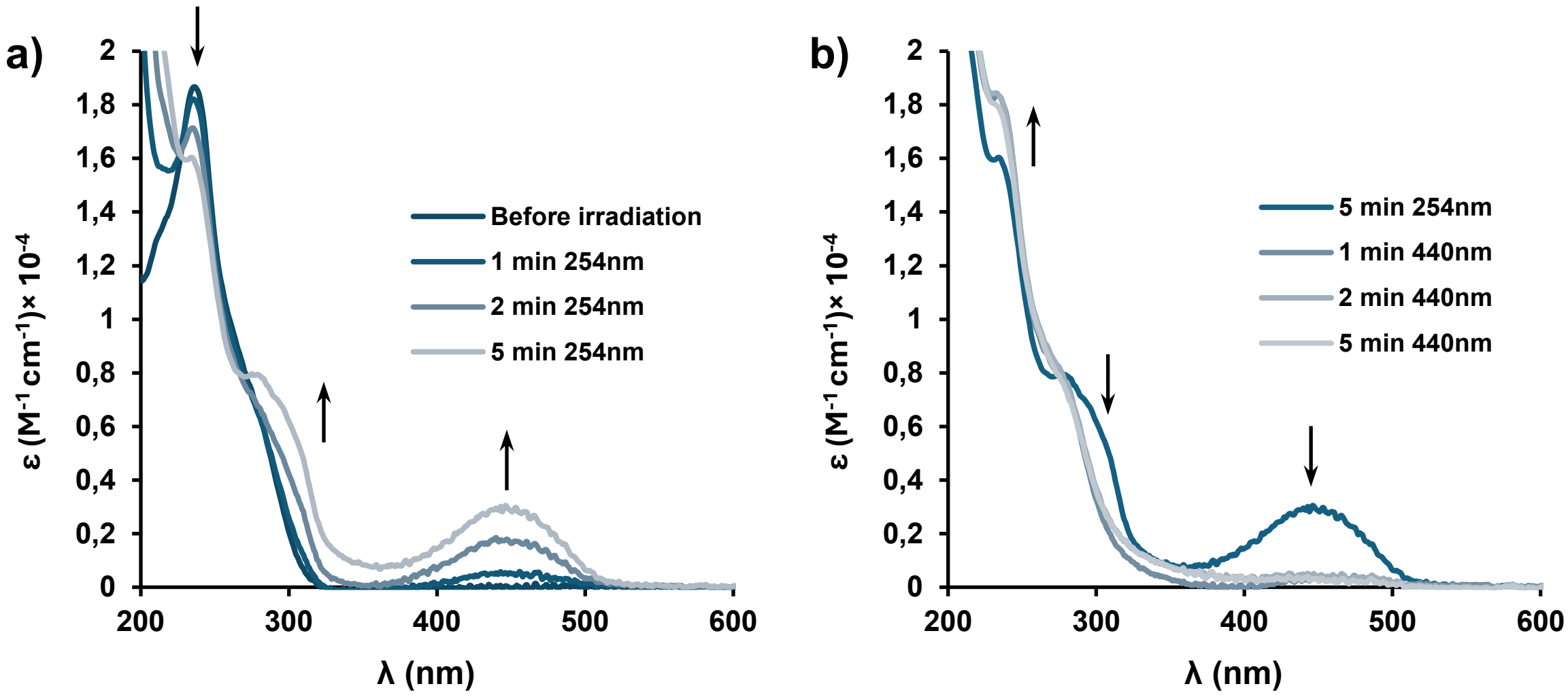

Fig. S7. Changes in the UV-vis spectra of **8** upon irradiation with **a)** UV (λ_{max} = 254 nm) and subsequently with **b)** visible (λ_{max} = 440 nm) light in solution (MeCN, c = 3 × 10^{-5} M).

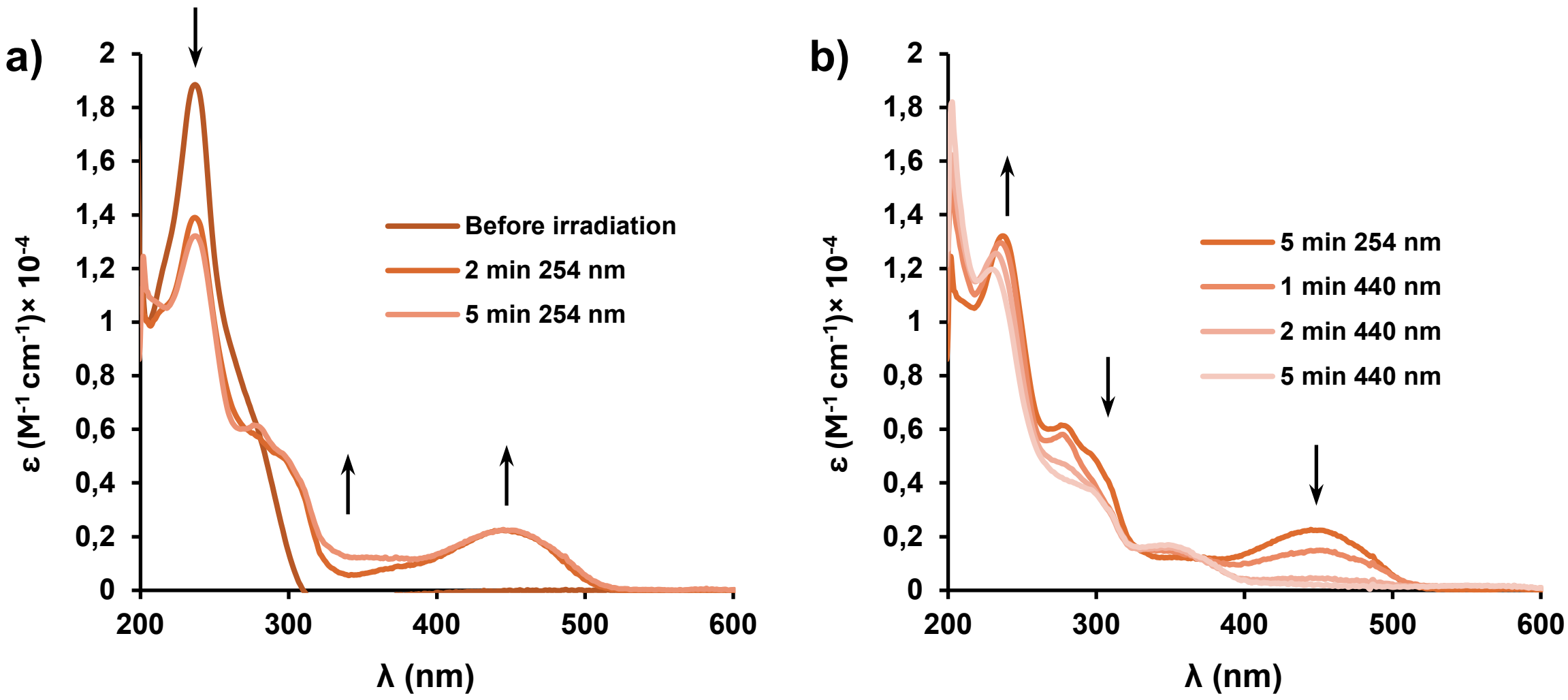

Fig. S8. Changes in the UV-vis spectra of **8** upon irradiation with **a)** UV (λ_{max} = 254 nm) and subsequently with **b)** visible (λ_{max} = 440 nm) light in solution (MeOH, c = 3 × 10^{-5} M).

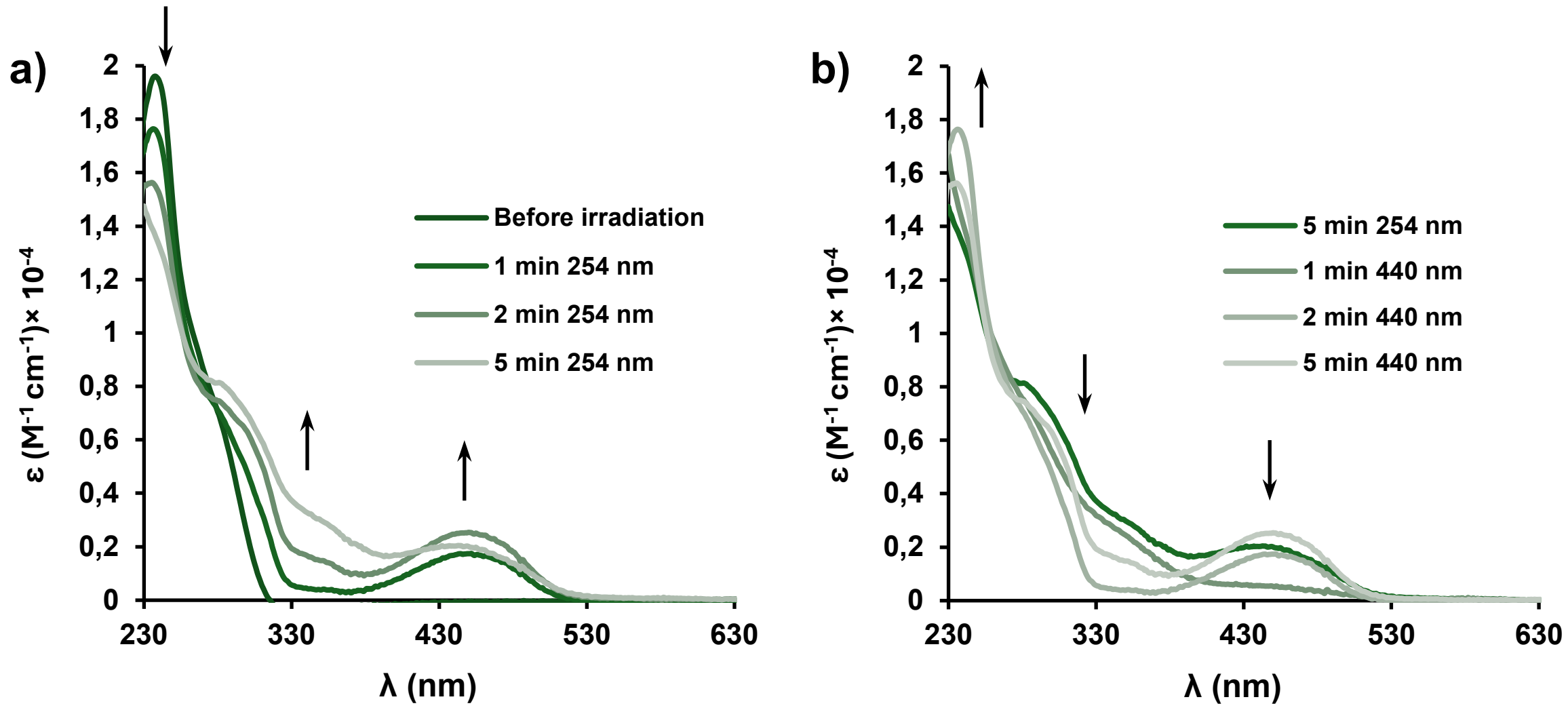

Fig. S9. Changes in the UV-vis spectra of **8** upon irradiation with **a)** UV (λ_{max} = 254 nm) and **b)** visible (λ_{max} = 440 nm) light in solution (DCM, c = 3 × 10^{-5} M).

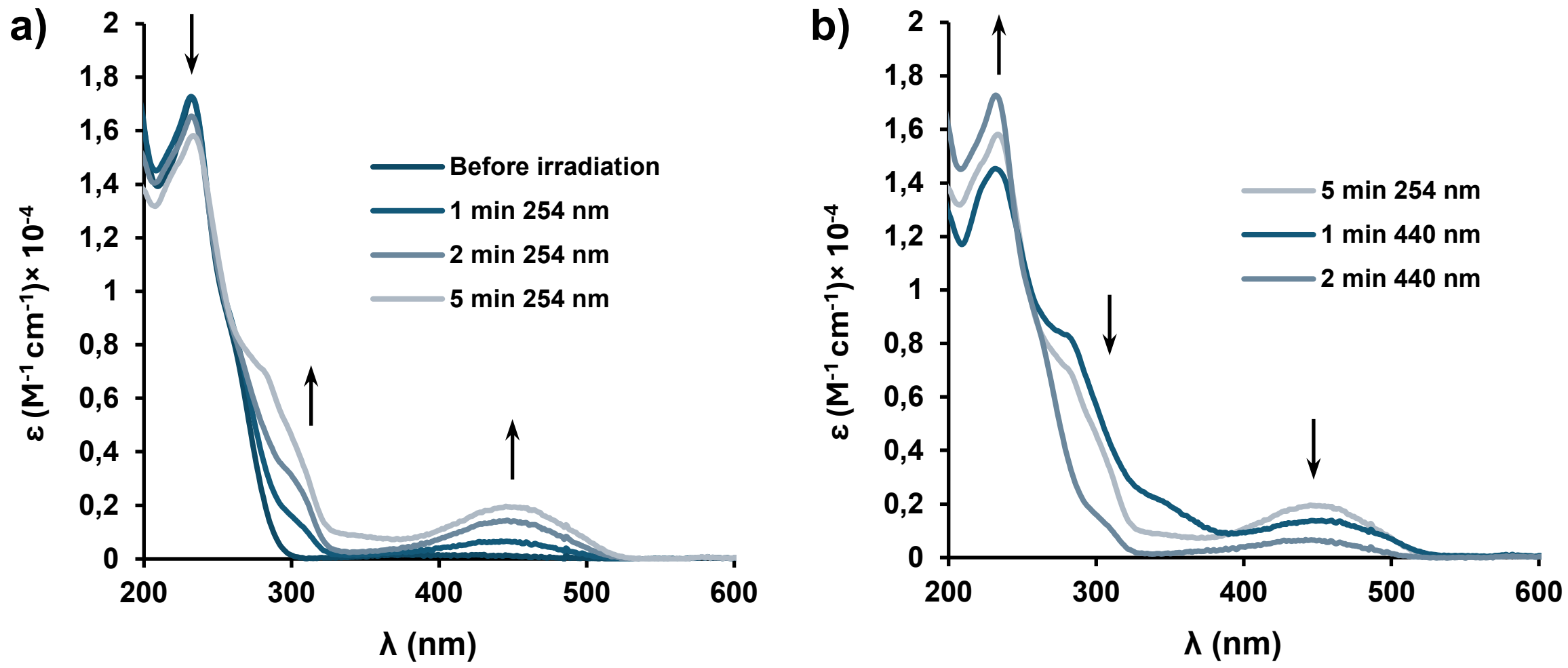

Fig. S10. Changes in the UV-vis spectra of **9** upon irradiation with **a)** UV (λ_{max} = 254 nm) and subsequently with **b)** visible (λ_{max} = 440 nm) light in solution (MeCN, c = 3 × 10^{-5} M).

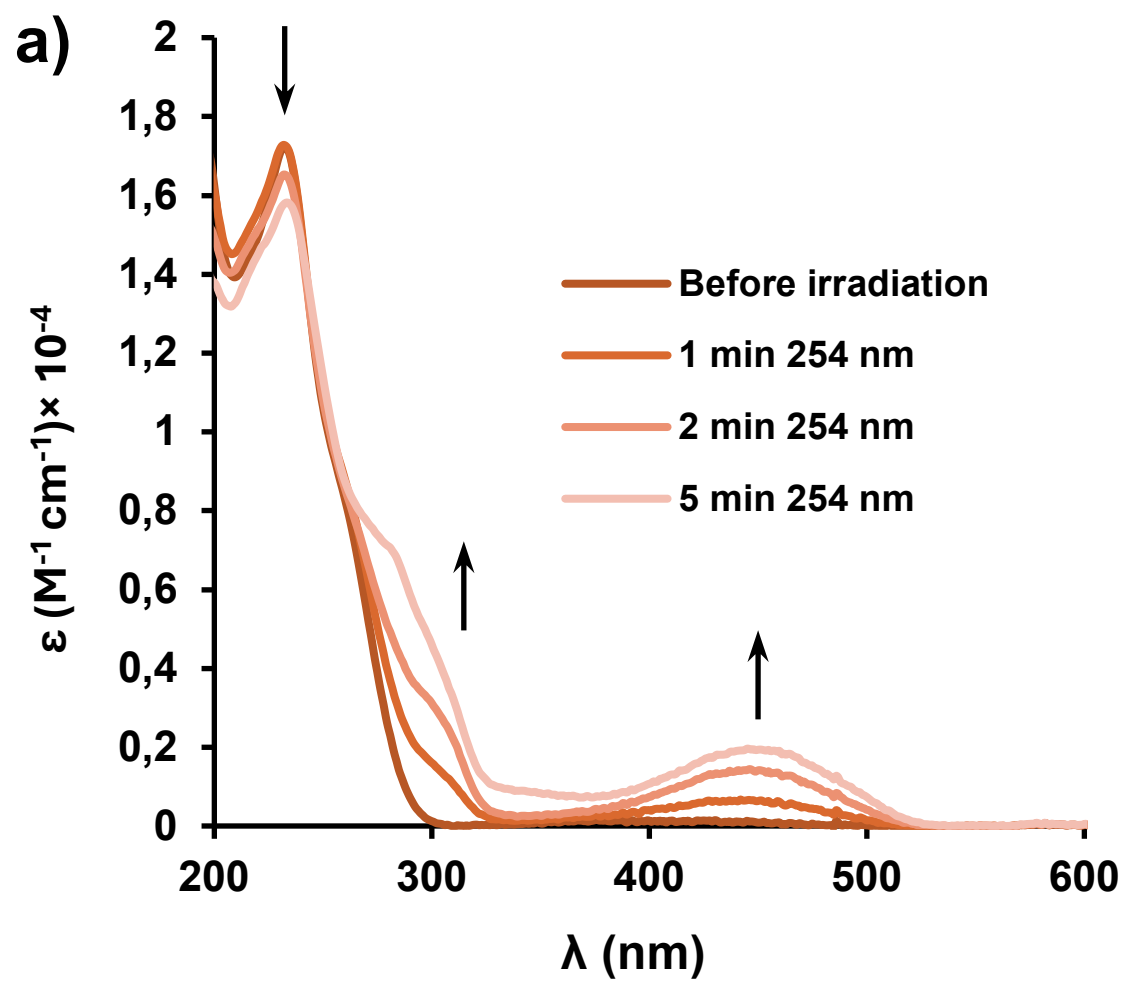

Fig. S11. Changes in the UV-vis spectra of **9** upon irradiation with **a)** UV (λ_{max} = 254 nm) and subsequently with **b)** visible (λ_{max} = 440 nm) light in solution (MeOH, c = 3 × 10^{-5} M).

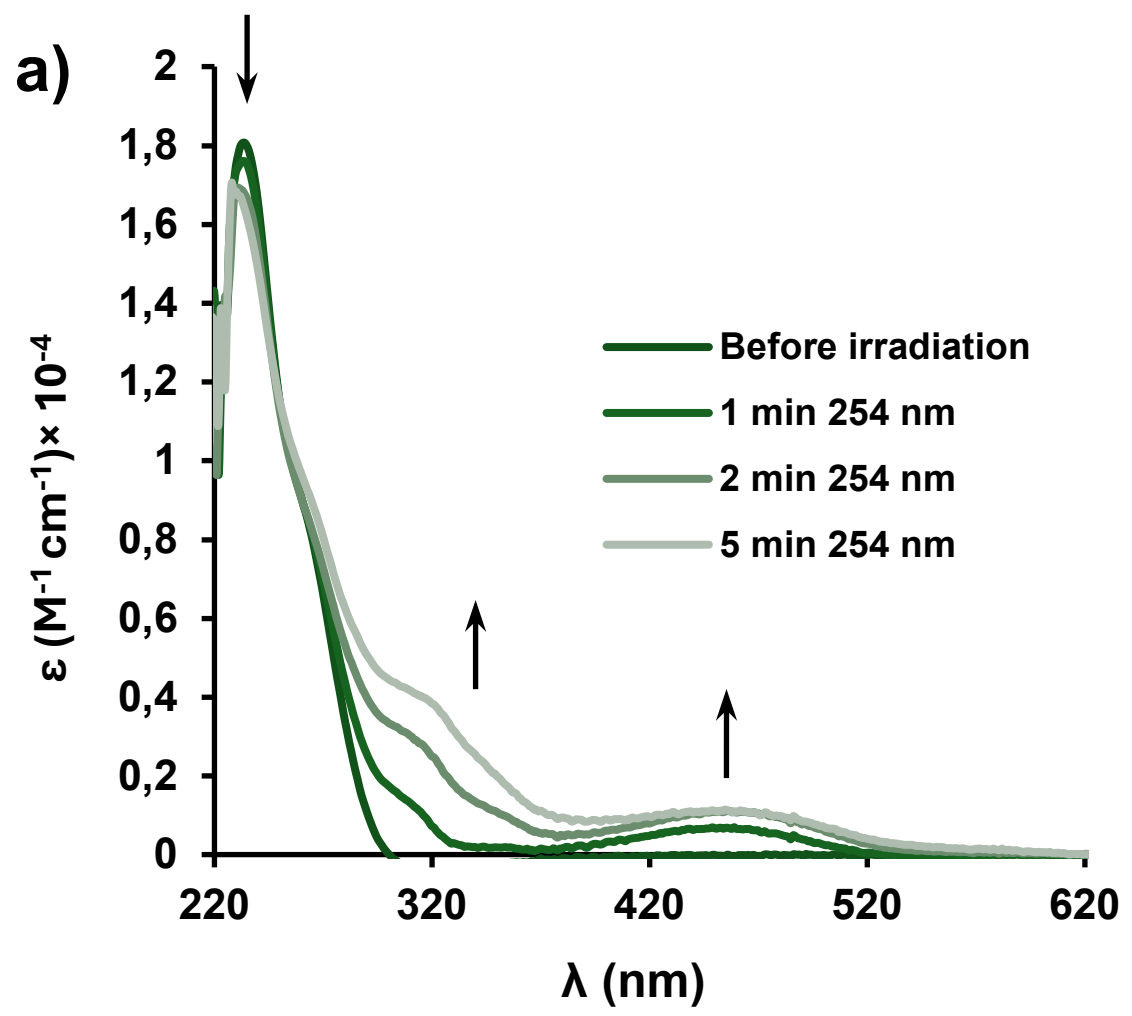

Fig. S12. Changes in the UV-vis spectra of **9** upon irradiation with **a)** UV (λ_{max} = 254 nm) and subsequently with **b)** visible (λ_{max} = 440 nm) light in solution (DCM, c = 3 × 10^{-5} M).

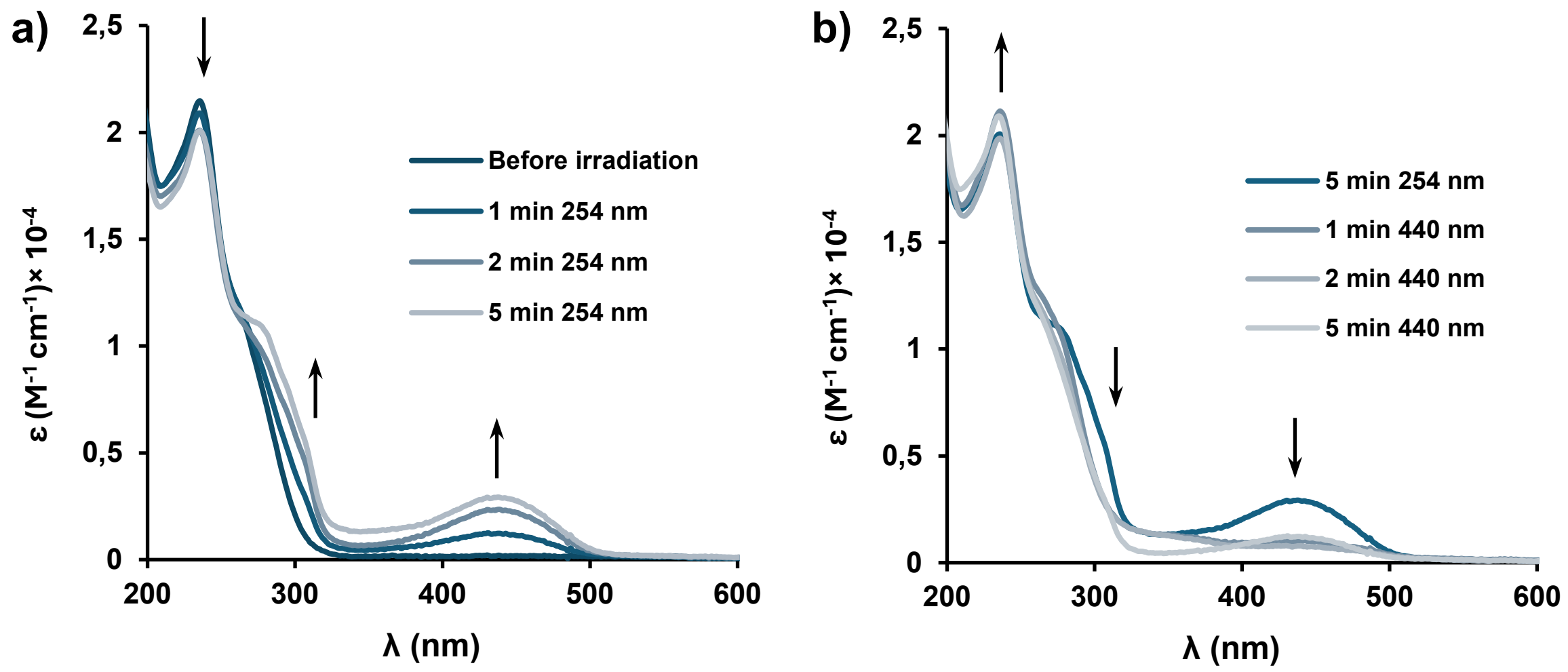

Fig. S13. Changes in the UV-vis spectra of **10** upon irradiation with **a)** UV (λ_{max} = 254 nm) and subsequently with **b)** visible (λ_{max} = 440 nm) light in solution (MeCN, c = 3 × 10^{-5} M).

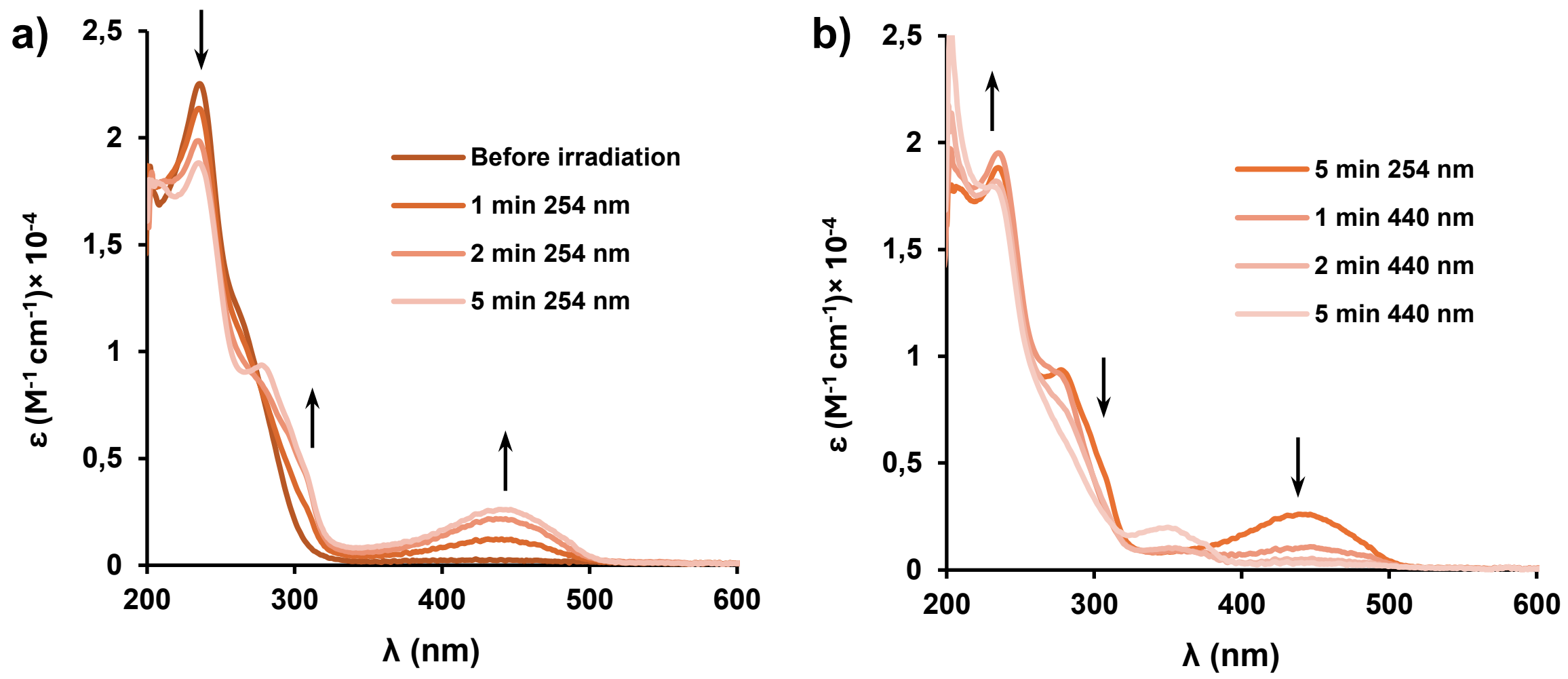

Fig. S14. Changes in the UV-vis spectra of **10** upon irradiation with **a)** UV (λ_{max} = 254 nm) and subsequently with **b)** visible (λ_{max} = 440 nm) light in solution (MeOH, c = 3 × 10^{-5} M).

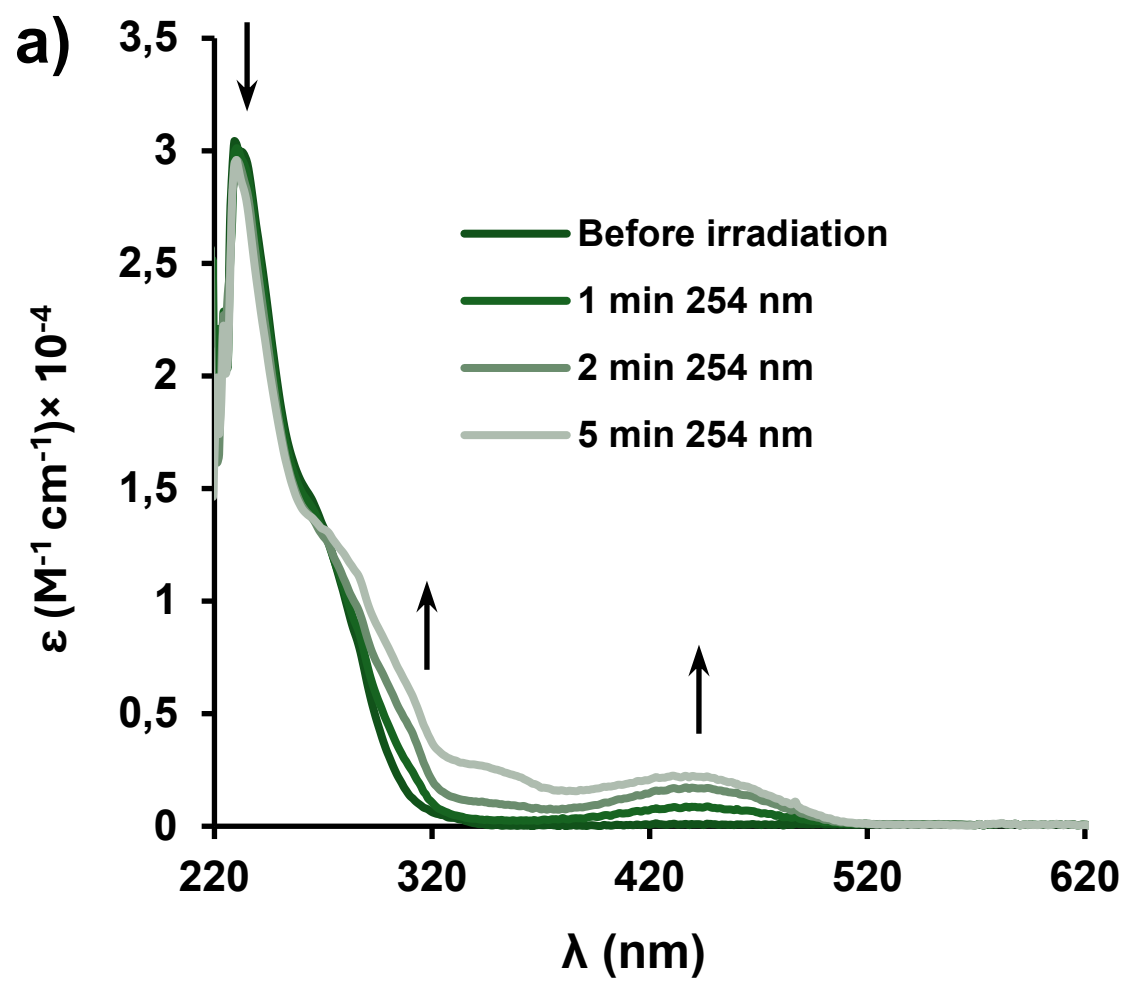

Fig. S15. Changes in the UV-vis spectra of **10** upon irradiation with **a)** UV (λ_{max} = 254 nm) and subsequently with **b)** visible (λ_{max} = 440 nm) light in solution (DCM, c = 3 × 10^{-5} M).

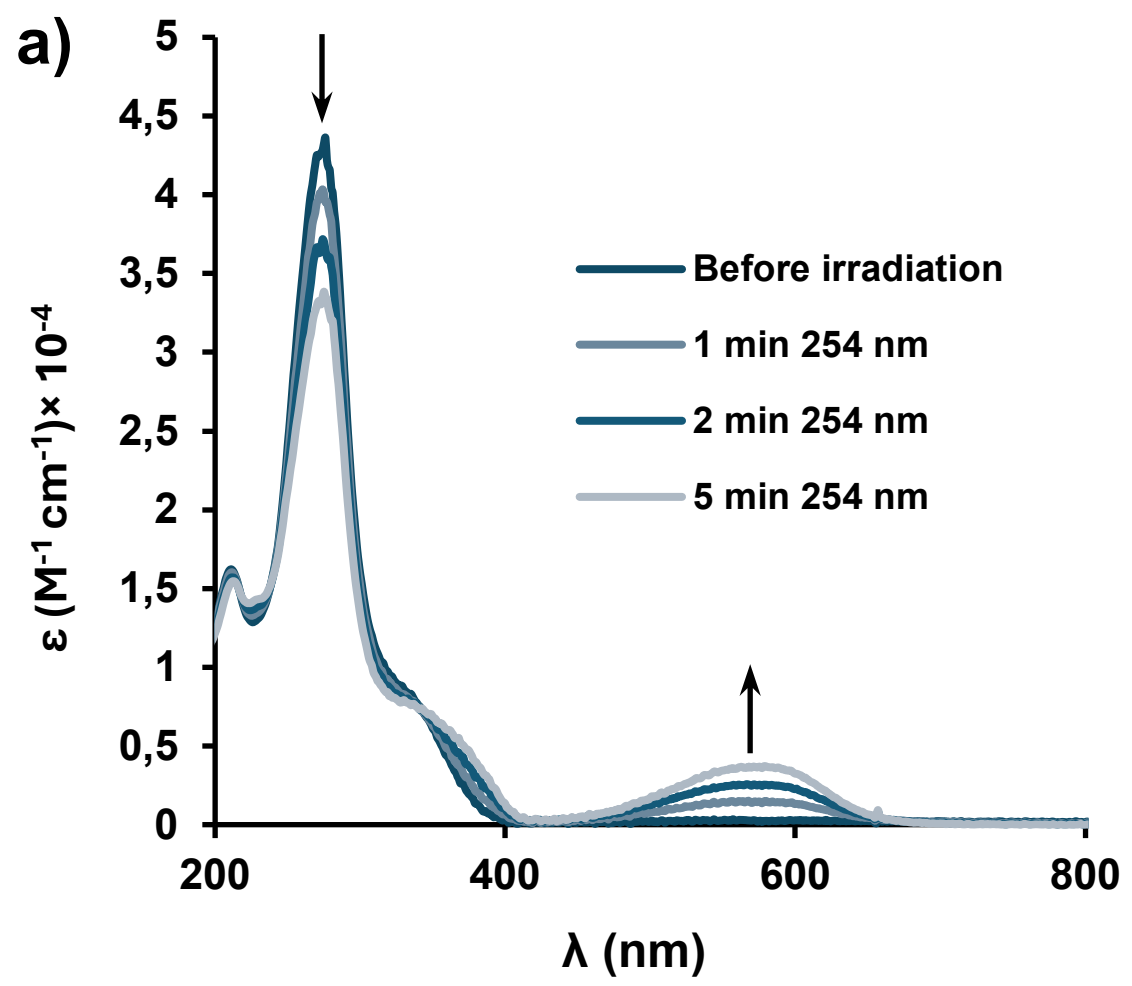

Fig. S16. Changes in the UV-vis spectra of **11** upon irradiation with **a)** UV (λ_{max} = 254 nm) and subsequently with **b)** visible (λ_{max} = 585 nm) light in solution (MeCN, c = 3 × 10^{-5} M).

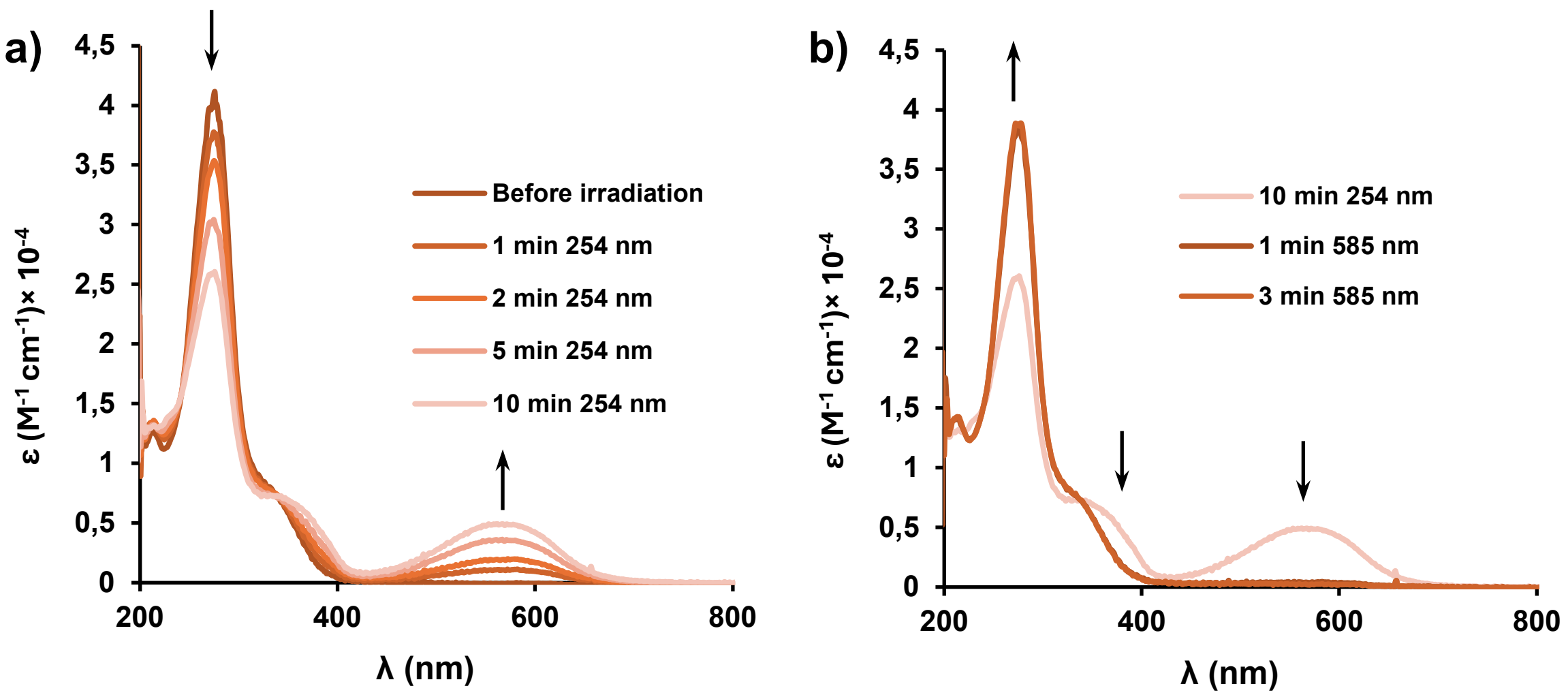

Fig. S17. Changes in the UV-vis spectra of **11** upon irradiation with **a)** UV (λ_{max} = 254 nm) and subsequently with **b)** visible (λ_{max} = 585 nm) light in solution (MeOH, c = 3 × 10^{-5} M).

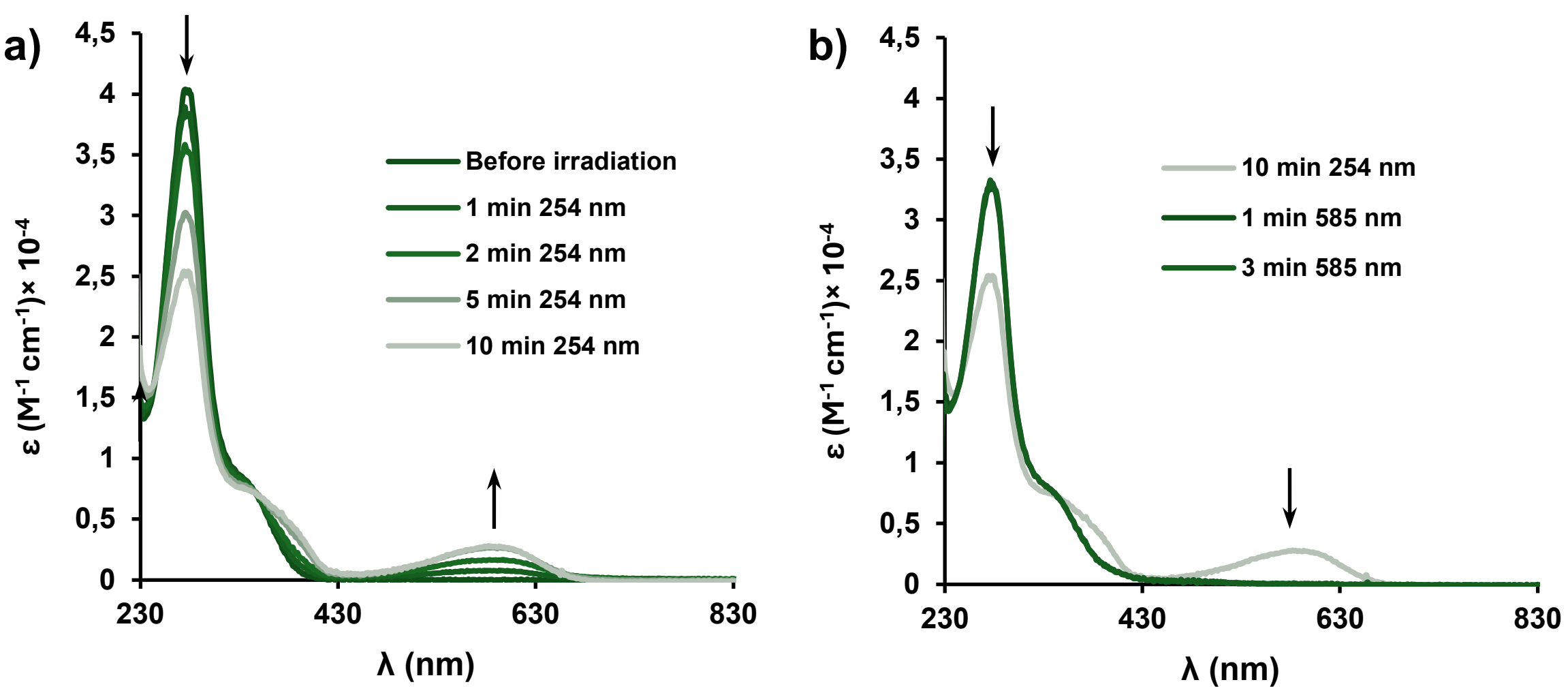

Fig. S18. Changes in the UV-vis spectra of **11** upon irradiation with **a)** UV (λ_{max} = 254 nm) and subsequently with **b)** visible (λ_{max} = 585 nm) light in solution (DCM, c = 3 × 10^{-5} M).

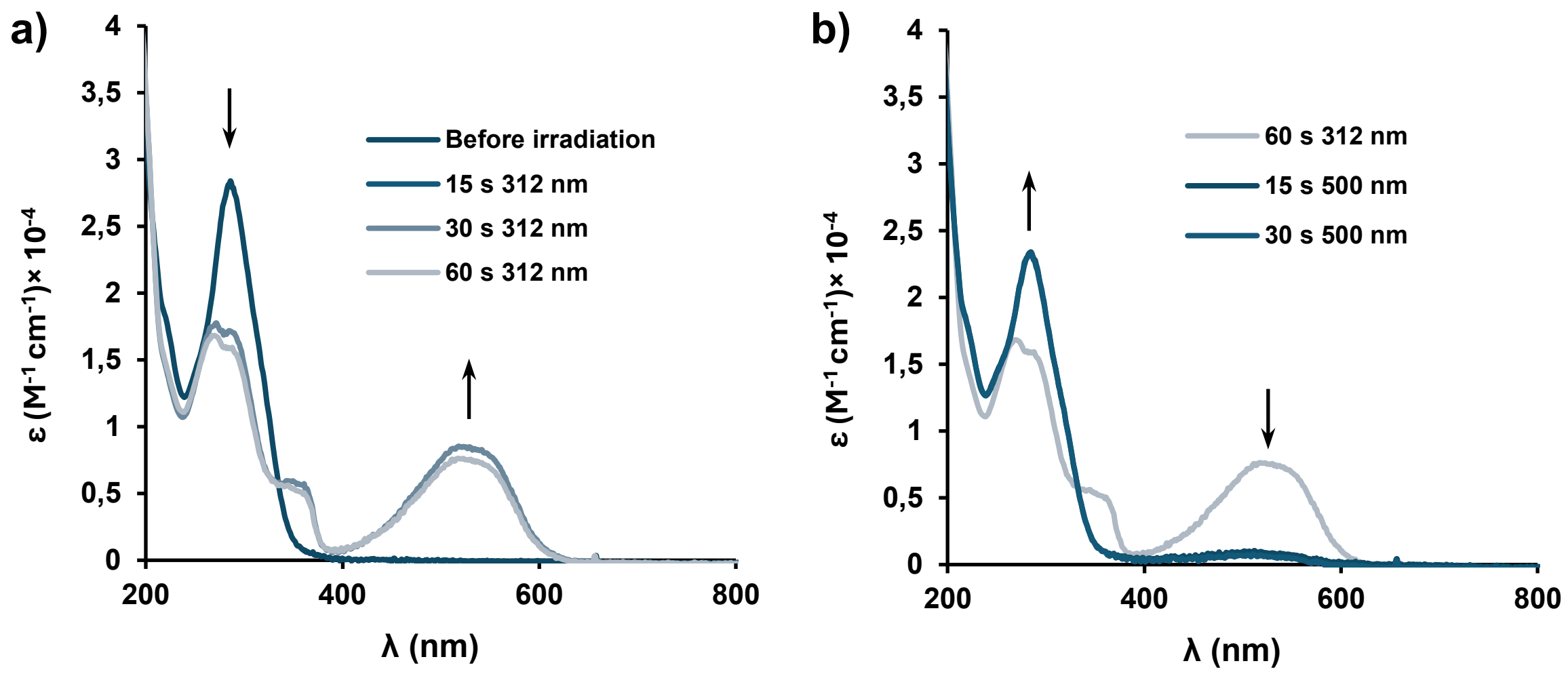

Fig. S19. Changes in the UV-vis spectra of **13** upon irradiation with **a)** UV (λ_{max} = 312 nm) and subsequently with **b)** visible (λ_{max} = 500 nm) light in solution (MeCN, c = 3 × 10^{-5} M).

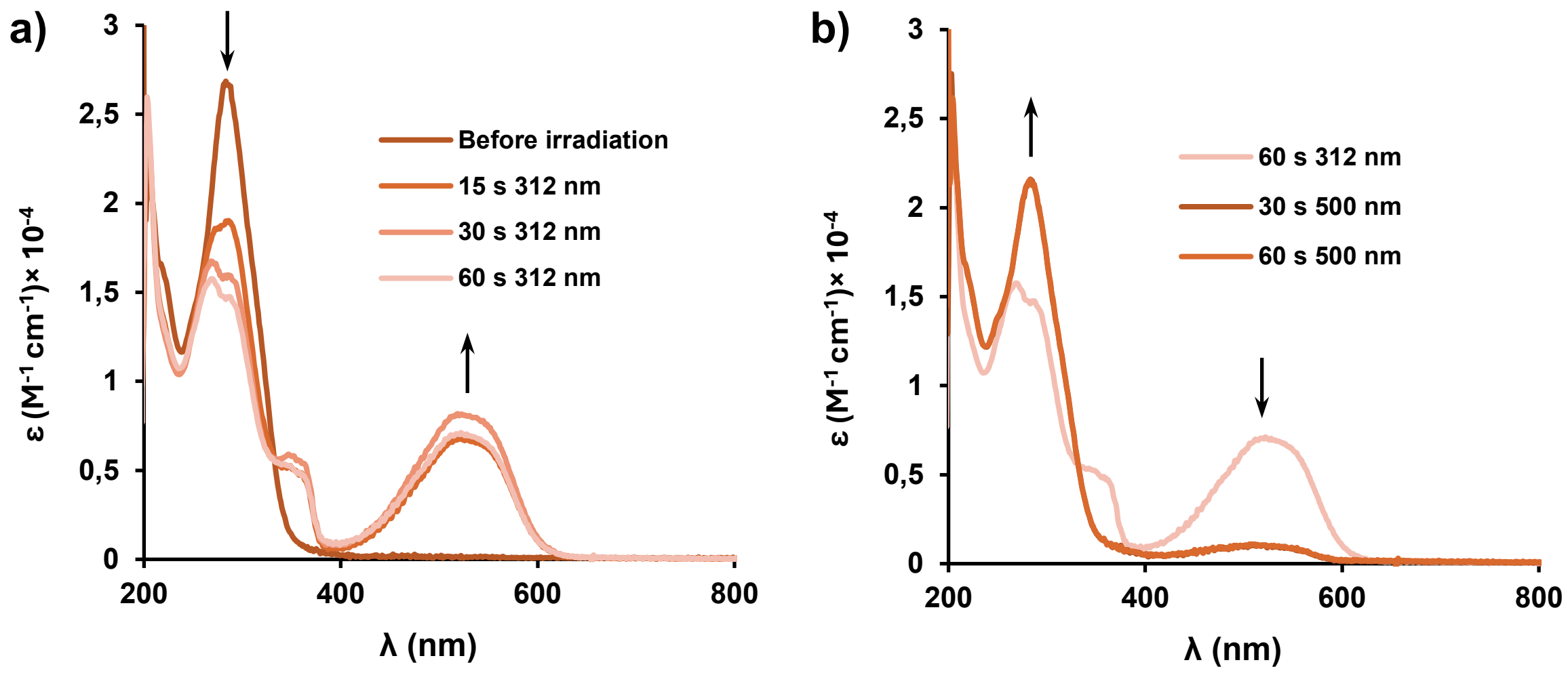

Fig. S20. Changes in the UV-vis spectra of **13** upon irradiation with **a)** UV (λ_{max} = 312 nm) and subsequently with **b)** visible (λ_{max} = 500 nm) light in solution (MeOH, c = 3 × 10^{-5} M).

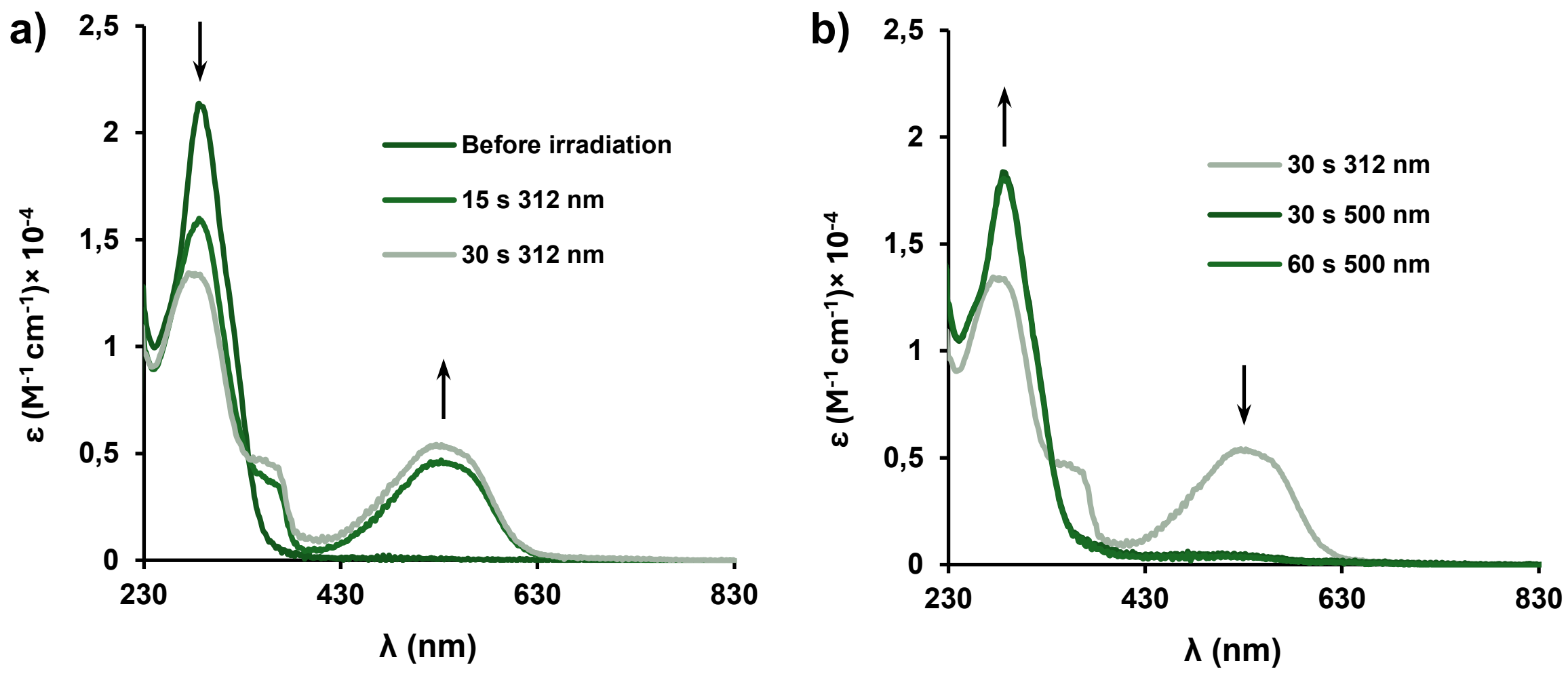

Fig. S21. Changes in the UV-vis spectra of **13** upon irradiation with **a)** UV (λ_{max} = 312 nm) and subsequently with **b)** visible (λ_{max} = 500 nm) light in solution (DCM, c = 3 × 10^{-5} M).

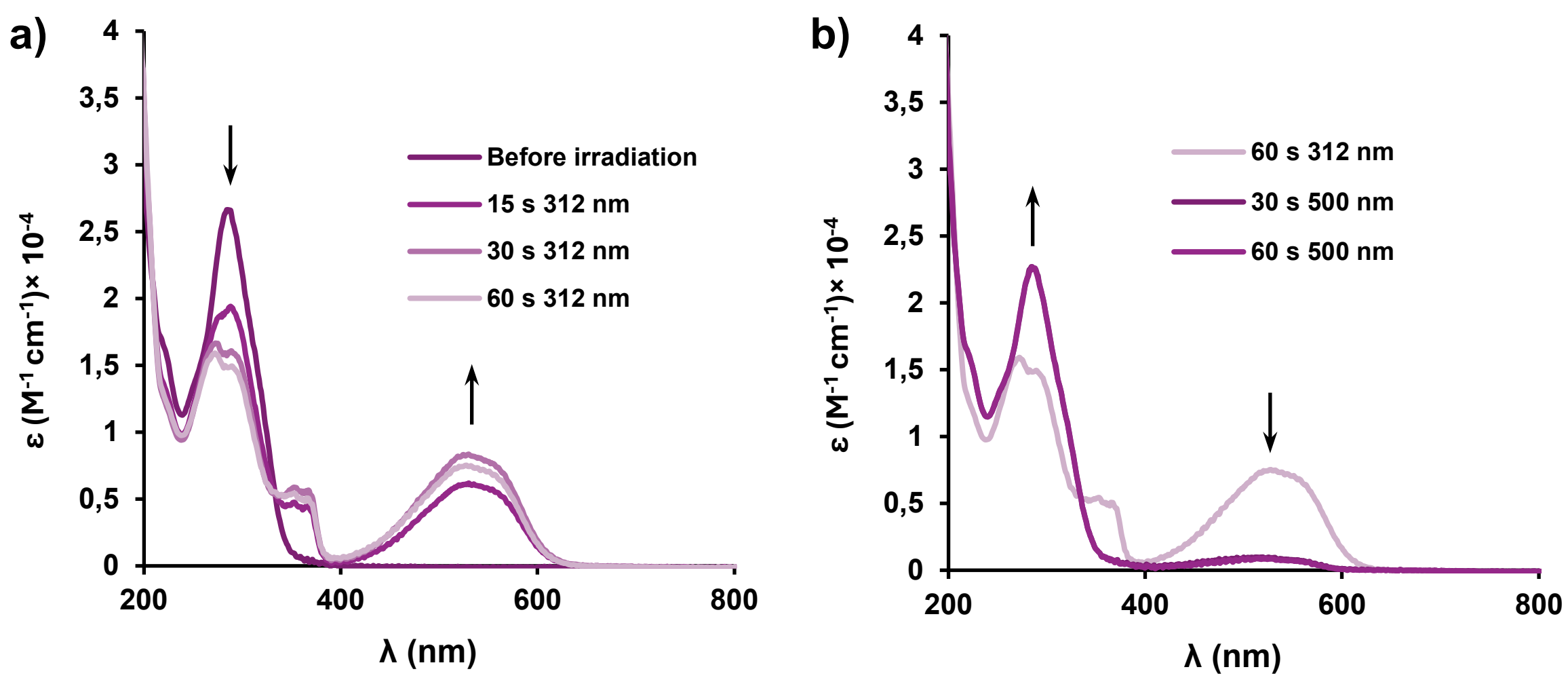

Fig. S22. Changes in the UV-vis spectra of **13** upon irradiation with **a)** UV (λ_{max} = 312 nm) and subsequently with **b)** visible (λ_{max} = 500 nm) light in solution (hexane, c = 3 × 10^{-5} M).

S4 Switching cycles – Analysis of fatigue resistance

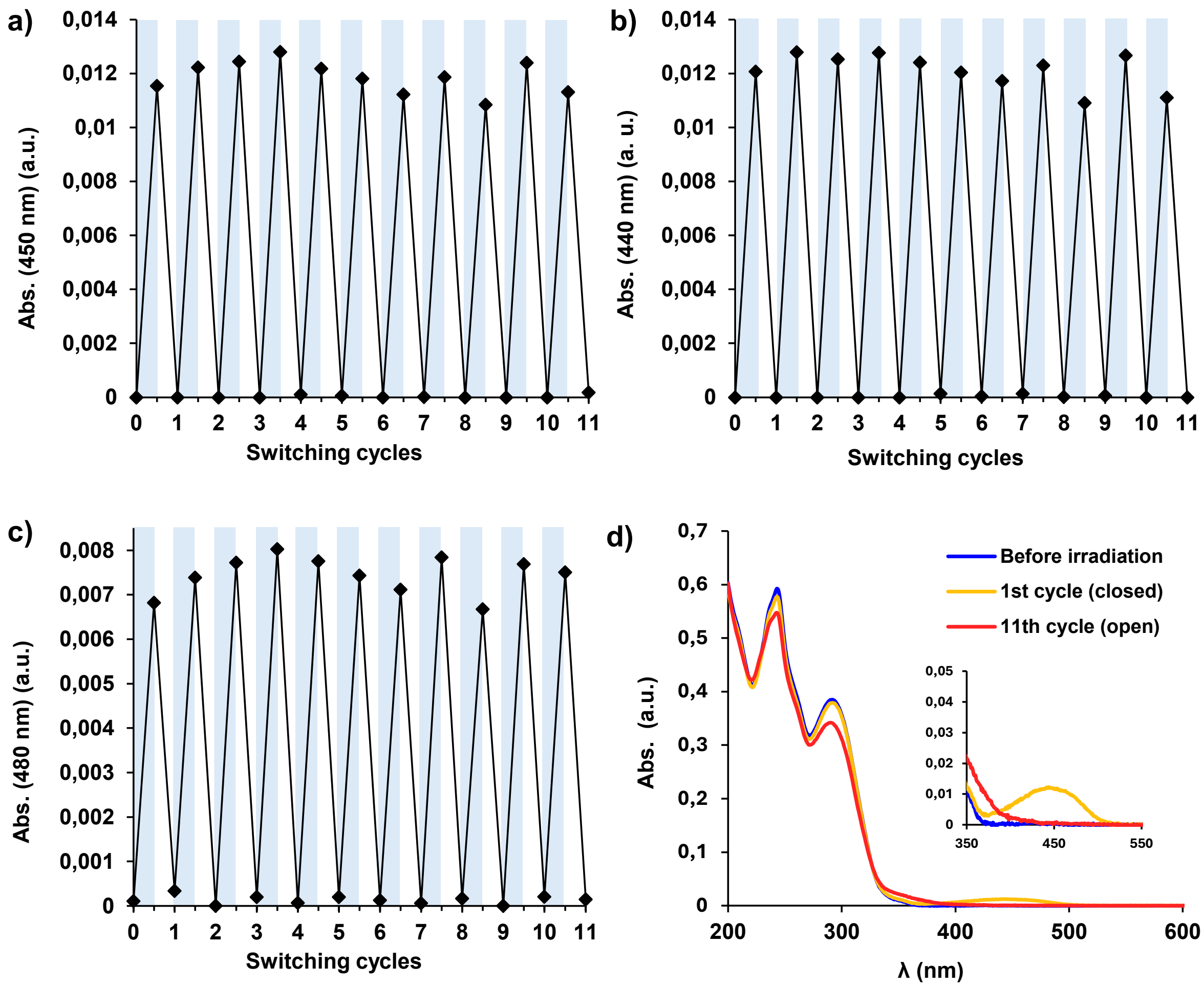

Fig. S23. Absorbance of compound **7** (3×10^{-5} M in MeCN) in the visible spectrum: **a)** @450 nm, **b)** @440 nm, **c)** @480 nm – upon alternating irradiation with UV (254 nm, 60 s) and visible (440 nm, 60s) light sources. **d)** UV-vis spectra of compound **7** at different stages of the photoswitching cycles.

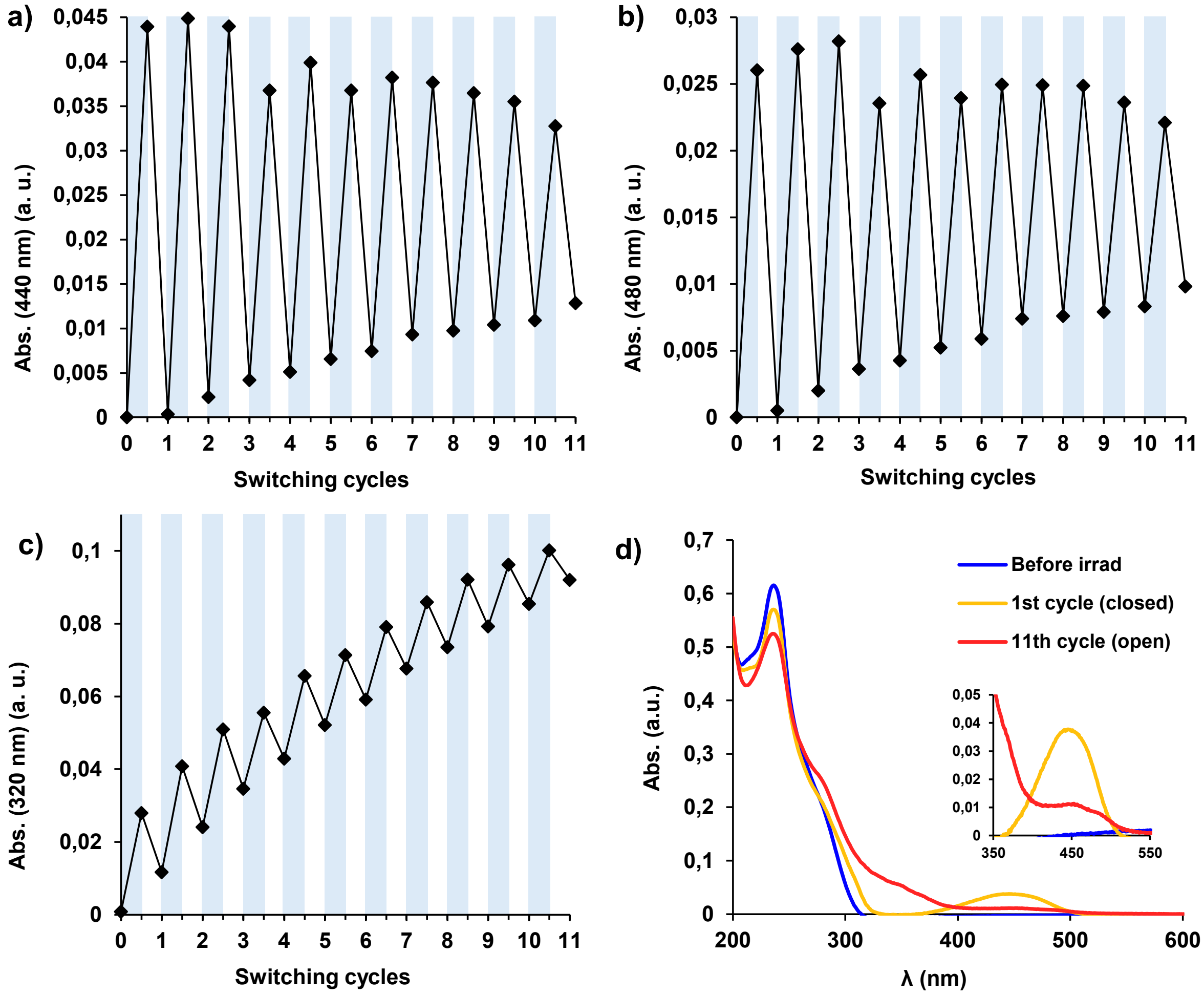

Fig. S24. Absorbance of compound **8** (3×10^{-5} M in MeCN) in the visible spectrum: **a)** @440 nm, **b)** @480 nm, **c)** @320 nm – upon alternating irradiation with UV (254 nm, 60 s) and visible (440 nm, 60s) light sources. **d)** UV-vis spectra of compound **8** at different stages of the photoswitching cycles. The baseline shift observed on **Fig. S22. c)** is due to the overlapping absorption band of the irreversible side product.

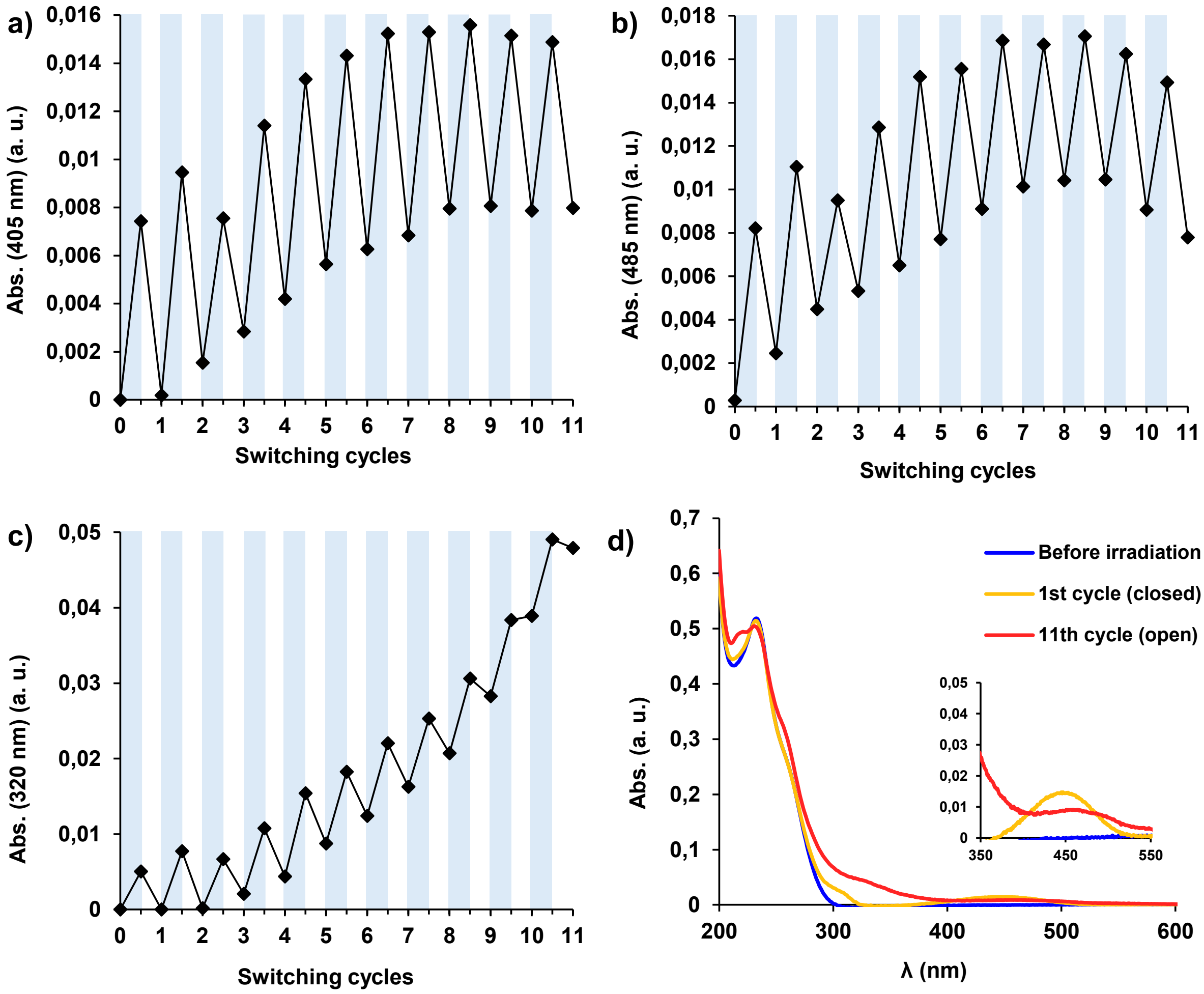

Fig. S25. Absorbance of compound **9** (3×10^{-5} M in MeCN) in the visible spectrum: **a)** @405 nm, **b)** @485 nm, **c)** @320 nm – upon alternating irradiation with UV (254 nm, 60 s) and visible (440 nm, 60s) light sources. **d)** UV-vis spectra of compound **9** at different stages of the photoswitching cycles. The baseline shift observed on **Fig. S23. a-c)** is due to the overlapping absorption band of the irreversible side product.

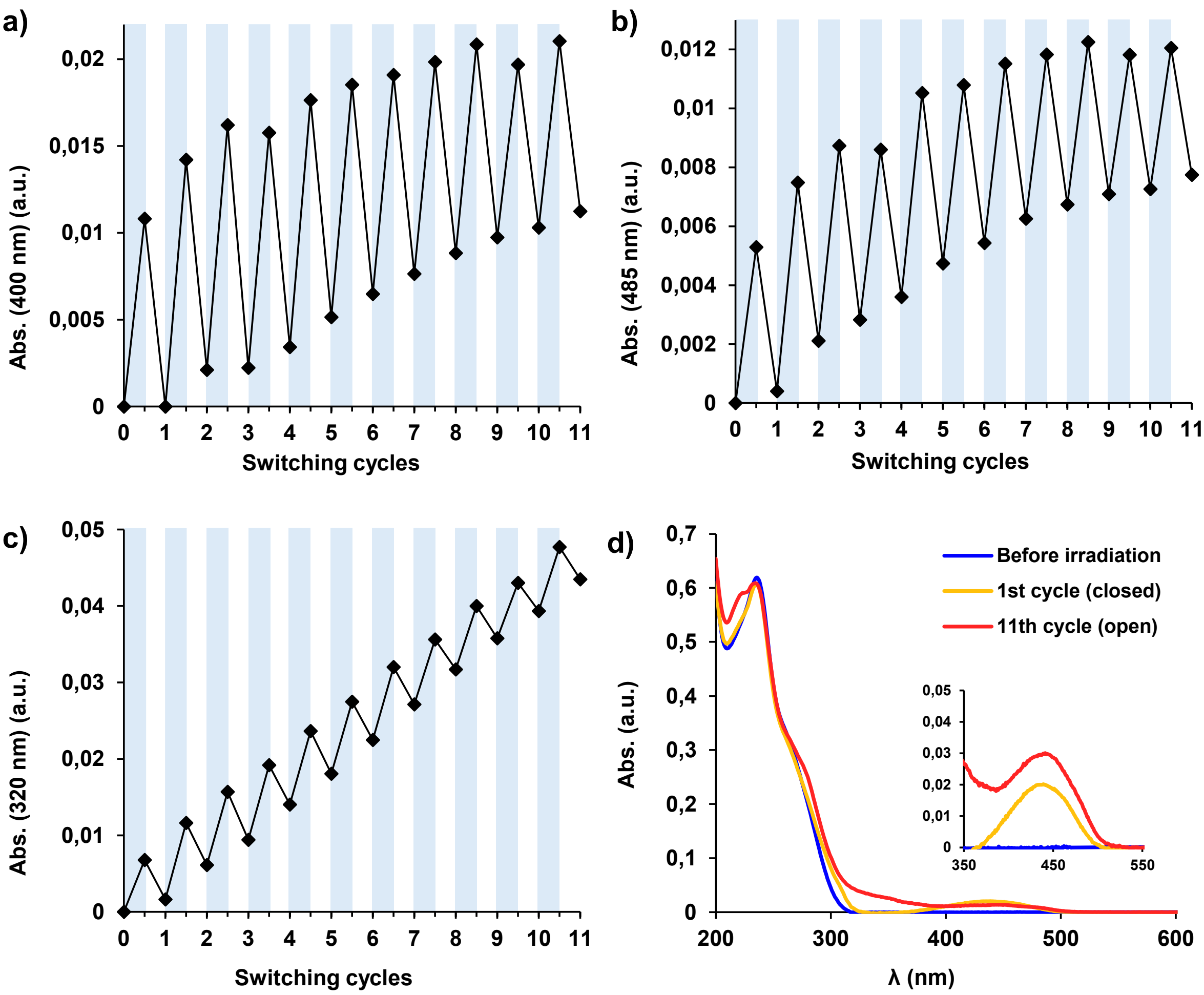

Fig. S26. Absorbance of compound **10** (3×10^{-5} M in MeCN) in the visible spectrum: **a)** @400 nm, **b)** @485 nm, **c)** @320 nm – upon alternating irradiation with UV (254 nm, 60 s) and visible (440 nm, 60s) light sources. **d)** UV-vis spectra of compound **10** at different stages of the photoswitching cycles. The baseline shift observed on **Fig. S24. a-c)** is due to the overlapping absorption band of the irreversible side product.

S5 Kinetic studies on the thermal ring opening reaction

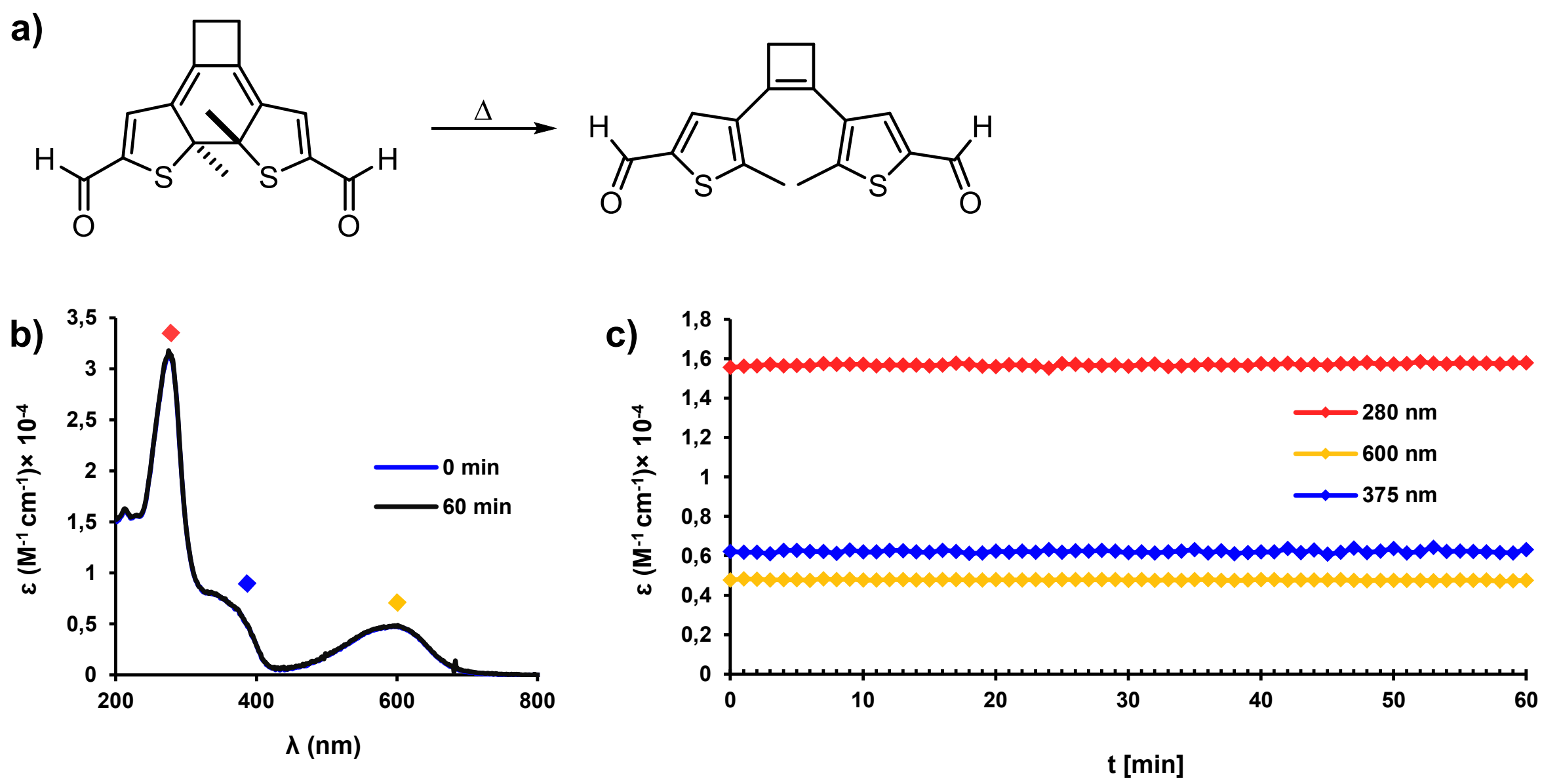

Fig. S27. a) Thermal cycloreversion of compound **11.** b) UV-vis spectra of **11** before and after 60 min relaxation time (MeCN, T = 25.8°C, C = 3×10^{-5} M). c) Monitoring of molar absorption coefficient values at 280, 375 and 600 nm throughput the experiment.

Experimental procedure: A sample of **11** (MeCN, c = 3×10^{-5} M) was prepared in a quartz cuvette and was irradiated at 254 nm for 10 minutes to reach PSS. Subsequently, it was placed into the UV-vis spectrophotometer sample holder and the pre-set experimental method (60 minutes, spectrum acquisition frequency 60s, room temperature) was started.

The results clearly illustrate the lack of thermal cycloreversion under these conditions.

S6 ^{1}H NMR spectroscopic characterization of the photochemical transformation of compound 11

Experimental procedure and data interpretation: A ~2.0 mg sample of **11** was dissolved in deuterated benzene/acetonitrile (C_6D_6/CD_3CN, 600-700 μL) and the solution was transferred to a 5 mm quartz NMR tube sealed with a plastic cap. This sample was subjected to ^{1}H NMR measurement after lock, tune and shimming of the instrument. Subsequently, the sample was removed and externally irradiated at 254/585 nm for the depicted intervals. After each irradiation period, the NMR was retuned and reshimmed before data acquisition. Notably, all samples were irradiated under air without signs of significant decomposition.

Compound **11** was first irradiated at 254 nm in both solvents. After 90 minutes a new set of signals appeared in the spectra, however exhibiting very low intensities in both cases. In contrast to this we observed the solutions in the NMR tubes turning from pale-yellow to deep violet. Accordingly, we extended the irradiation time up to 16 hours, which resulted in higher intensity signals. Notably, we not only detected the characteristic shift of thienyl methyl groups but the aldehyde protons as well. By integrating the representative peak areas, we could quantify the relative distribution of open/closed forms after irradiation. After overnight irradiation at 254 nm the samples were exposed to 585 nm visible light for 60 minutes, which resulted in complete cycloreversion based on the disappearance of the signals corresponding to the closed isomer of **11**. *These results indicate the reversible photoswitching of **11**.*

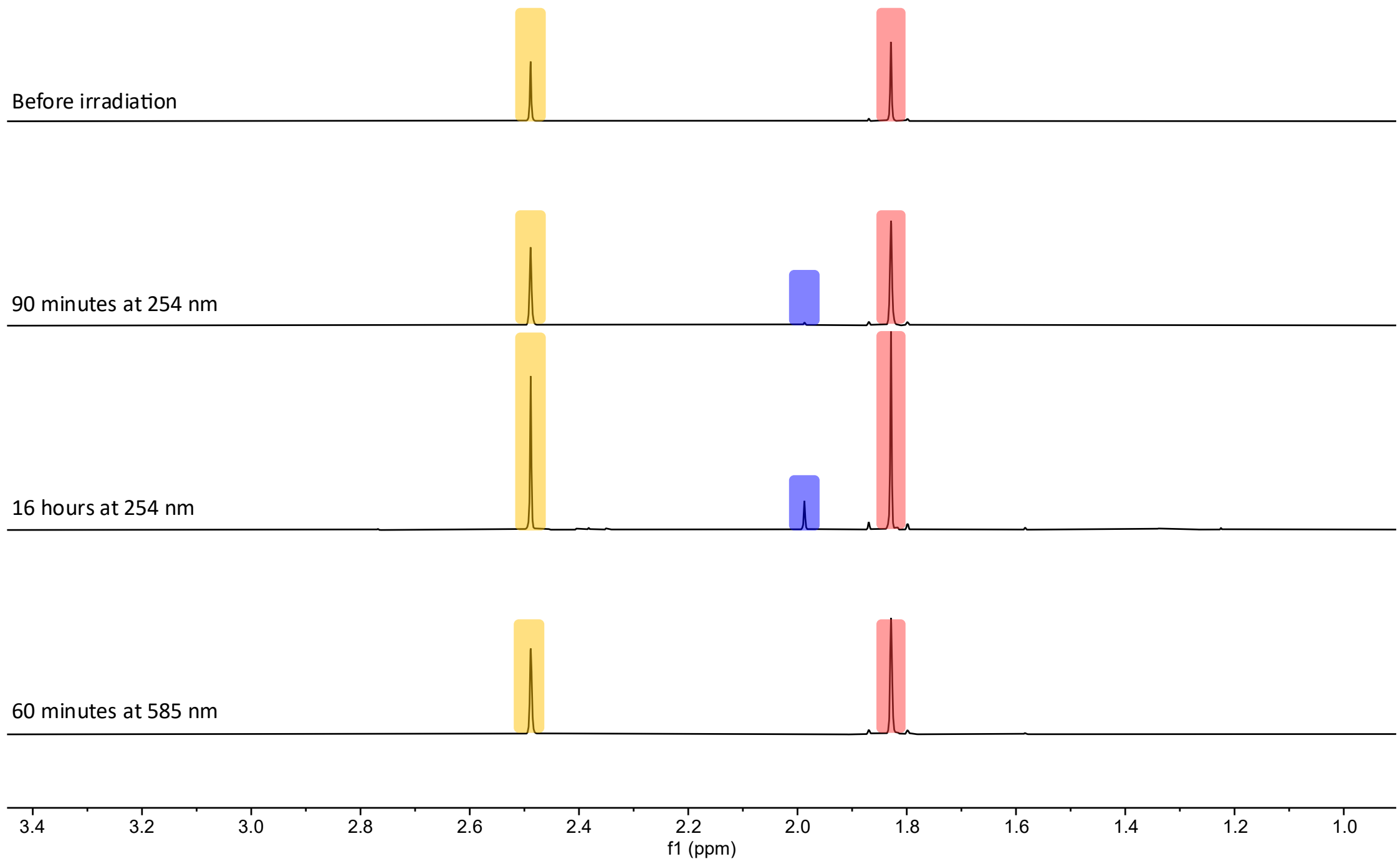

Fig. S28. ^{1}H NMR spectra of **11** in C_6D_6 after irradiation at 254 nm (aliphatic region, ppm scale).

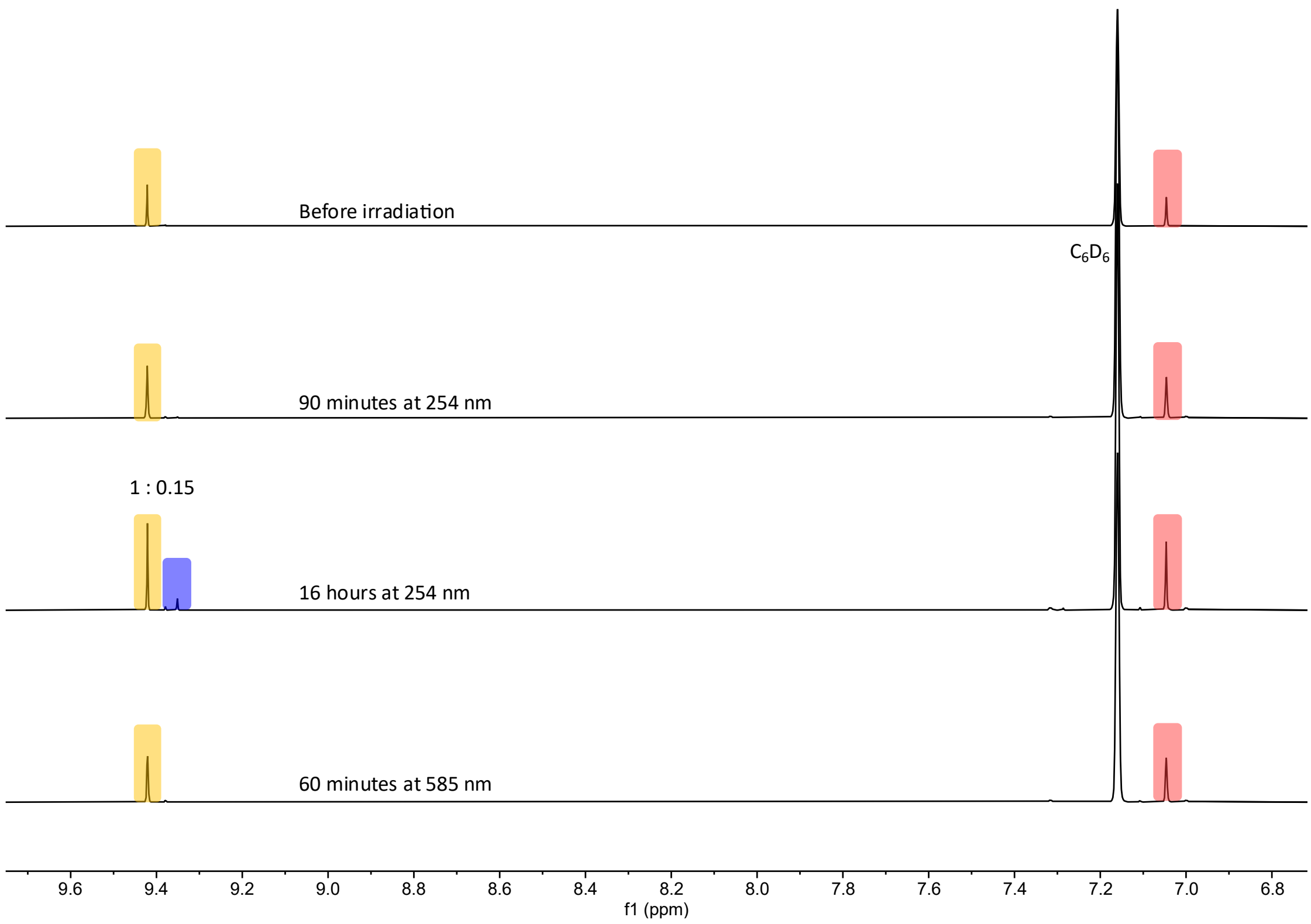

Fig. S29. ^{1}H NMR spectra of **11** in C_6D_6 after irradiation at 254 nm (aromatic region, ppm scale).

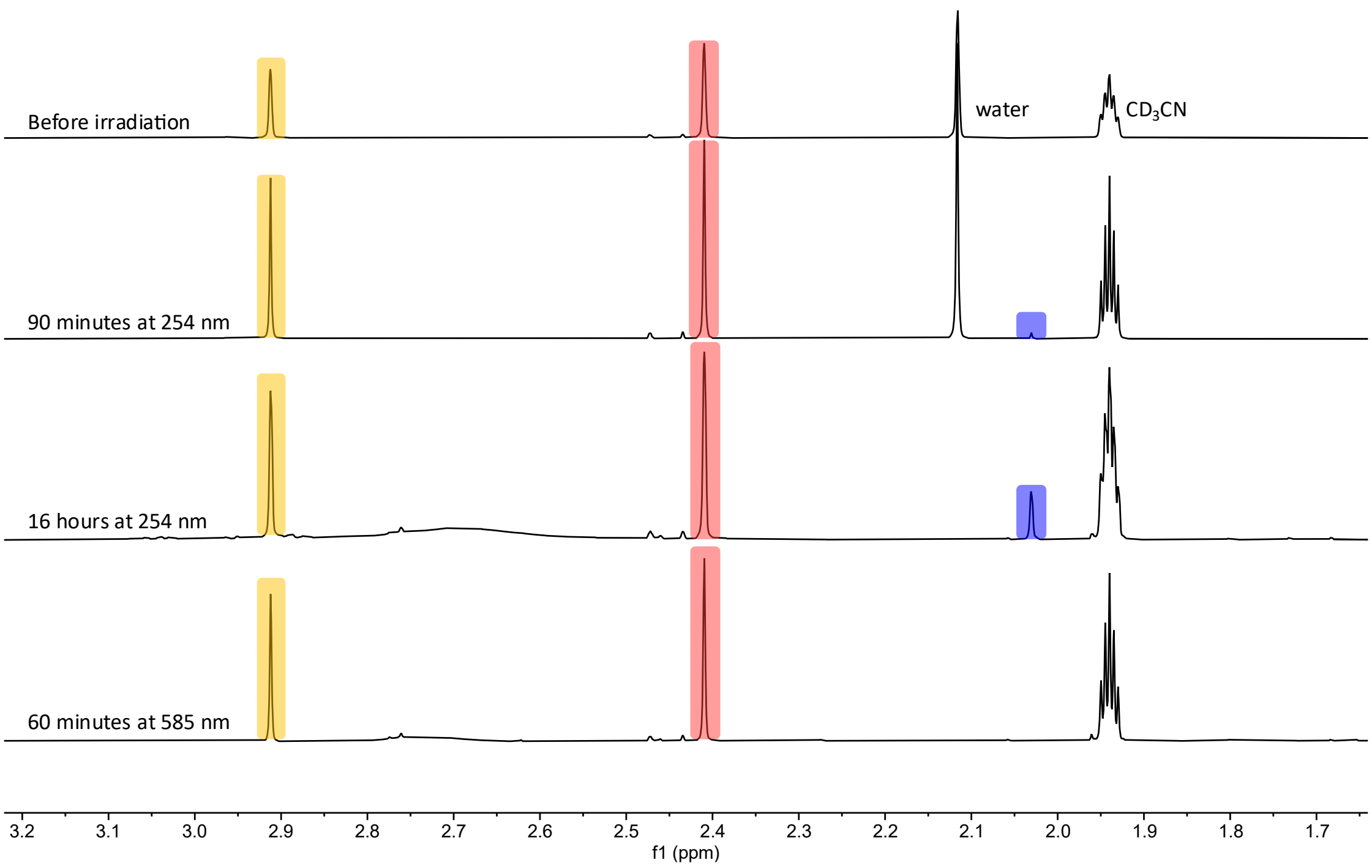

Fig. S30. ^{1}H NMR spectra of **11** in CD_3CN after irradiation at 254 nm (aliphatic region, ppm scale).

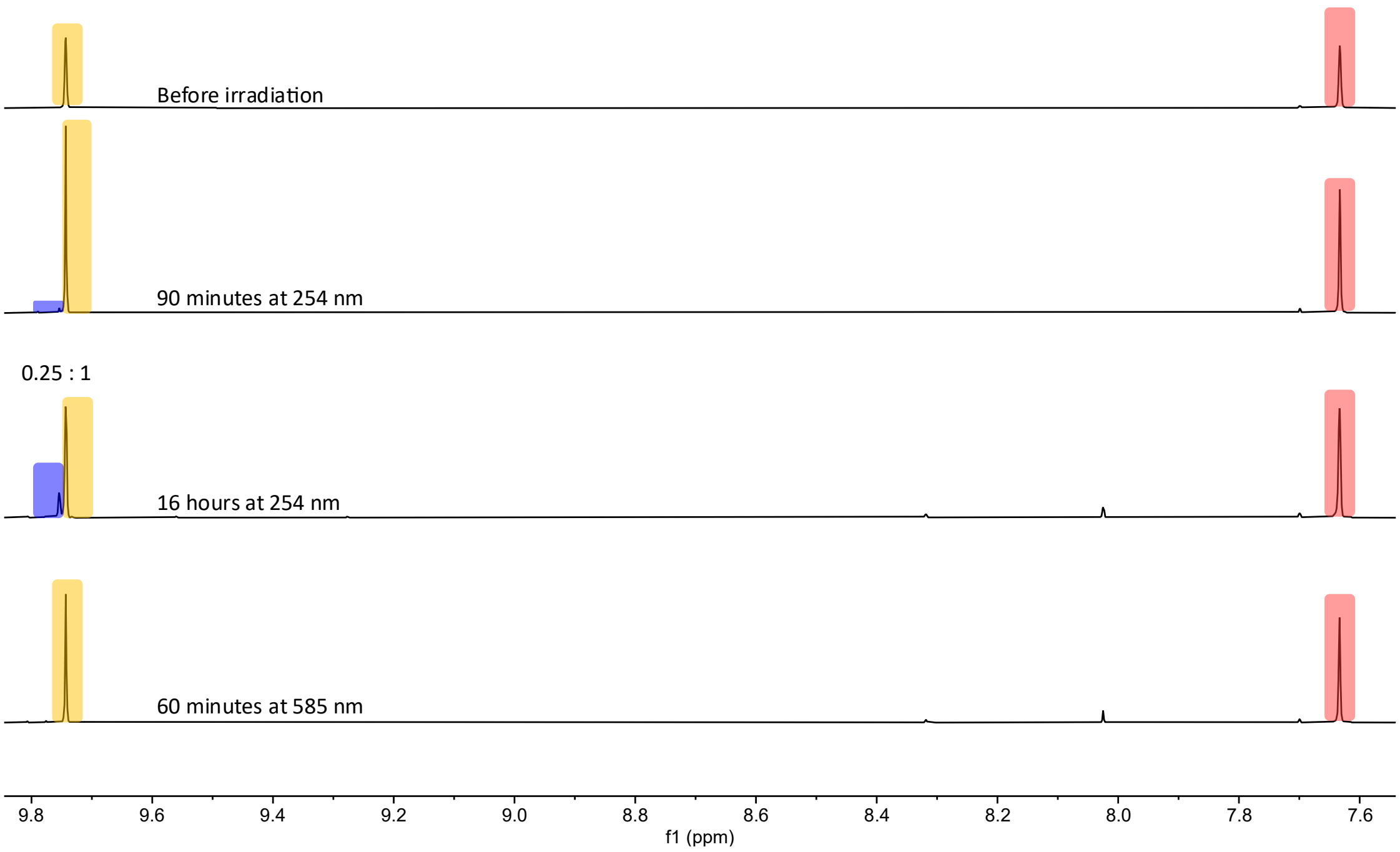

Fig. S31. ^{1}H NMR spectra of **11** in CD_3CN after irradiation at 254 nm (aromatic region, ppm scale).

S7 Photoswitching in solid phase

Upon irradiation with UV light (254 nm) pale-yellow crystals of **8** turned bright red. The photoswitching was shown to be reversible in the solid phase, as the crystals returned to the original yellow color after irradiation with visible light (440 nm).

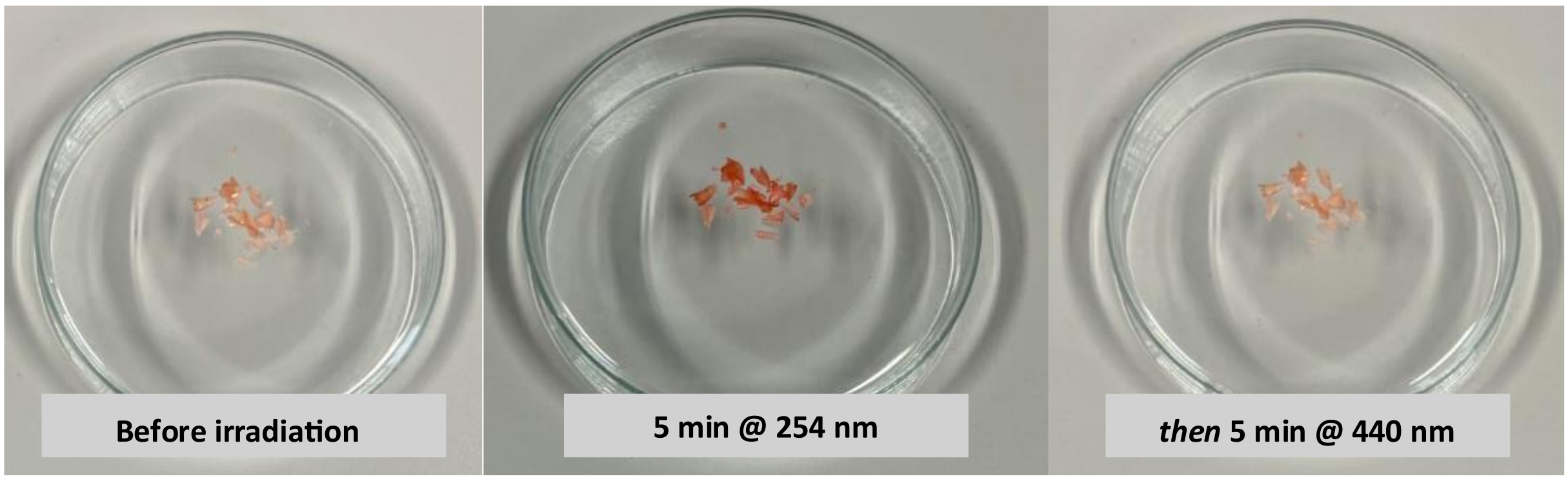

Fig. S32. Reversible photoswitching of crystalline **8**.

S8 Cyclopropene-bridged dithienylethenes

We explored the synthetic accessibility of cyclopropene-bridged dithienylethenes as well. Firstly, we attempted to analogously prepare diketone **S11** from Friedel-Crafts acylation with malonyl chloride, however this resulted in a very complex reaction mixture without a separable main product. As a result, we turned to alternative synthetic approaches.

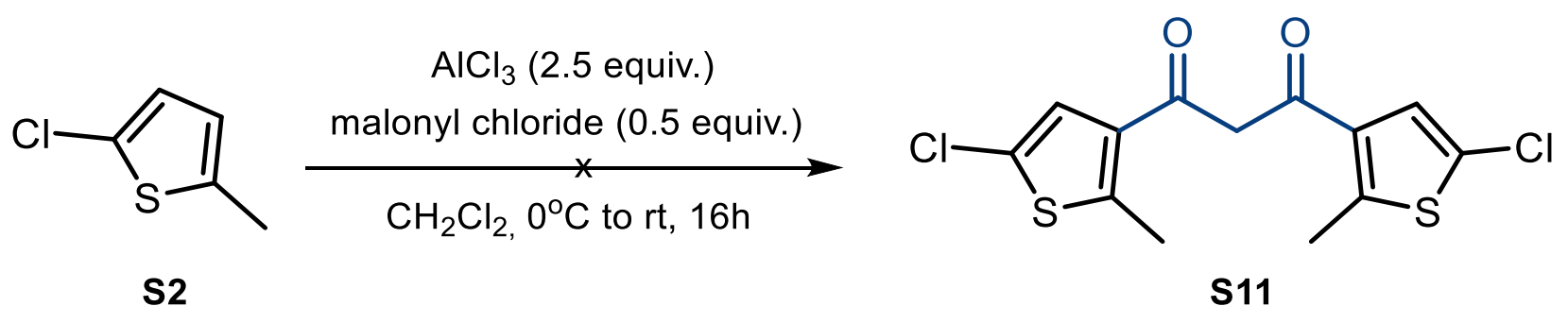

Fig. S33. Attempted synthesis of diketone **S11** via Friedel-Crafts acylation with malonyl chloride.

Subsequently, we successfully prepared Weinreb-amide **S13**, a key 1,3-dicarbonyl synthon first disclosed in this present communication. Unfortunately, diketone **S15** could not be obtained via this lithiation-Weinreb ketone synthesis sequence either.

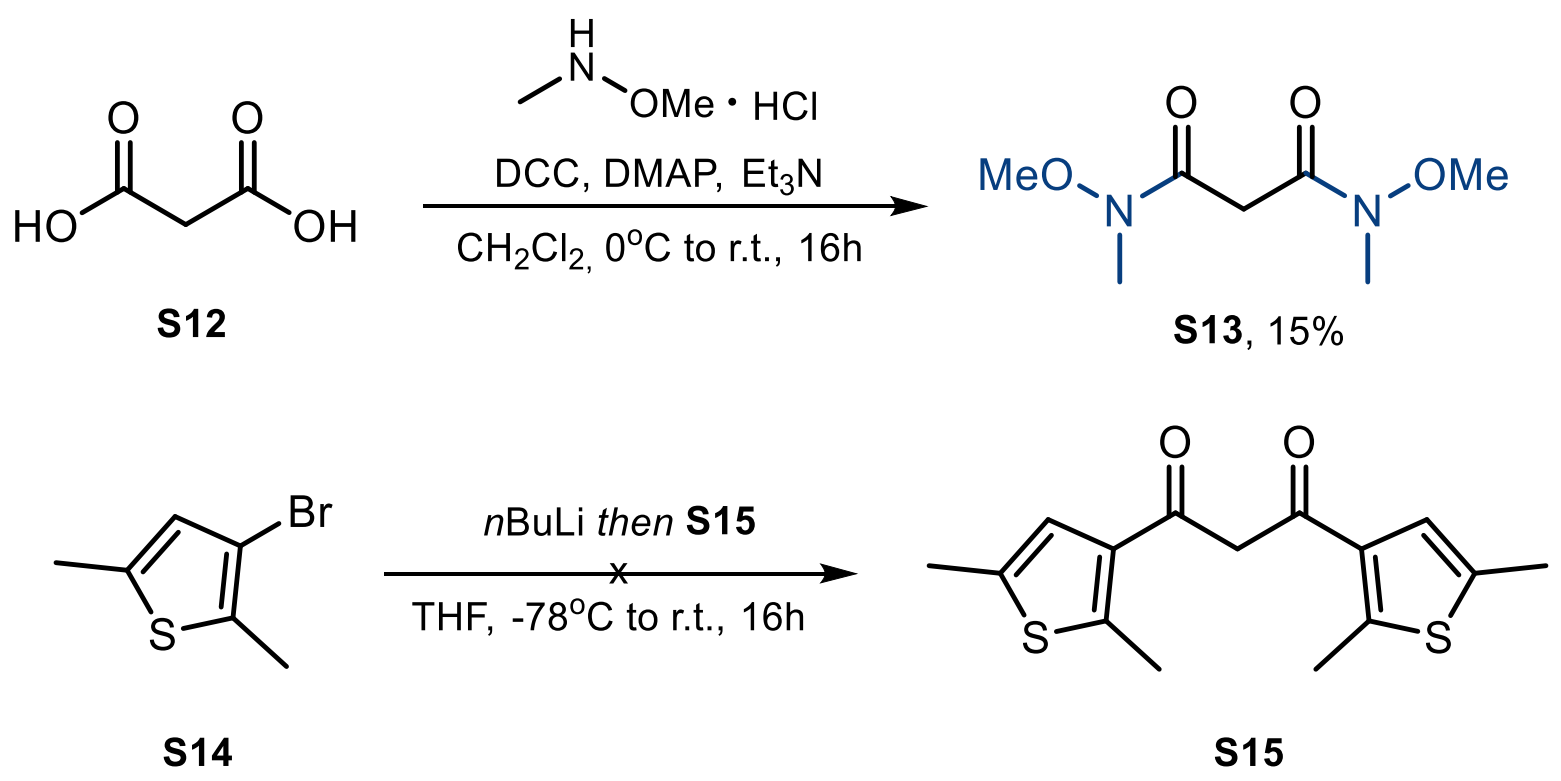

Fig. S34. Attempted synthesis of diketone **S15** via Weinreb-amide intermediate **S13**.

Following the 'dead-end' attempts, we successfully implemented different C1 insertion strategies (Fig. S35) on diaryl acetylene **14** to produce the desired cyclopropenes (**15** and **16**) as detailed in the main text. UV-vis spectroscopic and photoswitching properties of the cyclopropene-bridged dithienylethenes were also evaluated. The absorption spectra of DTE **14** and **15** showed fairly similar features, two high intensity bands at approximately 220 and 300 nm. Notably, significant solvatochromism was not experienced using a range of different solvents (MeCN, MeOH, DCM, toluene, $CHCl_3$). Irradiation with UV light (λ_{max} = 312 nm) resulted in the depletion of absorption intensity at the 300 nm peak and a slight increase in the visible range (350 – 500 nm), in both cases. However, this photoreaction was irreversible even after prolonged irradiation with different wavelengths in the visible or UV region. By following

the effect of photoirradiation on difluoro derivative **15** with NMR spectroscopy we experienced that extended UV irradiation resulted in the loss of F from the molecule (based on ^{19}F NMR).

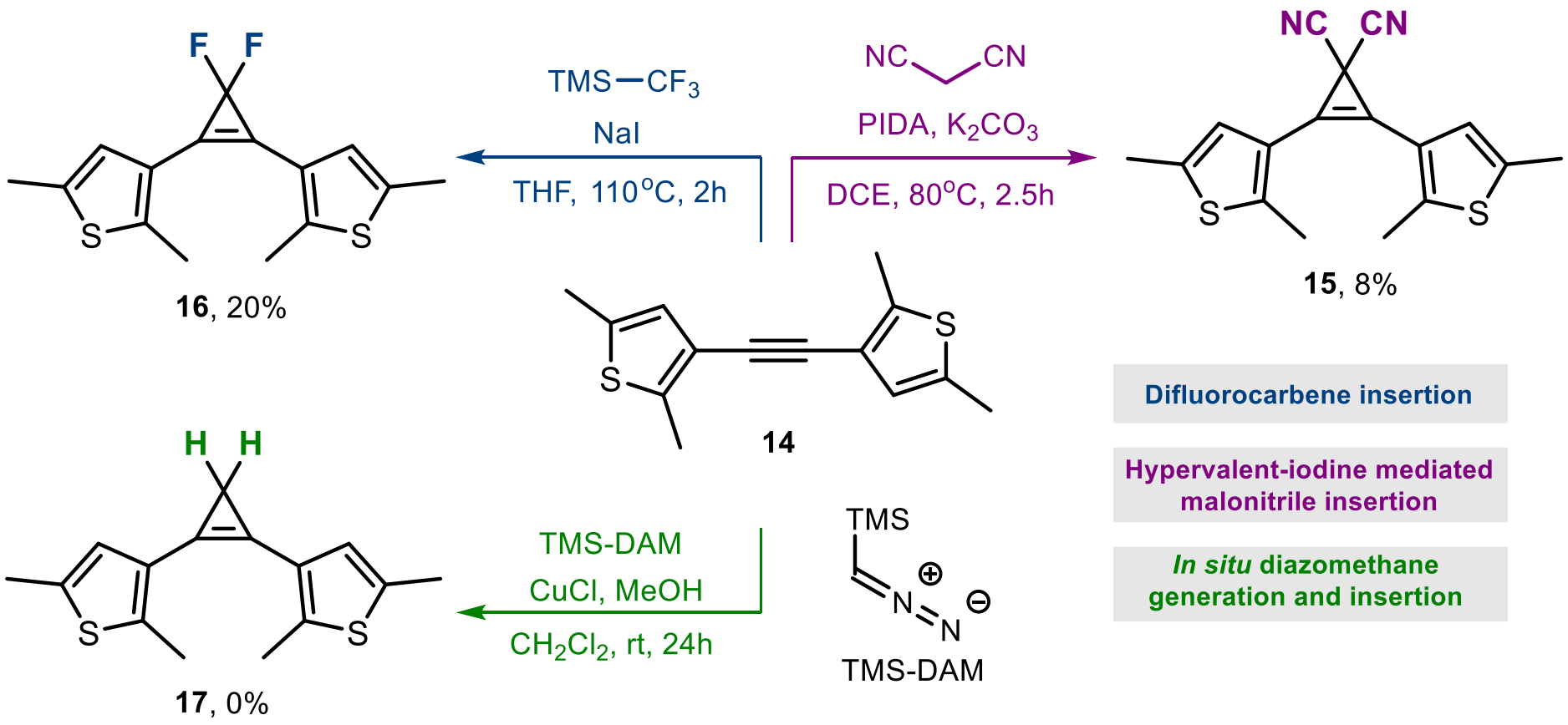

Fig. S35. Syntheses of cyclopropene-bridged dithienylethenes via C1 insertion strategy.

S8.1 Experimental Protocols and Product Characterization

Synthesis of dithienylacetylene **14** has been previously reported by our research group.[2]

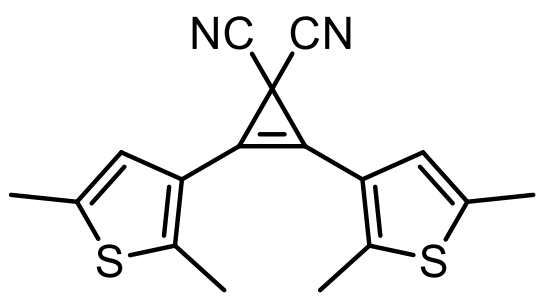

15
2,3-bis(2,5-dimethylthiophen-3-yl)cycloprop-2-ene-1,1-dicarbonitrile

Compound 15: A 4 mL scintillation vial was charged with dithienylacetylene **14** (100.0 mg, 0.41 mmol, 1.0 equiv.), PIDA (274.5 mg, 0.85 mmol, 2.1 equiv.), potassium carbonate (117.8 mg, 0.85 mmol, 2.1 equiv.), malonitrile (32.2 mg, 0.49 mmol, 1.2 equiv.) and 1,2-dichloroethane (DCE) (2 mL). The mixture was heated to 80°C and stirred for 2.5 hours. Subsequently, the mixture was cooled to room temperature, poured into water and extracted with CH_2Cl_2 (3 x 10 mL). The combined organic phase was dried over anhydrous $MgSO_4$ and concentrated under reduced pressure. The crude product was further purified by column chromatography (SiO_2, hexanes/ethyl acetate) affording the desired product as a yellowish solid. **Yield:** 8%.

Physical state: yellow, amorphous solid

^{1}H NMR ($CDCl_3$, 500 MHz): δ = 6.88 (s, 2H), 2.73 (s, 6H), 2.47 (s, 6H) ppm. (see Spectrum)

^{13}C NMR ($CDCl_3$, 125.5 MHz): δ = 142.9, 138.6, 125.8, 119.3, 117.1, 93.7, 15.3, 14.3 ppm. (see Spectrum)

Rf: 0.46 (hex/EtOAc, 9:1)

HRMS (APCI-TOF): *m/z* calcd. for $C_{17}H_{14}N_2S_2$ ($[M+H]^+$) 311.0671, found 311.0656.

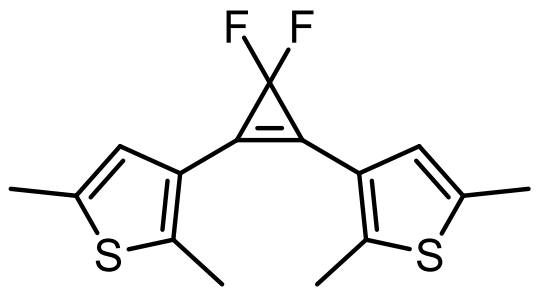

16

3,3'-(3,3-difluorocycloprop-1-ene-1,2-diyl)bis(2,5-dimethylthiophene)

Compound 16: An oven-dried Schlenk tube was charged with dithienylacetylene **14** (100.0 mg, 0.41 mmol, 1.0 equiv.), $TMSCF_3$ (115.4 mg, 0.82 mmol, 2.0 equiv.) anhydrous NaI (133.8 mg, 0.89 mmol, 2.2 equiv.) and THF (3 mL). The mixture was heated to 110°C and stirred for 2 hours. The reaction was quenched by the addition of saturated Na_2CO_3 solution at room temperature and the resulting mixture was extracted with diethyl ether (3 x 20mL). The combined organic phase was dried over anhydrous $MgSO_4$ and concentrated under reduced pressure. The crude product was further purified via column chromatography (SiO_2, hexanes/Et_3N) affording the desired product as a yellowish solid. **Yield:** 20%.

[Note]: 1,1-Diflurocyclopropenes are highly sensitive to acidic media, thus both column chromatography and pTLC were performed using hexane/Et_3N eluent. The silica column and pTLC plates were equilibrated with hexanes/Et_3N (10:1) mixture prior to separation. For the separation hexanes/Et_3N (40:1) mixture was used as eluent. NMR spectra were acquired in $CDCl_3$ pre-treated with anhydrous K_2CO_3.

Physical state: yellow, amorphous solid

^{1}H NMR ($CDCl_3$, 500 MHz): δ = 6.89 (s, 2H), 2.61 (s, 6H), 2.47 (s, 6H) ppm. (see Spectrum)

^{13}C NMR ($CDCl_3$, 125.5 MHz): δ = 149.8, 138.6, 124.7, 123.6, 29.9, 15.42, 15.19 ppm. (see Spectrum)

^{19}F NMR ($CDCl_3$, 282.4 MHz): δ: -101.02 ppm. (see Spectrum)

Rf: 0.46 (hexanes)

HRMS (APCI-TOF): *m/z* calcd. for $C_{15}H_{14}FS_2$ ($[M-F]^+$) 277.0520, found 277.0522.

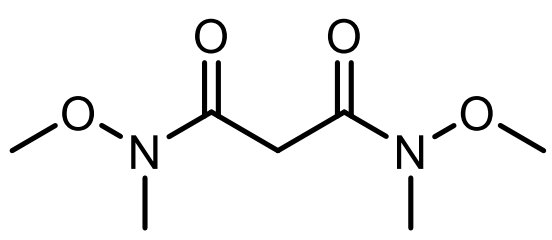

S13

N^1,*N*^{3}-dimethoxy-*N*^{1},*N*^{3}-dimethylmalonamide

Compound S13: A 100 mL round-bottom flask was charged with malonic acid (1.50 g, 14.4 mmol, 1.0 equiv.) and *N,O*-dimethylhydroxylamine hydrochloride (2.95 g, 30.3 mmol, 2.1 equiv.) and was dissolved in anhydrous CH_2Cl_2 (40 mL). The solution was cooled to 0°C and triethylamine (Et_3N) (5.84

g, 8.0 mL, 57.7 mmol, 4.0 equiv.), DMAP (176 mg, 1.44 mmol, 0.1 equiv.) and *N,N'*-dicyclohexylcarbodiimide (DCC) (6.25 g, 30.3 mmol, 2.1 equiv.) was added. The reaction was stirred overnight while warming to room temperature. After completion the mixture was diluted with CH_2Cl_2 and quenched with water. The phases were separated, and the aqueous layer was extracted with CH_2Cl_2 (3 x 30 mL). The combined organic phase was washed with saturated $NaHCO_3$ solution and brine, dried over $MgSO_4$ and was concentrated under reduced pressure. The crude product was further purified via column chromatography (SiO_2, hexanes/ethyl acetate) to afford the desired product as a yellow oil. **Yield:** 15%.

Physical state: yellow oil

^{1}H NMR ($CDCl_3$, 500 MHz): δ = 3.71 (s, 6H), 3.63 (s, 2H), 3.21 (s, 6H) ppm. (see Spectrum)

^{13}C NMR ($CDCl_3$, 125.5 MHz): δ = 168.3, 61.3, 38.4, 32.2 ppm. (see Spectrum)

Rf: 0.19 (EtOAc) (notable tailing on silica) (CAM stained)

HRMS (APCI-TOF): *m/z* calcd. for $C_7H_{15}N_2O_4$ ($[M+H]^+$) 191.1031, found 191.1023.

S8.2 UV-Vis analyses

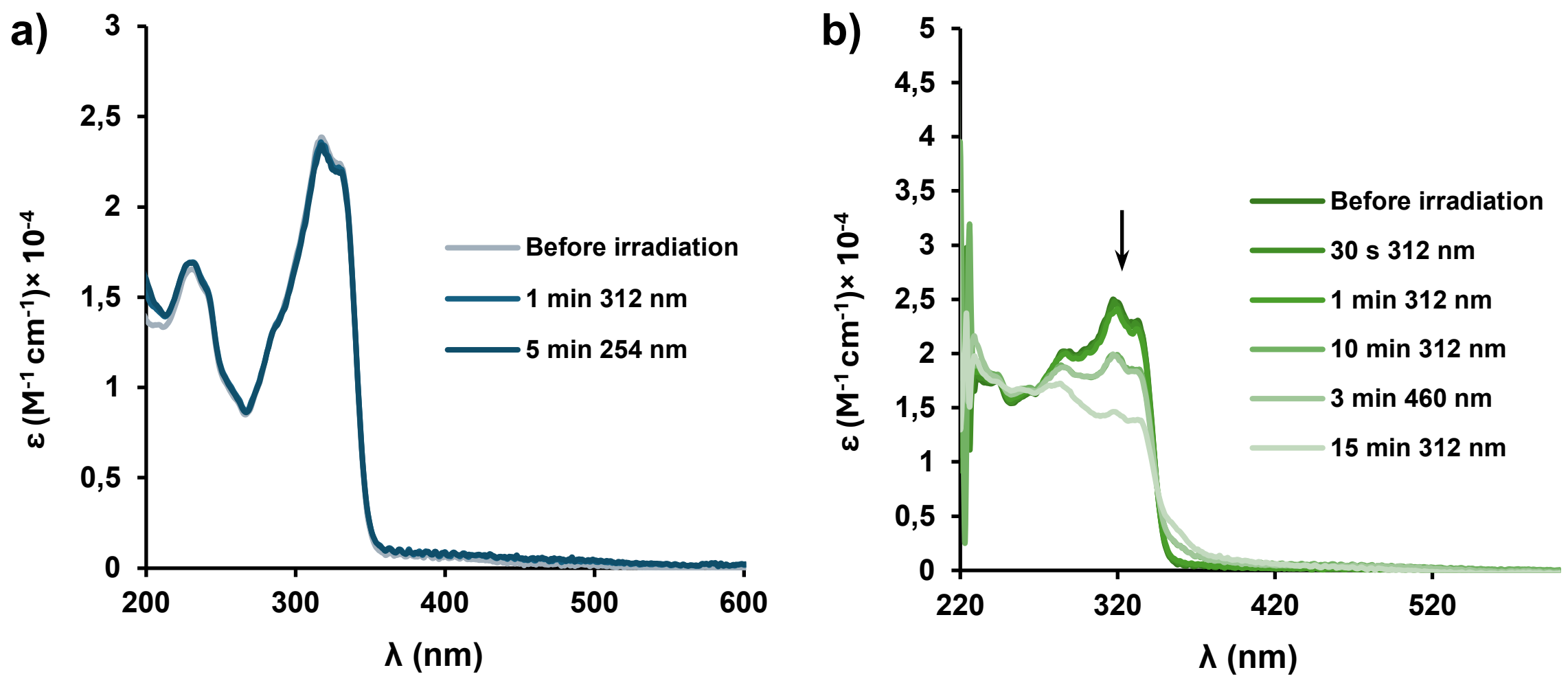

Fig. S36. Changes in the UV-vis spectra of **15** upon irradiation with UV (λ_{max} = 312 nm) light in a solution of **a)** MeCN and **b)** $CHCl_3$ (c = 3 × 10^{-5} M).

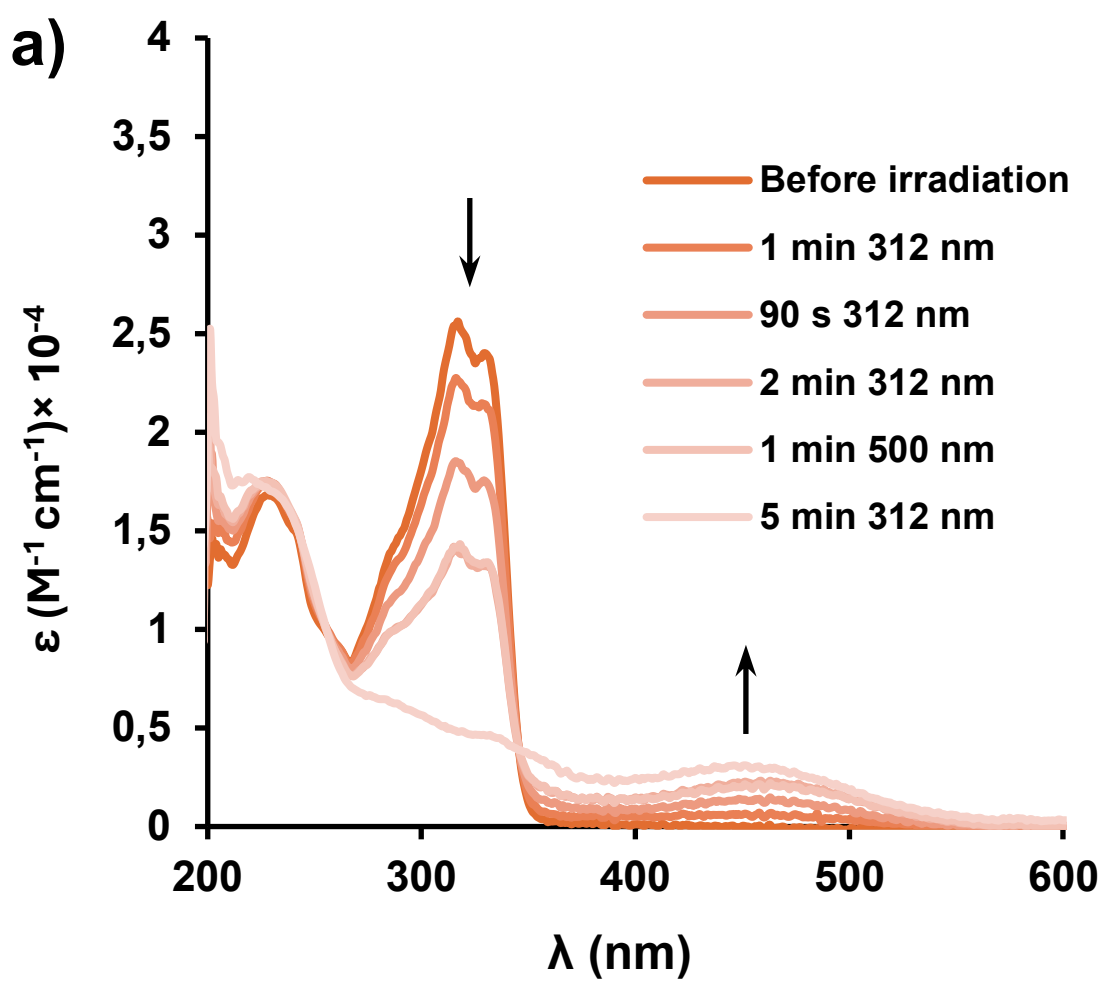

Fig. S37. Changes in the UV-vis spectra of **15** upon irradiation with UV (λ_{max} = 312 nm) light in solution (MeOH, C = 3 × 10^{-5} M).

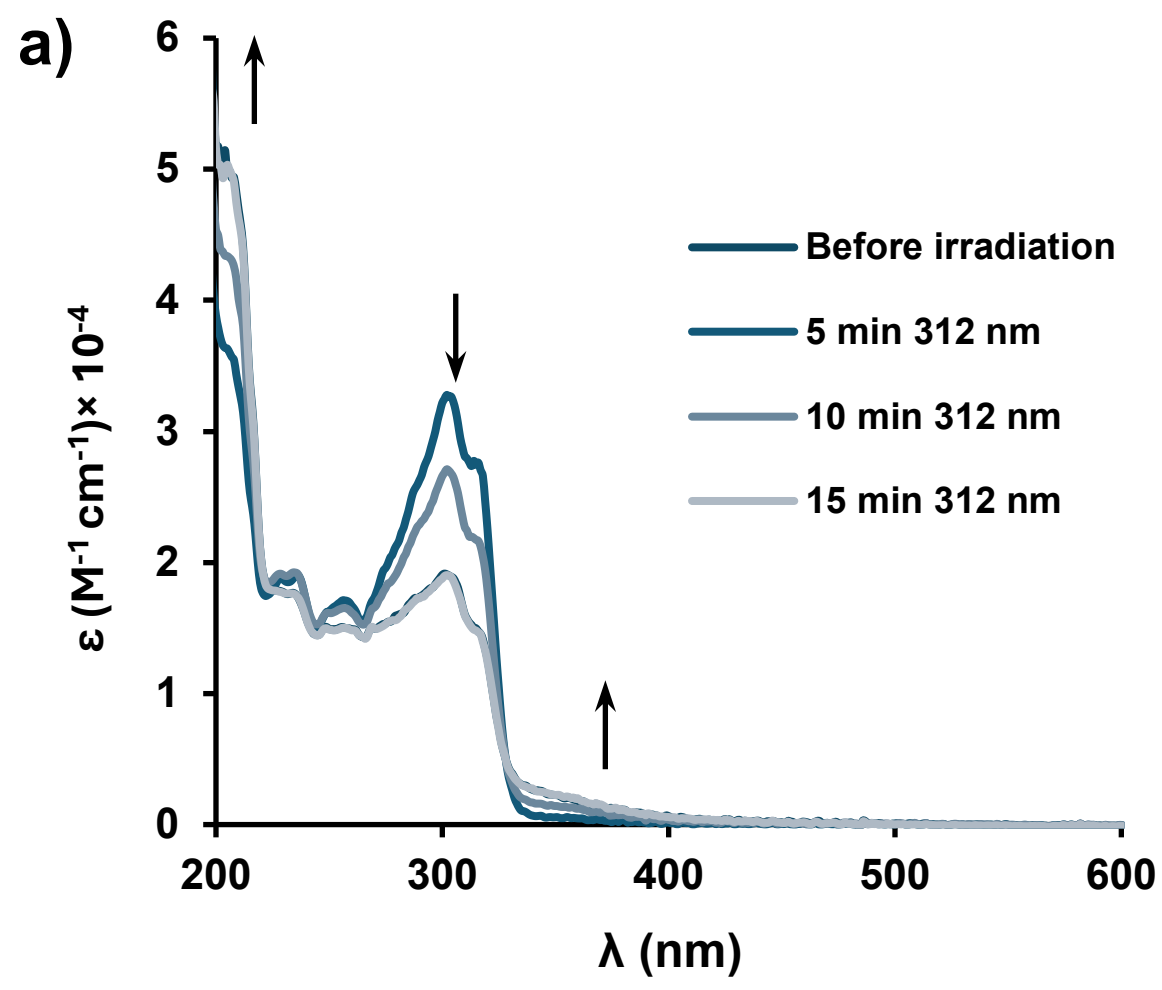

Fig. S38. Changes in the UV-vis spectra of **16** upon irradiation with UV (λ_{max} = 312 nm) light in a solution of **a)** MeCN and **b)** chloroform (c = 3 × 10^{-5} M).

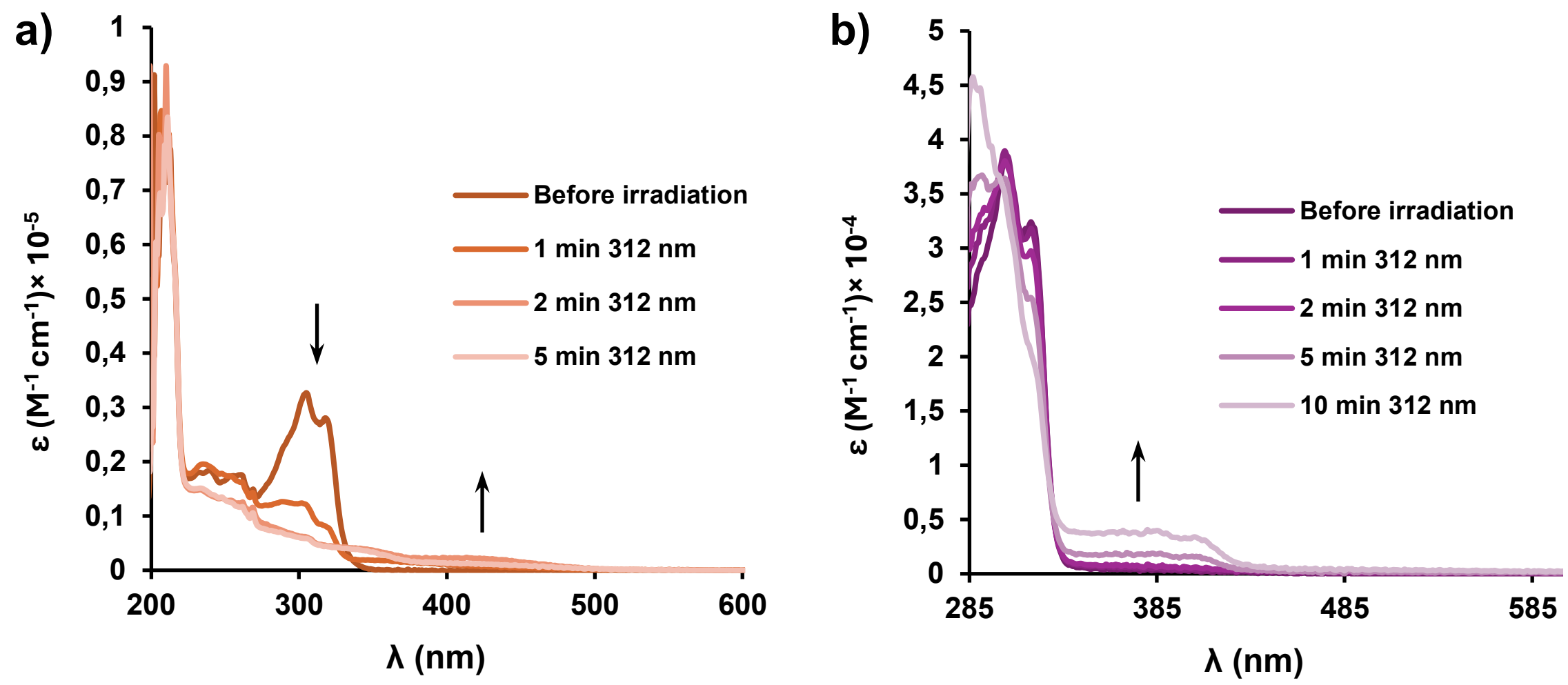

Fig. S39. Changes in the UV-vis spectra of **16** upon irradiation with UV (λ_{max} = 312 nm) light in a solution of **a)** MeOH and **b)** toluene (c = 3 × 10^{-5} M).

S9 LED emission spectra

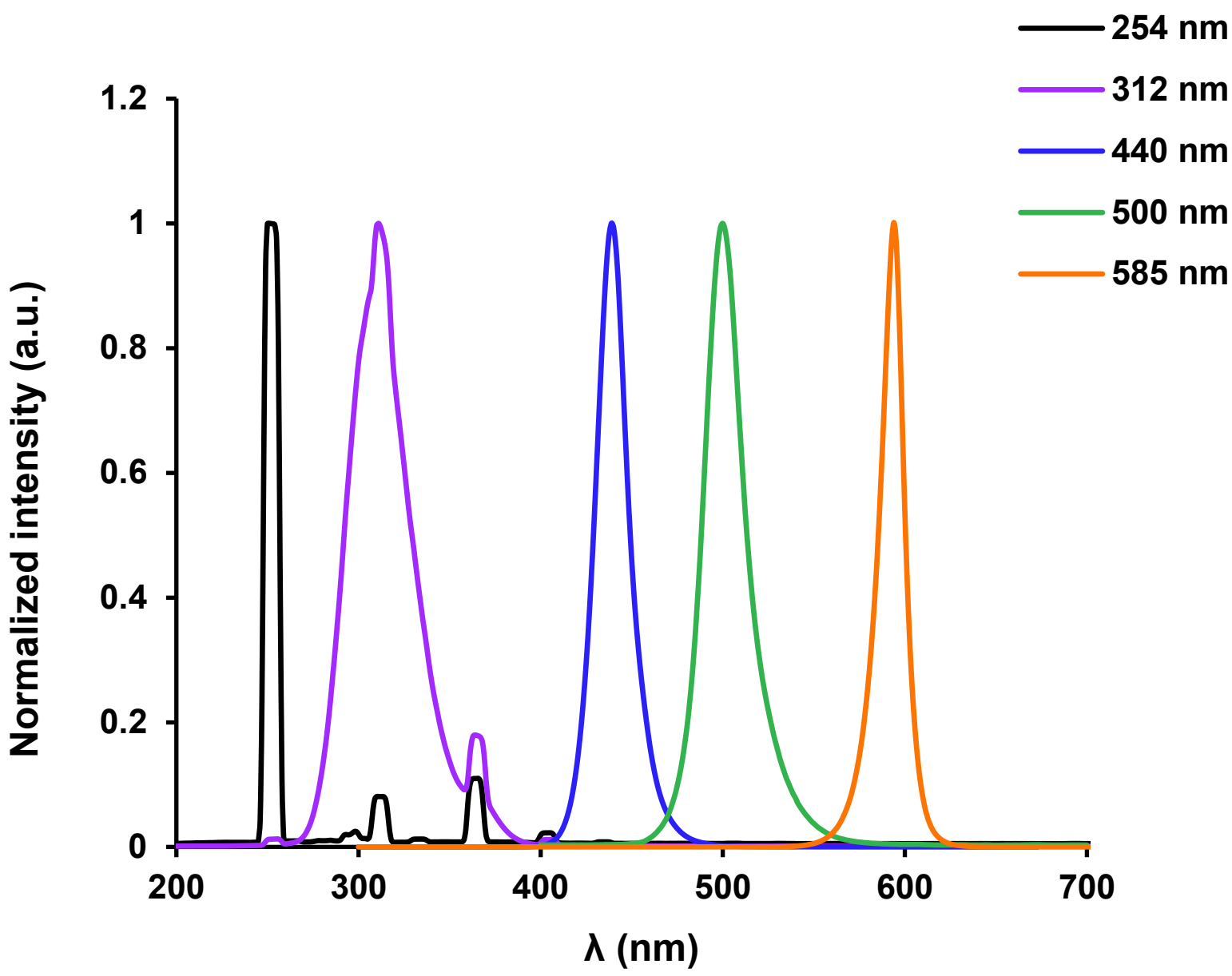

Fig. S40. Emission spectra of LED sources used in this work measured on a Jasco FP 8300 spectrofluorometer.

S10 Background

This next section provides additional insights regarding different important aspects of the main text.

S10.1 Previous uses of $TiCl_4$(collidine)

Titanium tetrachloride is typically available as colorless-yellow, volatile liquid for organic synthesis. It is extremely sensitive to water, upon contact with humid air it forms thick smoke of titanium dioxide (TiO_2) and hydrochloric acid (HCl). This extreme reactivity requires rigorous precautions and intensive attention when working with $TiCl_4$ making laboratory use quite labor-intensive. On the other hand, the versatile reactivity of this compound offers a wide range of synthetic transformations capitalizing on its Lewis-acidity, oxophilicity and potency in one-electron reductions. The Lewis acidic character also comes with the tendency to form complexes, as a result numerous ligands have been studied in $TiCl_4$ complexes (e.g. Cp_2TiCl_2, $TiCl_4(THF)_2$, arene complexes). Organic amines also react with $TiCl_4$ to give complexes such as $TiCl_4$(*collidine*). This complex (yellow, solid) exhibits outstanding stability, therefore can be utilized as a masked precursor of $TiCl_4$ for simplified handling and reaction setup. Previously it had been used as a single-electron reductant for benzyl and allyl alcohols (Fig. S41).[1,3,4]

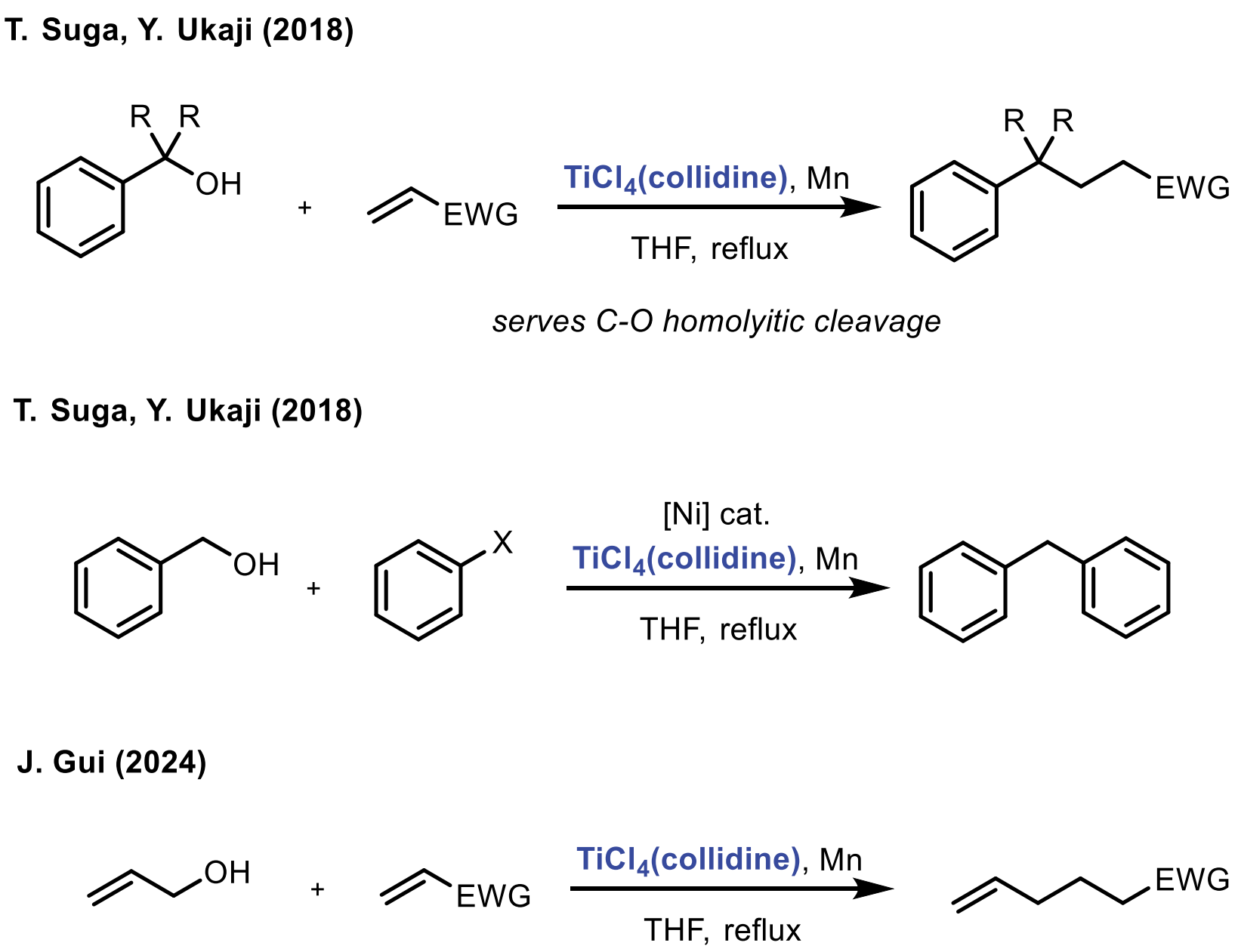

Fig. S41. Previous uses of $TiCl_4$(*collidine*) in organic synthesis.

S10.2 Photochemical side-reactivity of DTEs

Photochemical side-reactions of different diarylethenes have been reported and studied extensively before[5], including oxidations and eliminations of the closed ring isomers. A characteristic side-reactivity of perhydro dithienylethenes lead to the formation of the annulated byproduct **S16**. Mechanistic studies suggest two distinct pathways for the formation of **S16**, upon UV light irradiation of the closed ring isomer (Fig. S42.).[6]

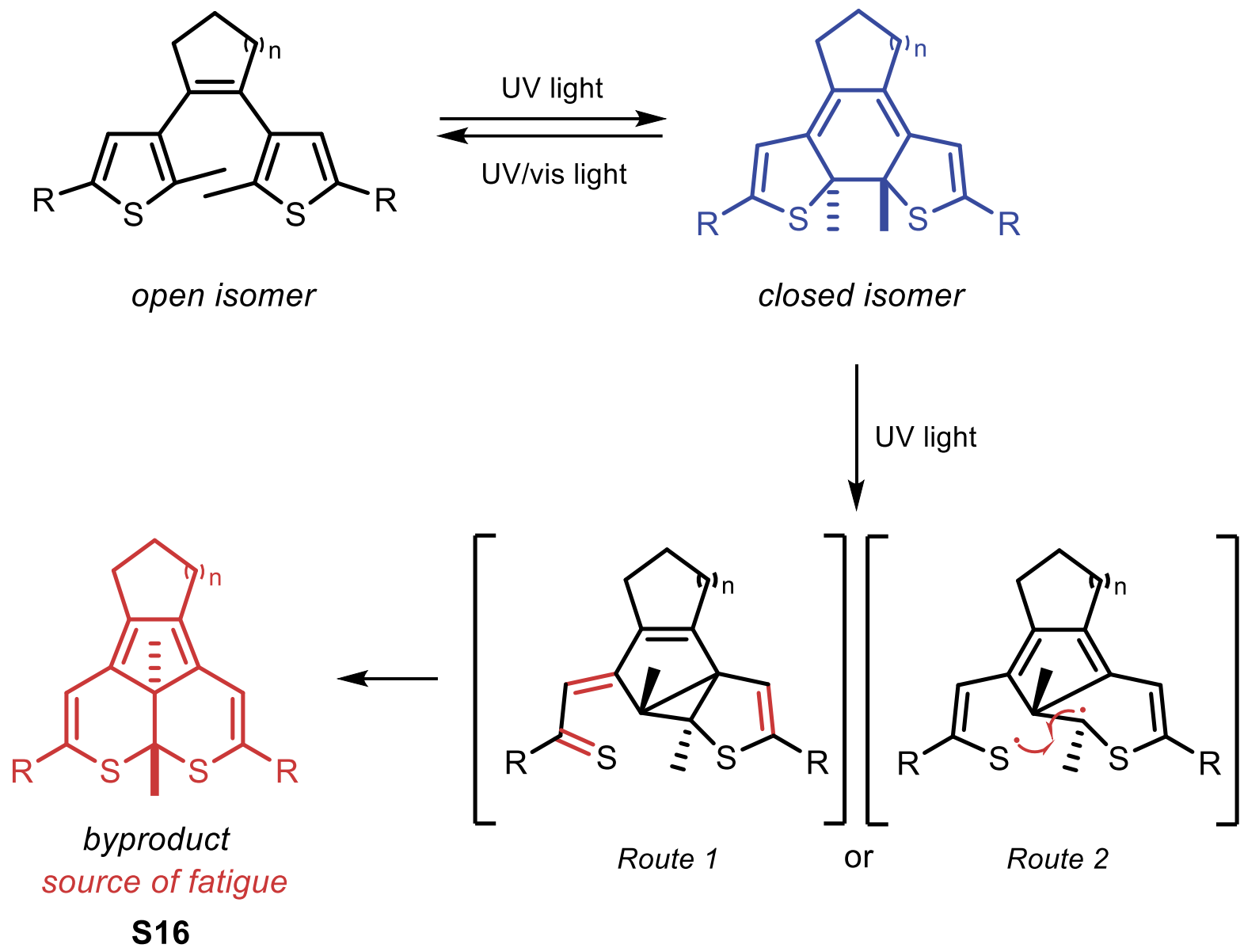

Fig. S42. Photochemical side-reactivity of dithienylethene photoswitches (formal 1,2-dyotropic rearrangement).

Byproduct formation can be monitored using *in situ* photo-NMR or HPLC measurements, however characteristic bands in the visible region of the UV/vis spectra and loss of reversibility also indicate the presence of **S16**.

S10.3 Improving fatigue resistance of DTEs

When applying molecular switches as functional units in light-responsive materials one of the crucial properties of the system is the reversibility between the two states. This is often regarded as fatigue resistance, implying stability over the course of a large number of photoisomerizations. To investigate the fatigue behavior of a new photochromic switch one performs a set of switching cycles consisting of multiple interconversions of the two states (open/closed isomer). One of the main sources of fatigue is often attributed to the formation of different byproducts, such as **S16** described above. A range of approaches have been described to suppress the photo-reactivity leading to **S16**, via core structural modifications. Novel molecular designs involve electron-withdrawing groups (e.g. CHO, Ph,

N-Me-pyridine)[7] or aromatic substituents (π-extensions) on the thiophene rings[8] to construct highly fatigue-resistant systems. Modification of the cyclic ethene bridge also holds further possibilities of improving fatigue resistance, however in case of perhydro-cycloalkene derived diarylethenes this has not been explored. Notably, this third approach offers the greatest extent of structural plasticity for late-stage functionalization.

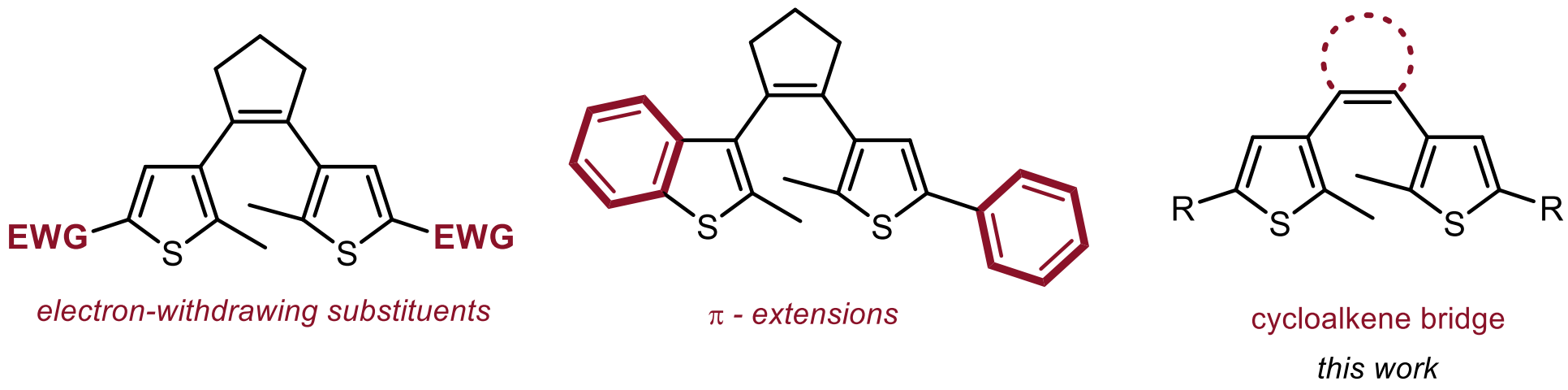

Fig. S43. Different approaches to improve fatigue resistance of diarylethene photochromic switches.

S10.4 Limitations and alternatives

Alternative Ti(IV) reagents

Alternative solutions could be argued against the use of $TiCl_4$(*collidine*), such as other commercial $TiCl_4$ complexes, the use of diluted, anhydrous $TiCl_4$ solution or glovebox conditions. Although all these approaches may simplify reaction setup $TiCl_4$(*collidine*) stands out as a combination of multiple positive traits: it is bench-stable, solid, cheap and easily accessible; it incorporates collidine as multipurpose ligand and additive.

Limitations

Intermolecular (dimerization) and cross-McMurry couplings both have been attempted with the optimized conditions but failed to give the desired products.

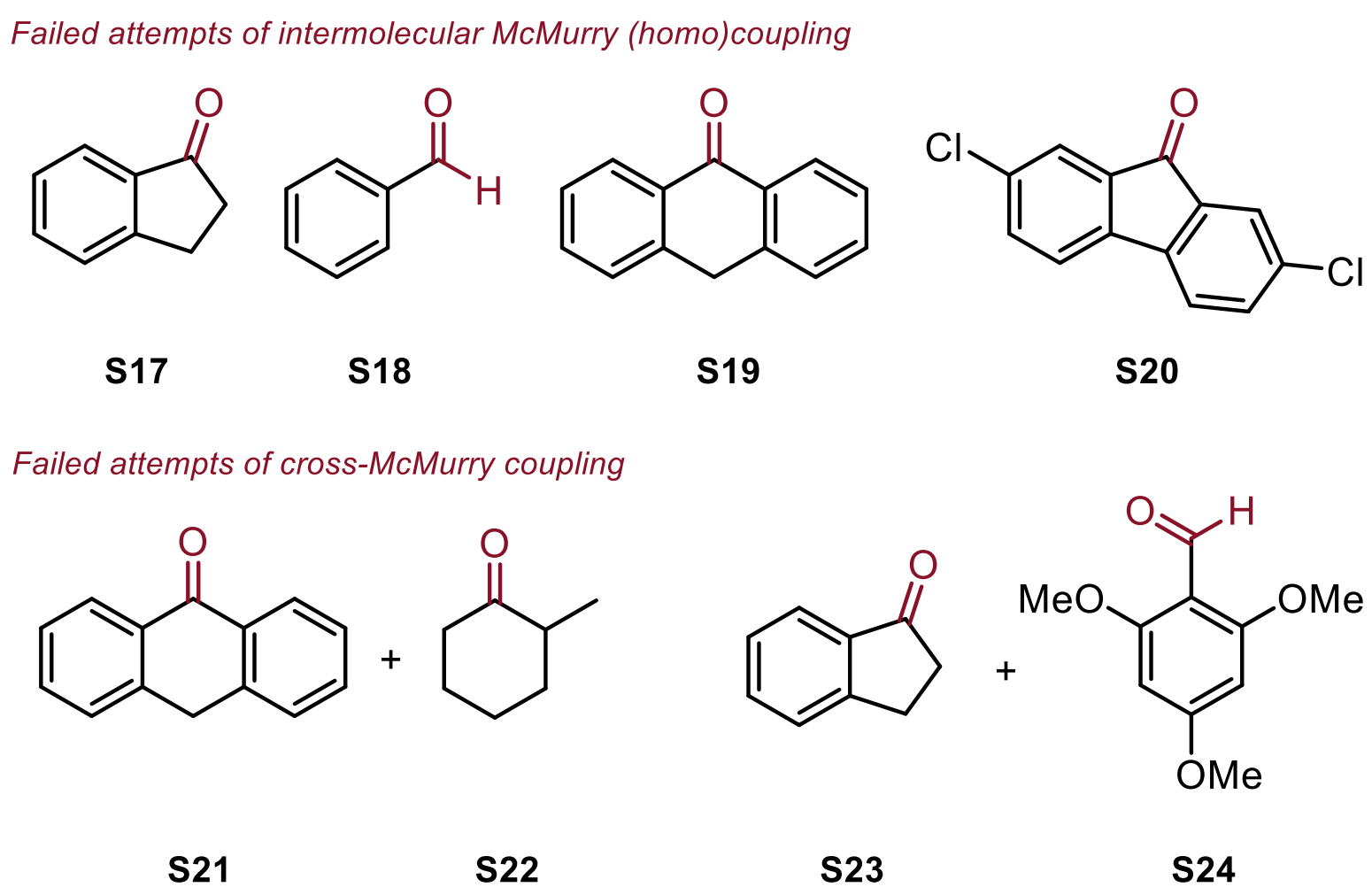

Fig. S44. Failed attempts of intermolecular McMurry reactions.

S11 References

[1] L. Yao, J. Bao, Y. Wang, J. Gui, Titanium-Mediated Dehydroxylative Cross-Coupling of Allylic Alcohols with Electron-Deficient Olefins. *Org. Lett*. **2024**, *26*, 1243–1248.

[2] P. P. Kalapos, P. J. Mayer, T. Gazdag, A. Demeter, B. Oruganti, B. Durbeej, G. London, Photoswitching of Local (Anti)Aromaticity in Biphenylene Based Diarylethene Molecular Switches. *J. Org. Chem*. **2022**, *87*, 15, 9532–9542.

[3] T. Suga, Y. Ukaji, Nickel Catalyzed Cross-Electrophile Coupling between Benzyl Alcohols and Aryl Halides Assisted by Titanium Co-reductant. *Org. Lett*. **2018**, *20*, 7846–7850.

[4] T. Suga, S. Shimazu, Y. Ukaji, Low-Valent Titanium Mediated Radical Conjugate Addition Using Benzyl Alcohols as Benzyl Radical Sources. *Org. Lett*. **2018**, *20*, 5389–5392.

[5] M. Irie, T. Lifka, K. Uchida, S. Kobatake, Y. Shindo, Fatigue Resistant Properties of Photochromic Dithienylethenes: By-product Formation. *Chem. Commun.* **1999**, *8*, 747–750.

[6] K. Higashiguchi, K. Matsuda, S. Kobatake, T. Yamada, T. Kawai, M. Irie, Fatigue Mechanism of Photochromic 1,2-Bis(2,5-dimethyl-3-thienyl)perfluorocyclopentene. *Bull. Chem. Soc. Jpn.* **2000**, *73*, 2389–2394.

[7] Y. C. Jeong, S. I. Yang, E. Kim, K. H. Ahn, Development of Highly Fluorescent Photochromic Material with High Fatigue Resistance. *Tetrahedron* **2006**, *62*, 5855–5861.

[8] M. Herder, B. M. Schmidt, L. Grubert, M. Pätzel, J. Schwarz, S. Hecht, Improving the Fatigue Resistance of Diarylethene Switches. *J. Am. Chem. Soc*. **2015**, *137*, 2738–2747.

S12 NMR spectra

Compound **S2** – ^{1}H, $CDCl_3$, 500 MHz (see Procedure)

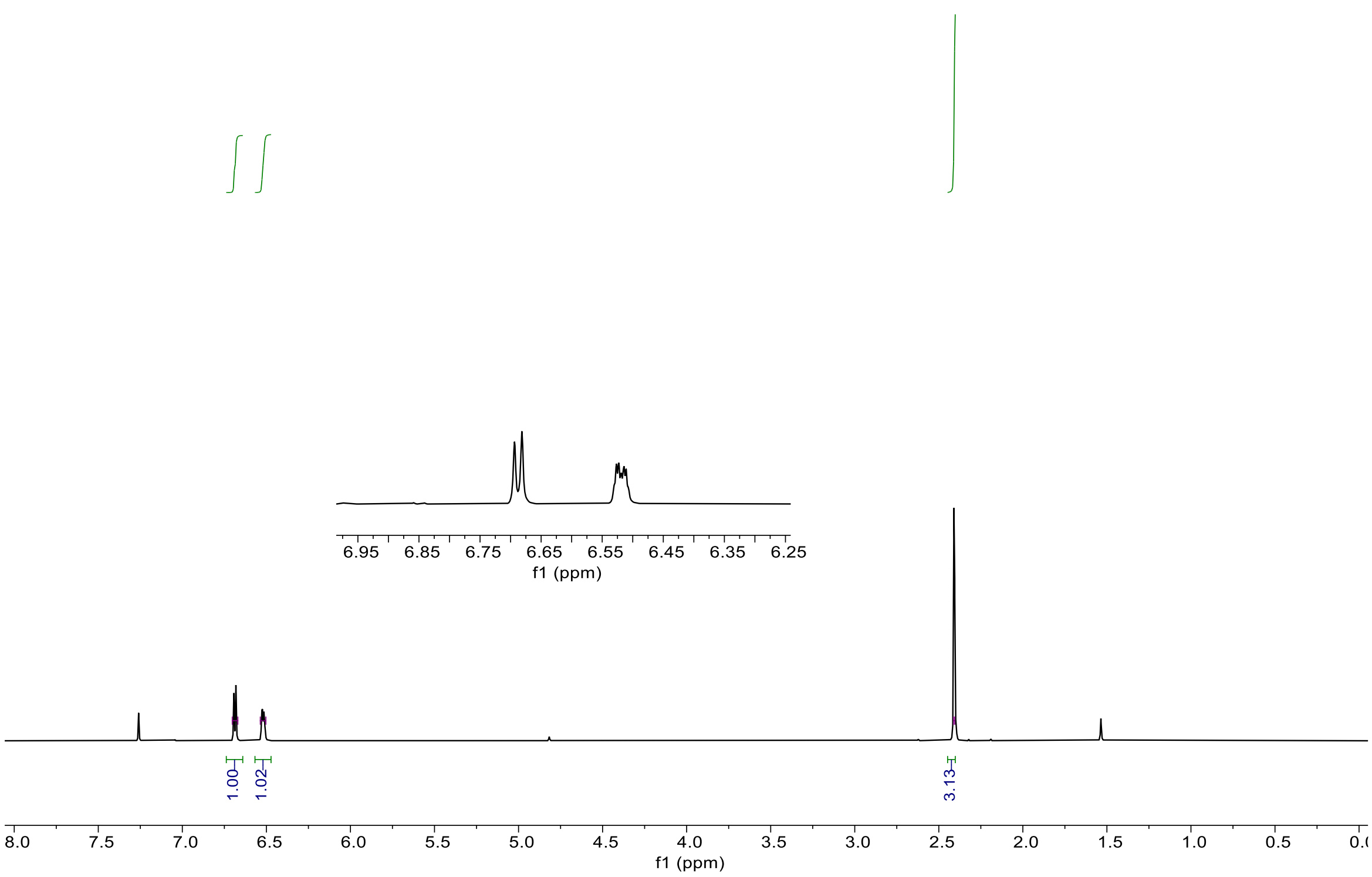

Compound **S2** – ^{13}C, $CDCl_3$, 125.5 MHz (see Procedure)

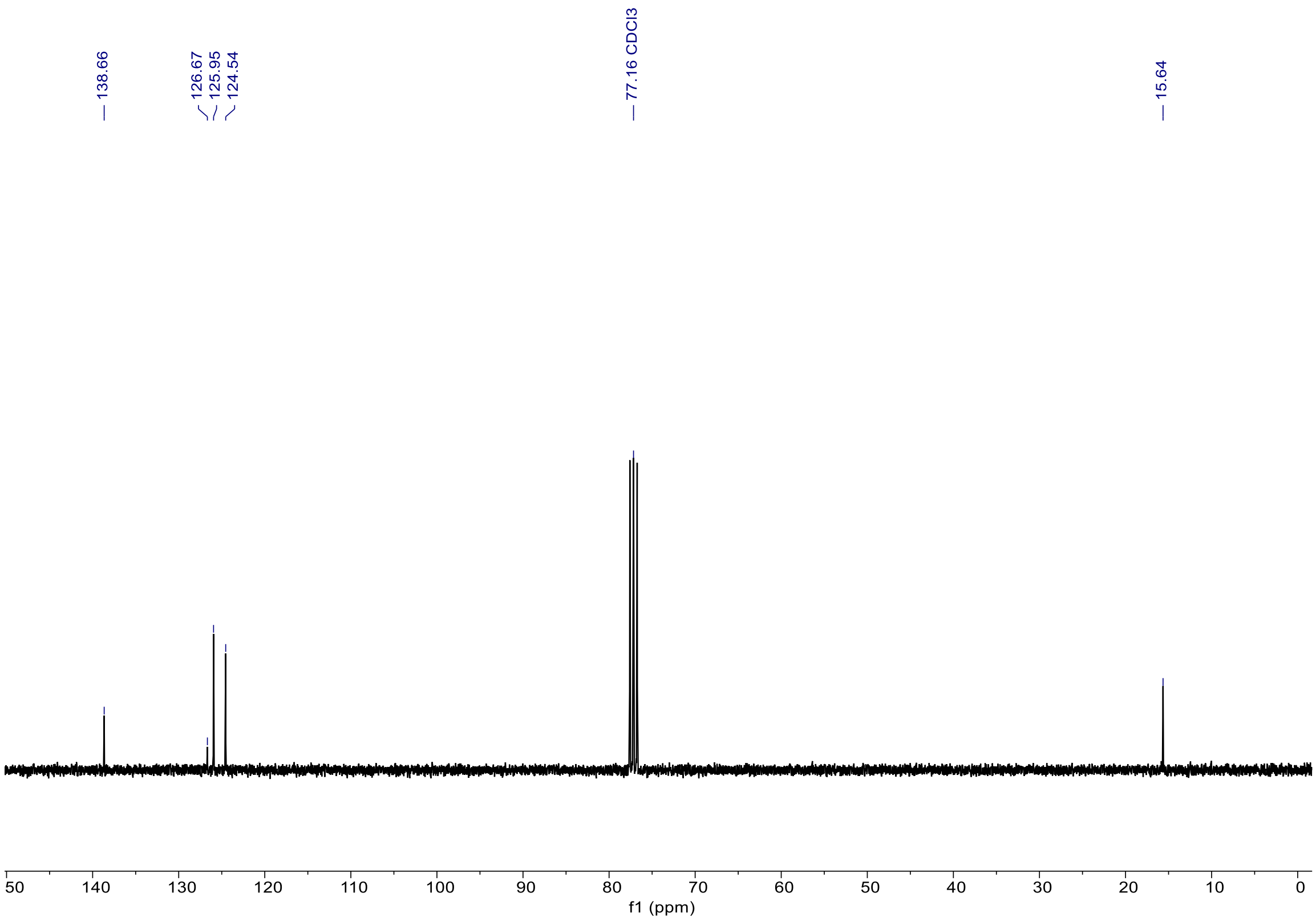

Compound **S3** – ^{1}H, $CDCl_3$, 500 MHz (see Procedure)

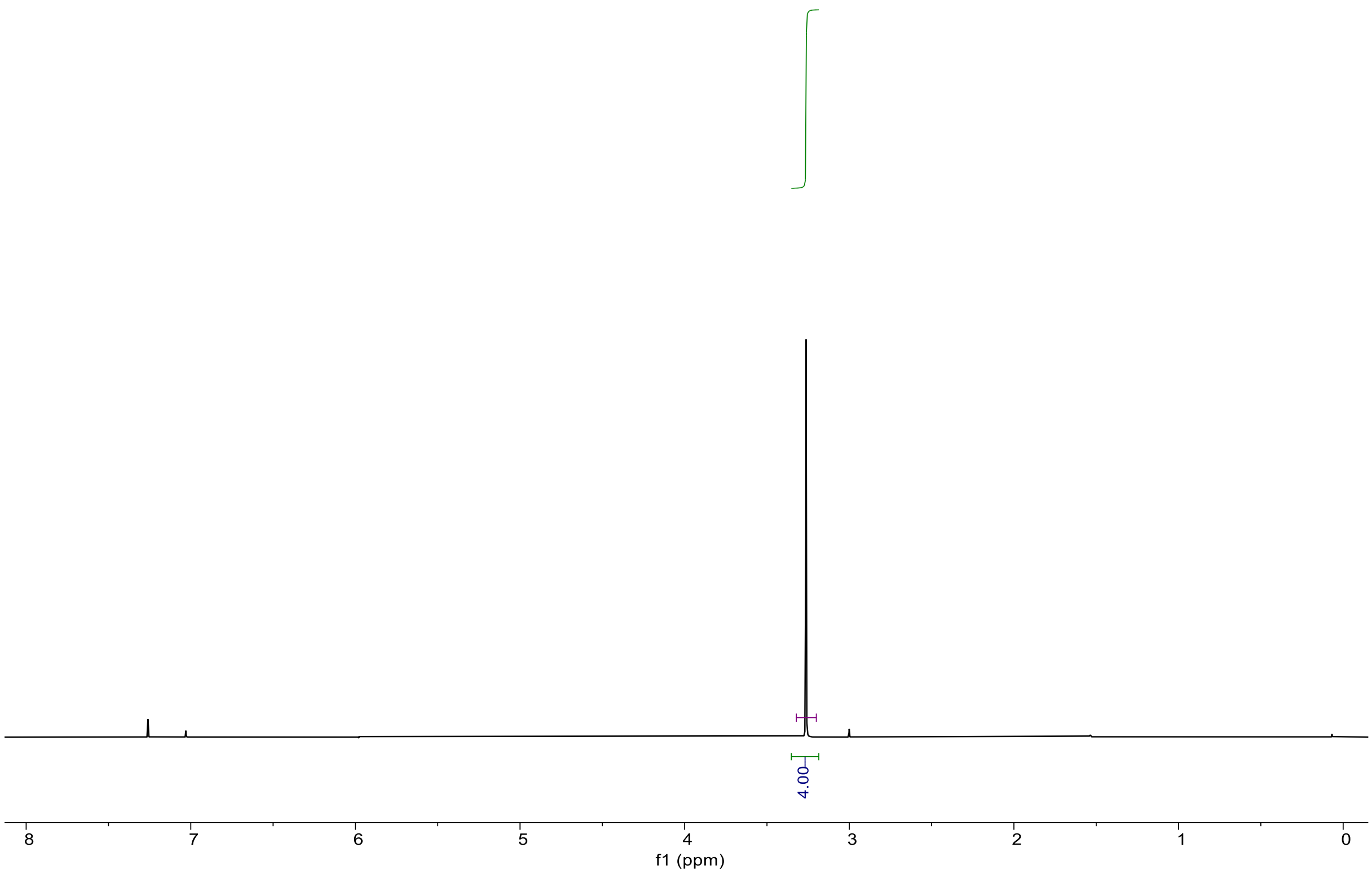

Compound **S3** – ^{13}C, $CDCl_3$, 125.5 MHz (see Procedure)

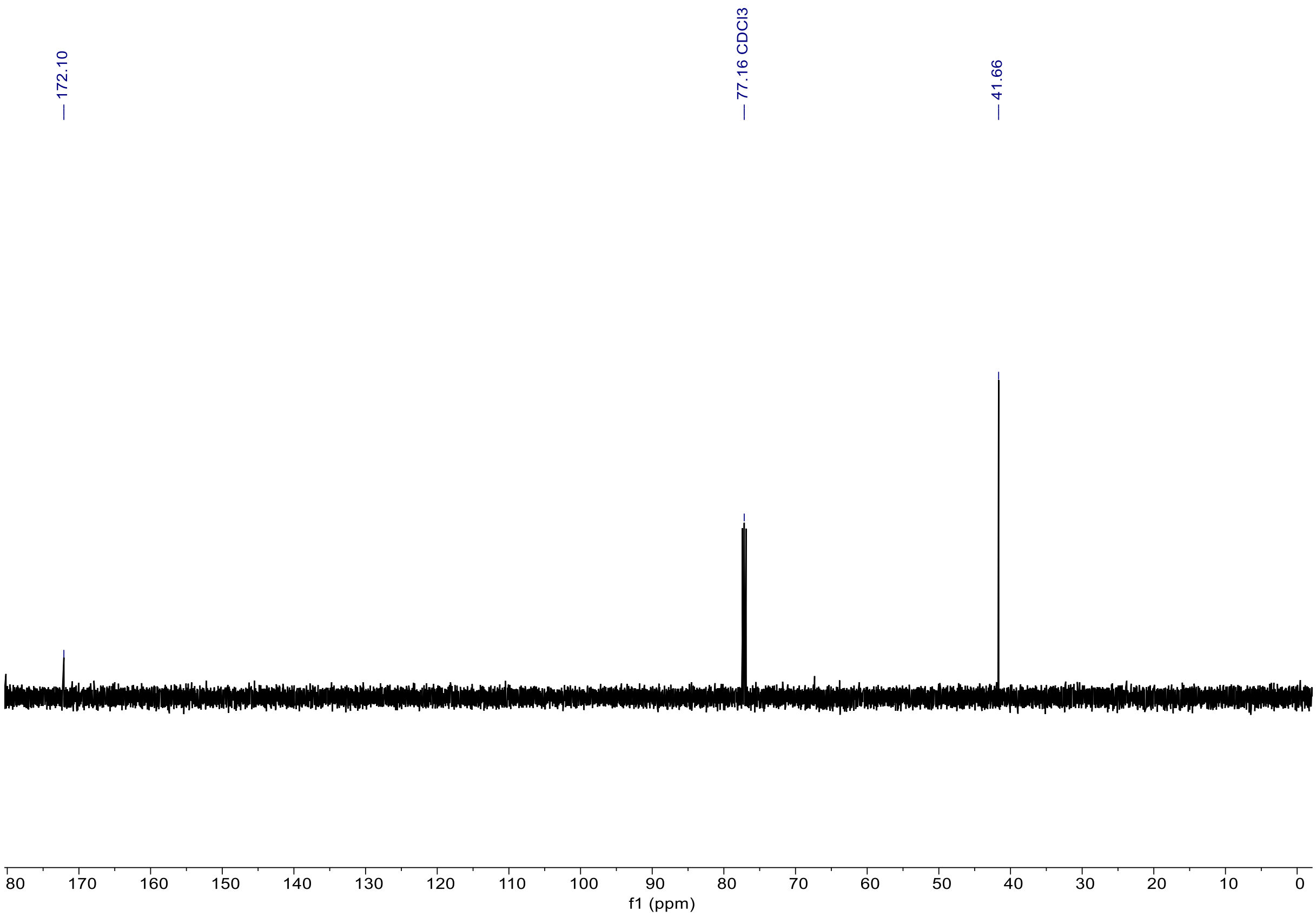

Compound **S4** – ^{1}H, $CDCl_3$, 500 MHz (see Procedure)

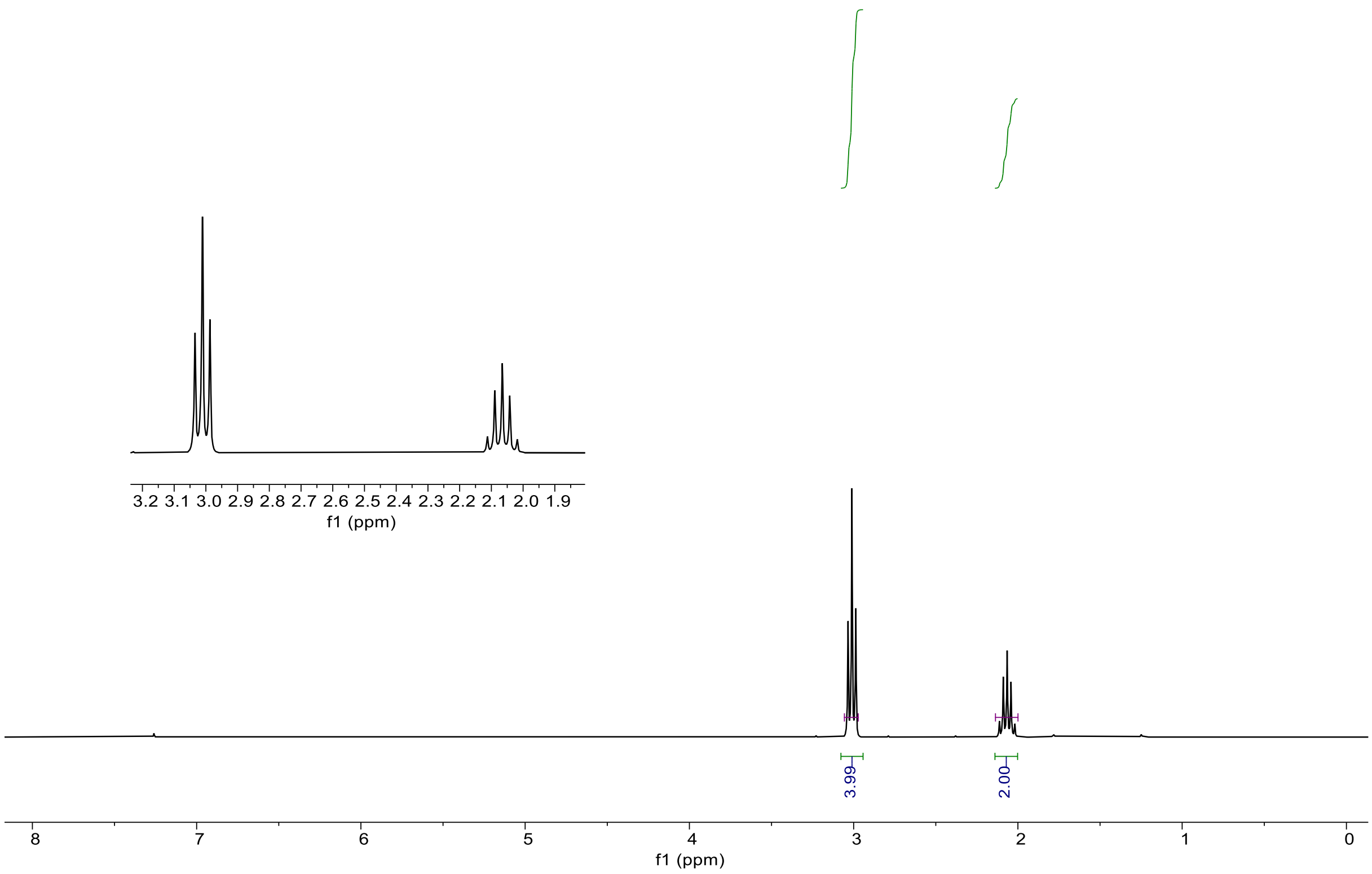

Compound **S4** – ^{13}C, $CDCl_3$, 125.5 MHz (see Procedure)

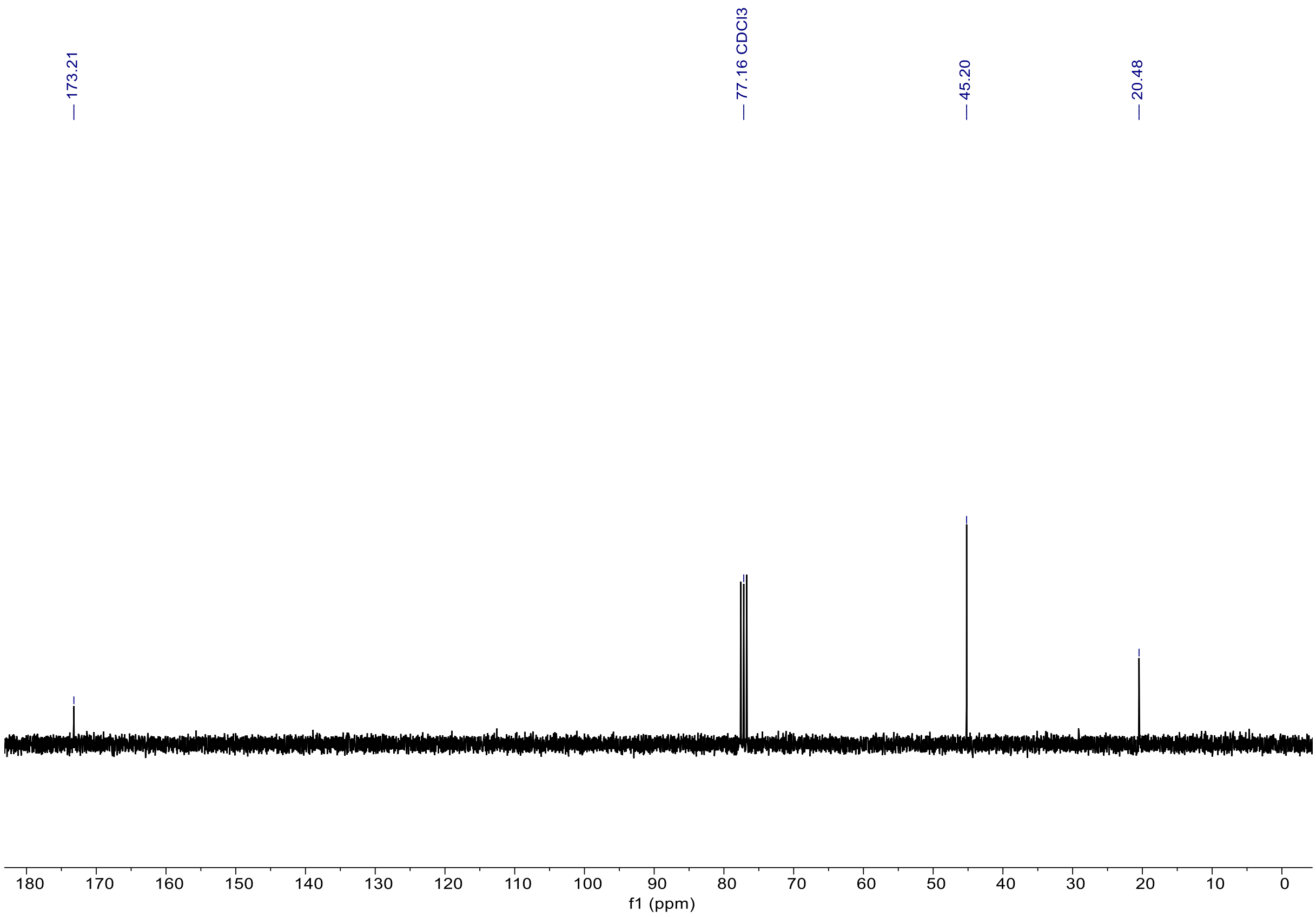

Compound **3** – ^{1}H, $CDCl_3$, 500 MHz (see Procedure)

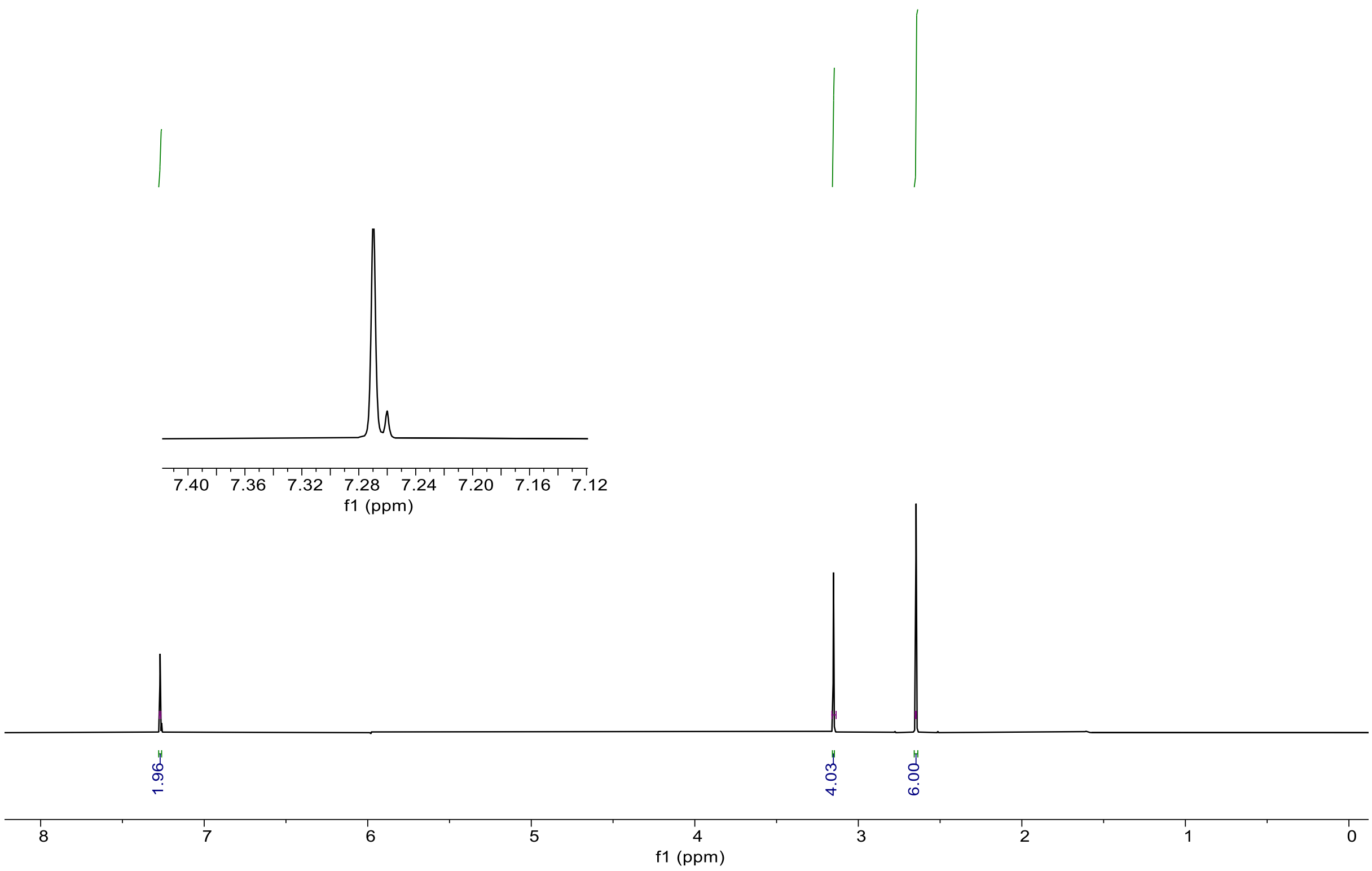

Compound **3** – ^{13}C, $CDCl_3$, 125.5 MHz (see Procedure)

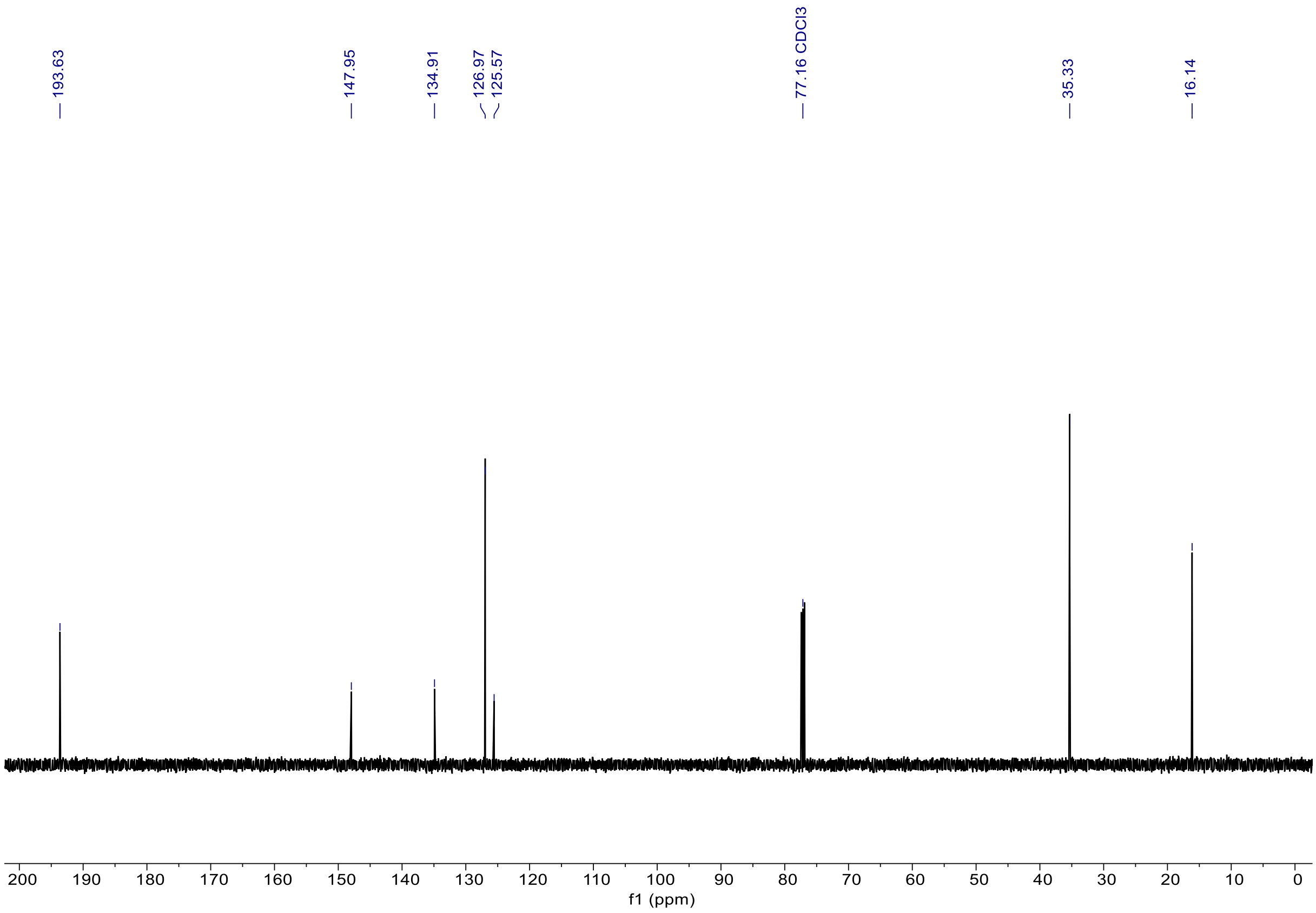

Compound **4** – ^{1}H, $CDCl_3$, 500 MHz (see Procedure)

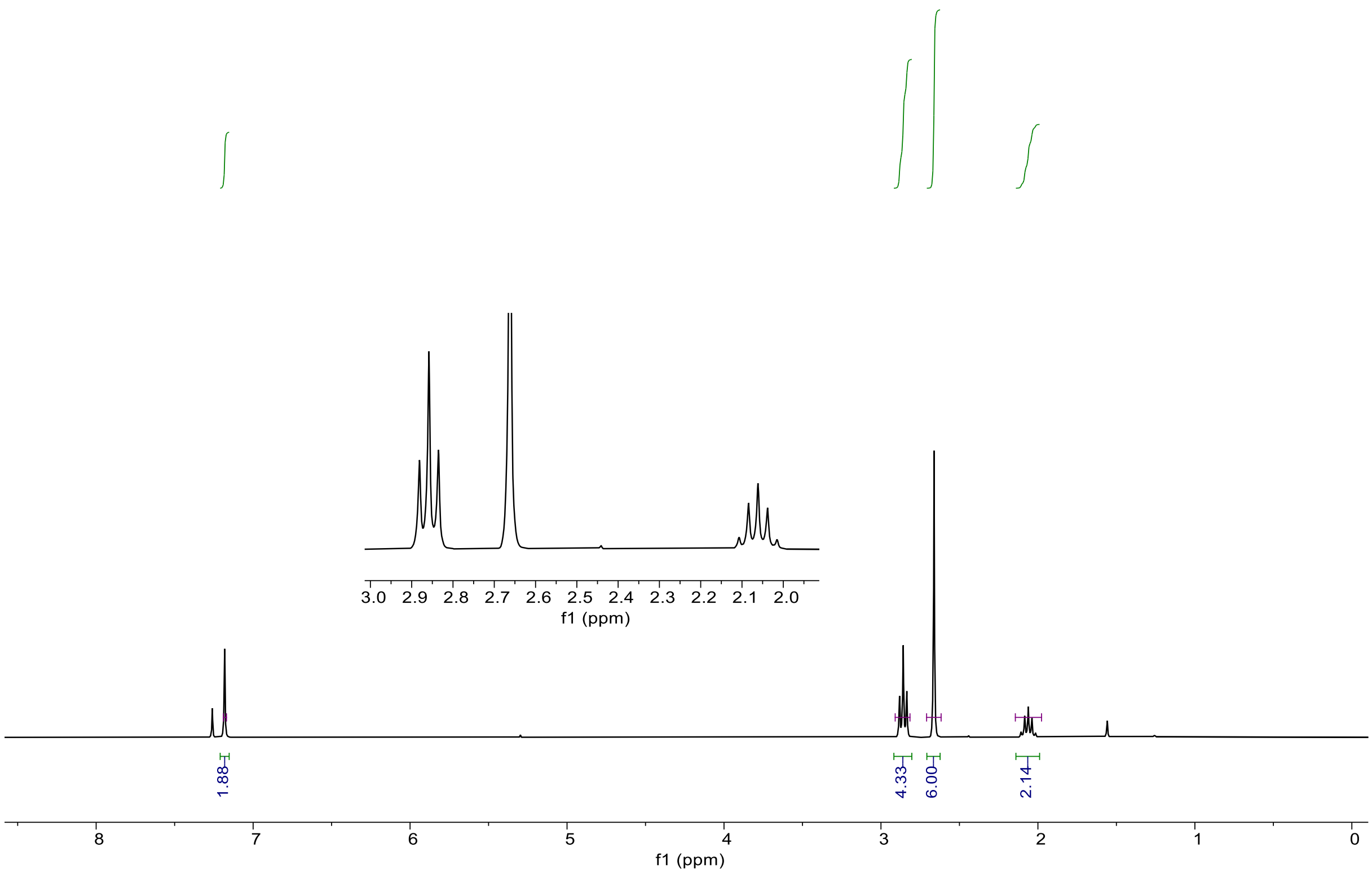

Compound **4** – ^{13}C, $CDCl_3$, 125.5 MHz (see Procedure)

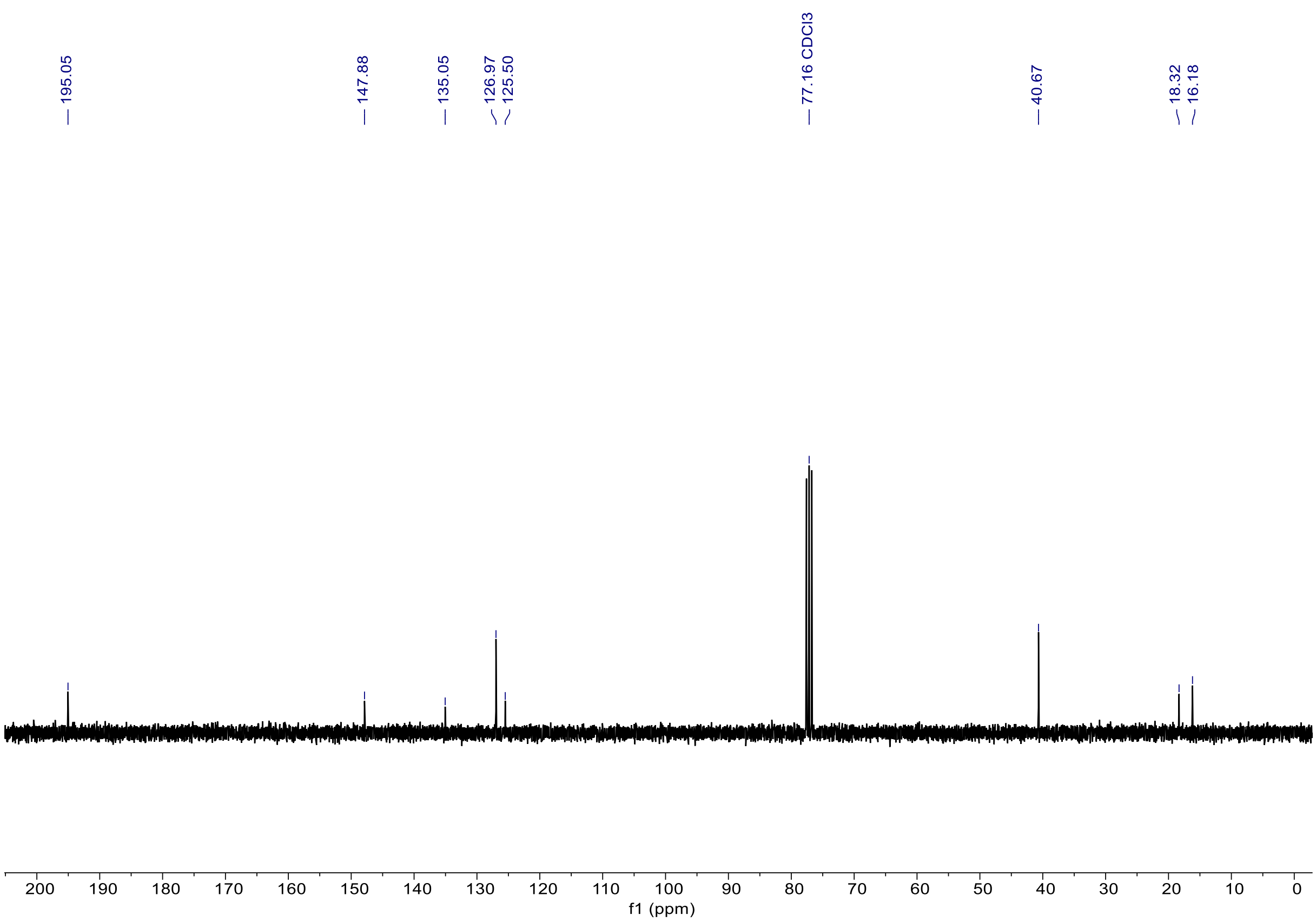

Compound **5** – ^{1}H, $CDCl_3$, 500 MHz (see Procedure)

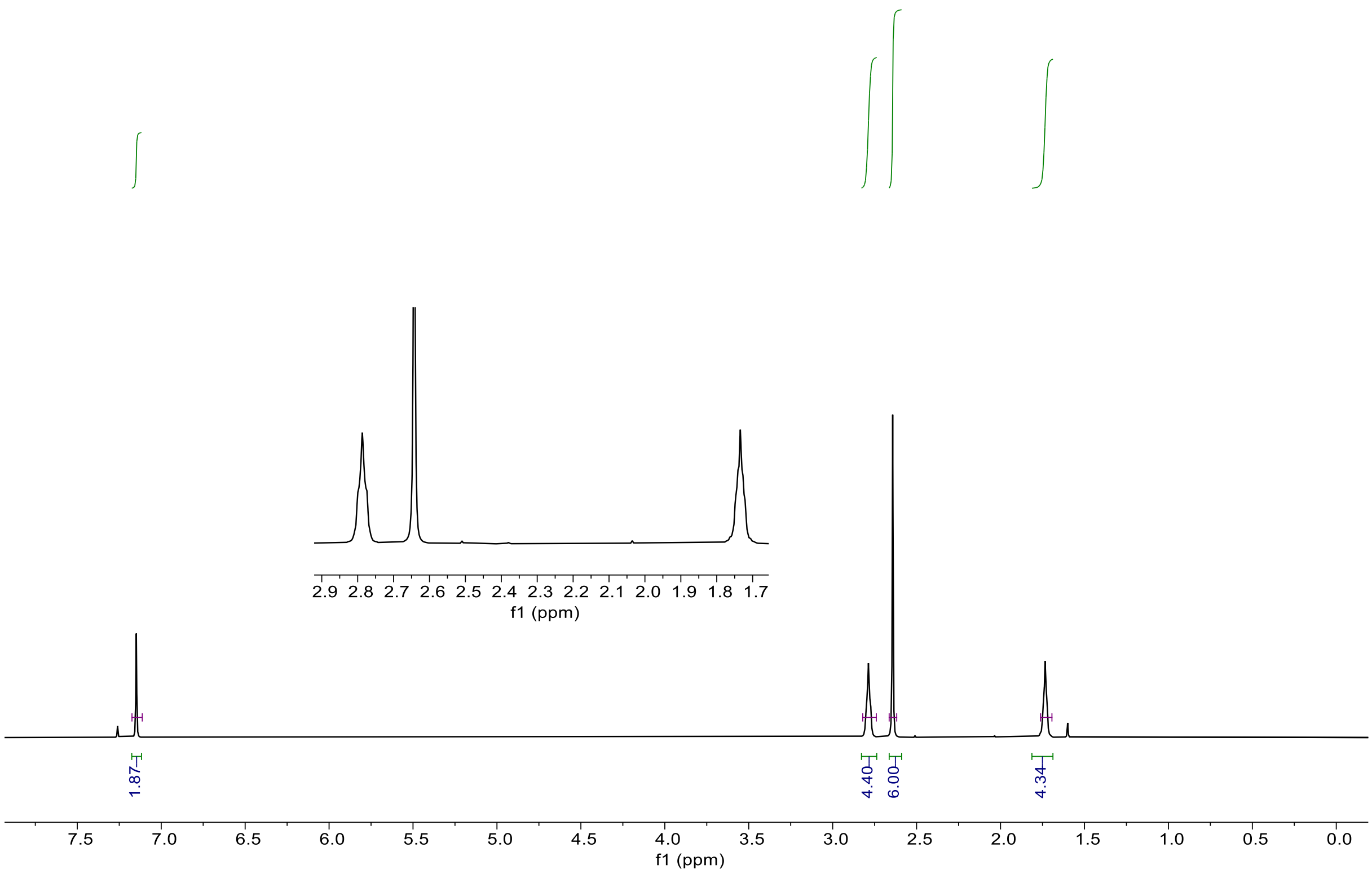

Compound **5** – ^{13}C, $CDCl_3$, 125.5 MHz (see Procedure)

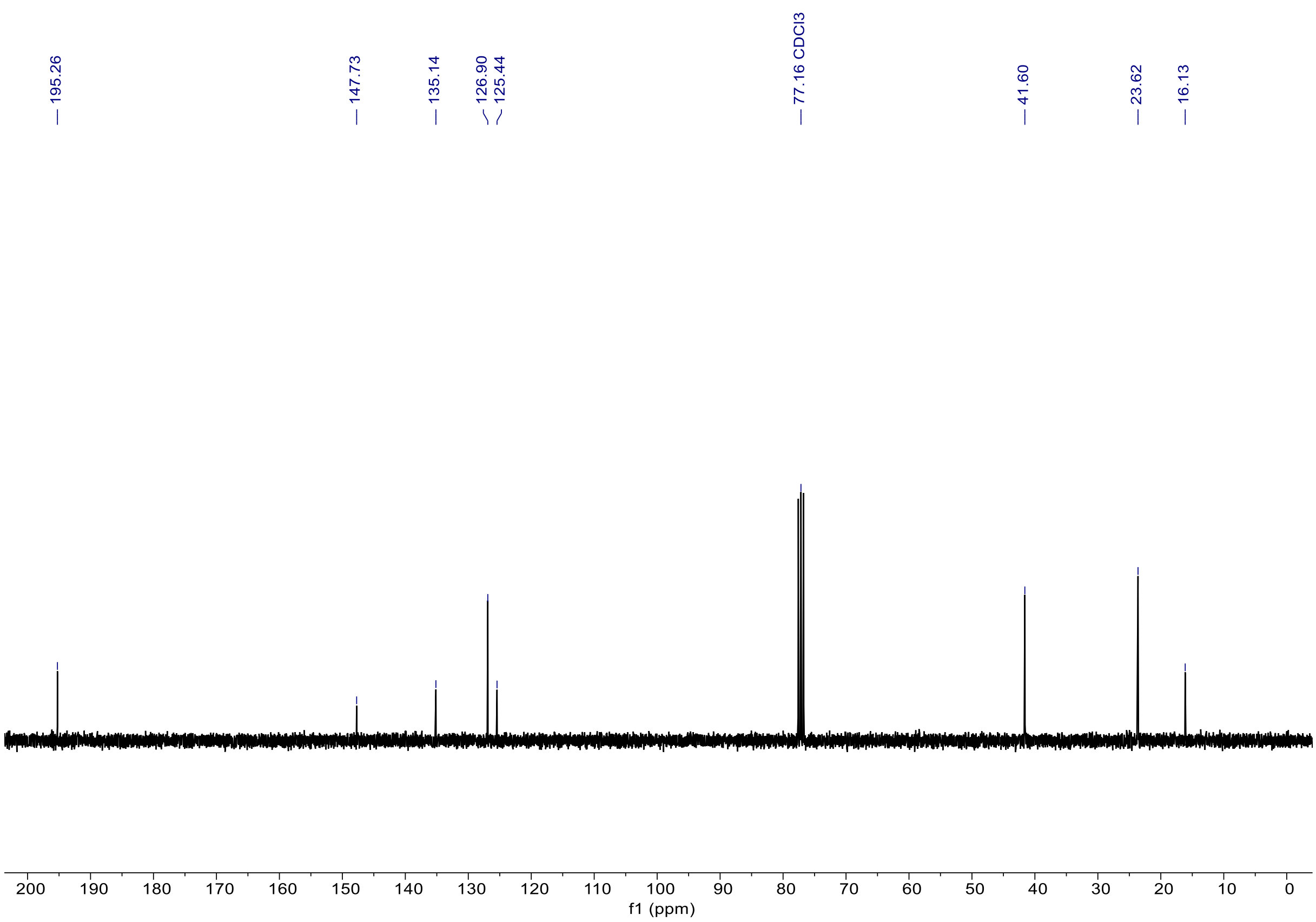

Compound **6** – ^{1}H, $CDCl_3$, 500 MHz (see Procedure)

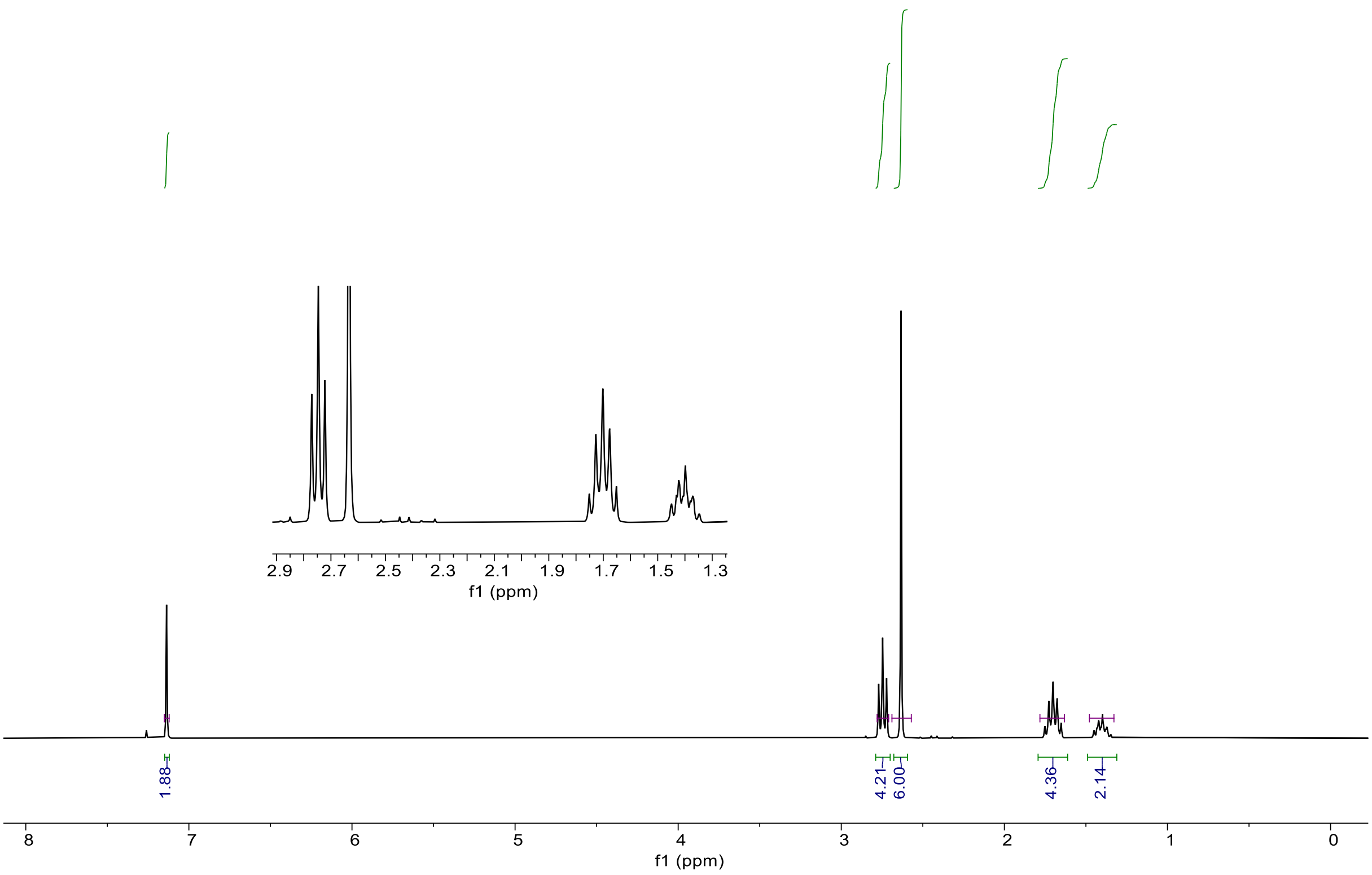

Compound **6** – ^{13}C, $CDCl_3$, 125.5 MHz (see Procedure)

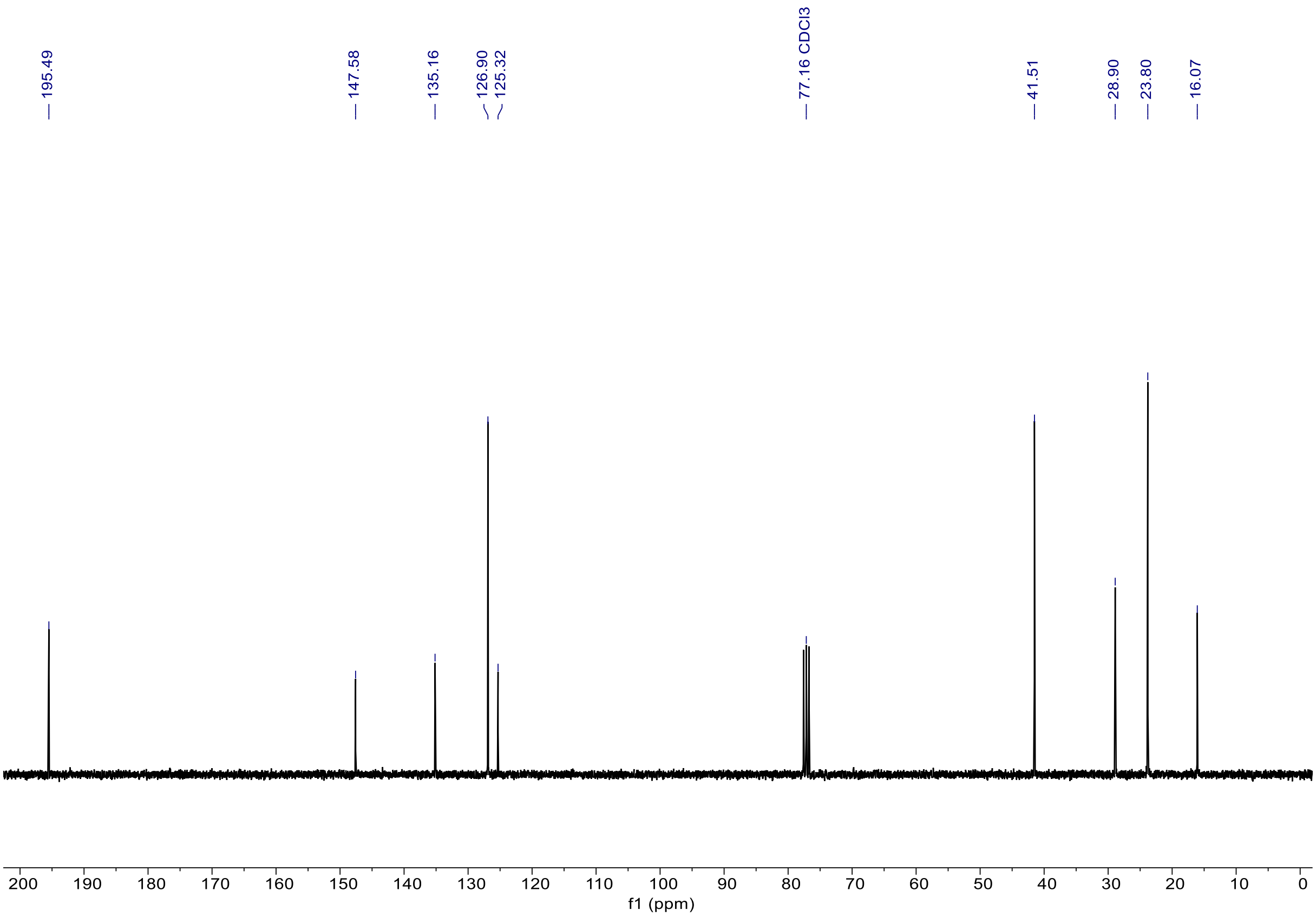

Compound **7** – ^{1}H, $CDCl_3$, 500 MHz (see Procedure)

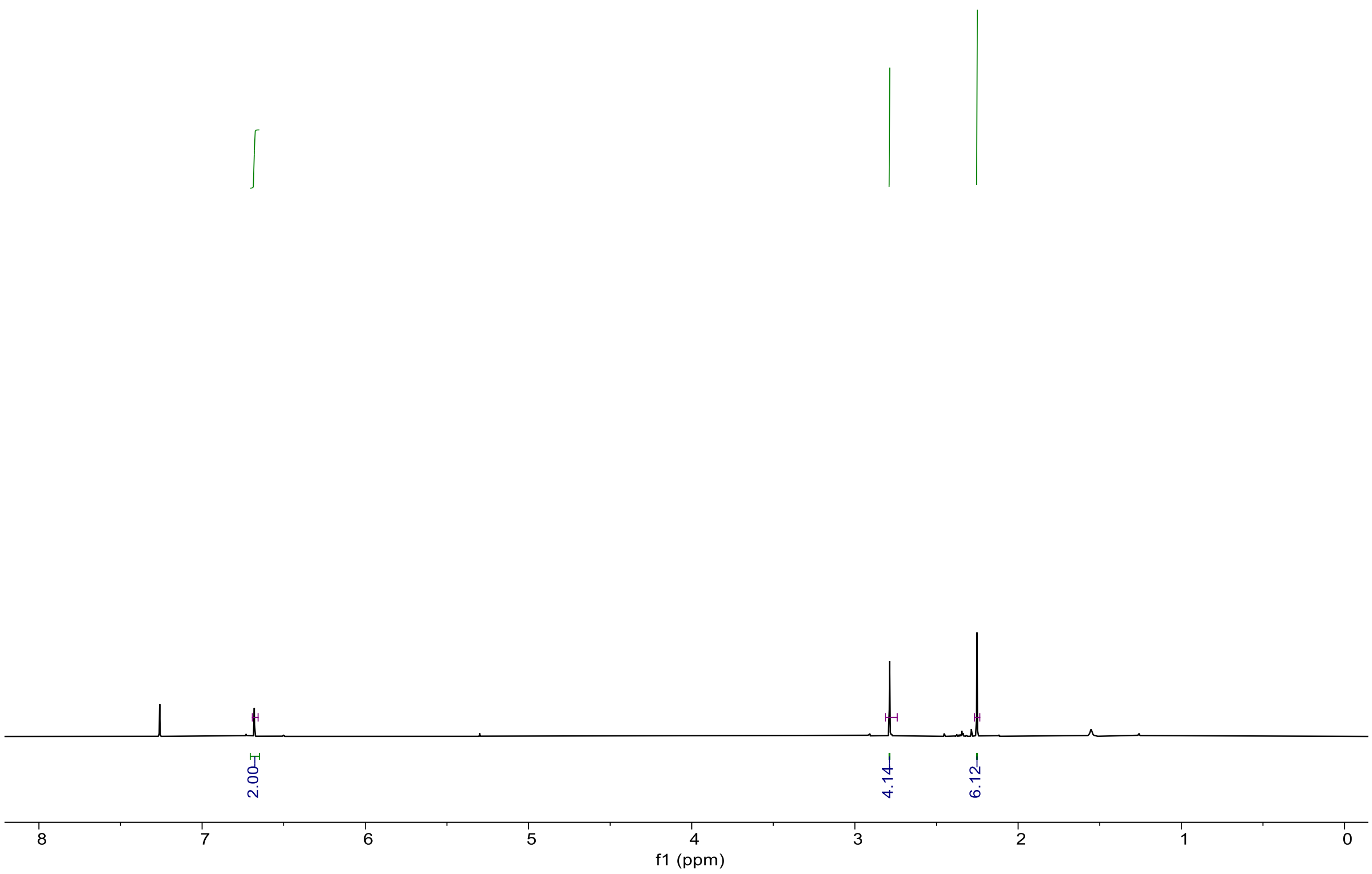

Compound **7** – ^{13}C, $CDCl_3$, 125.5 MHz (see Procedure)

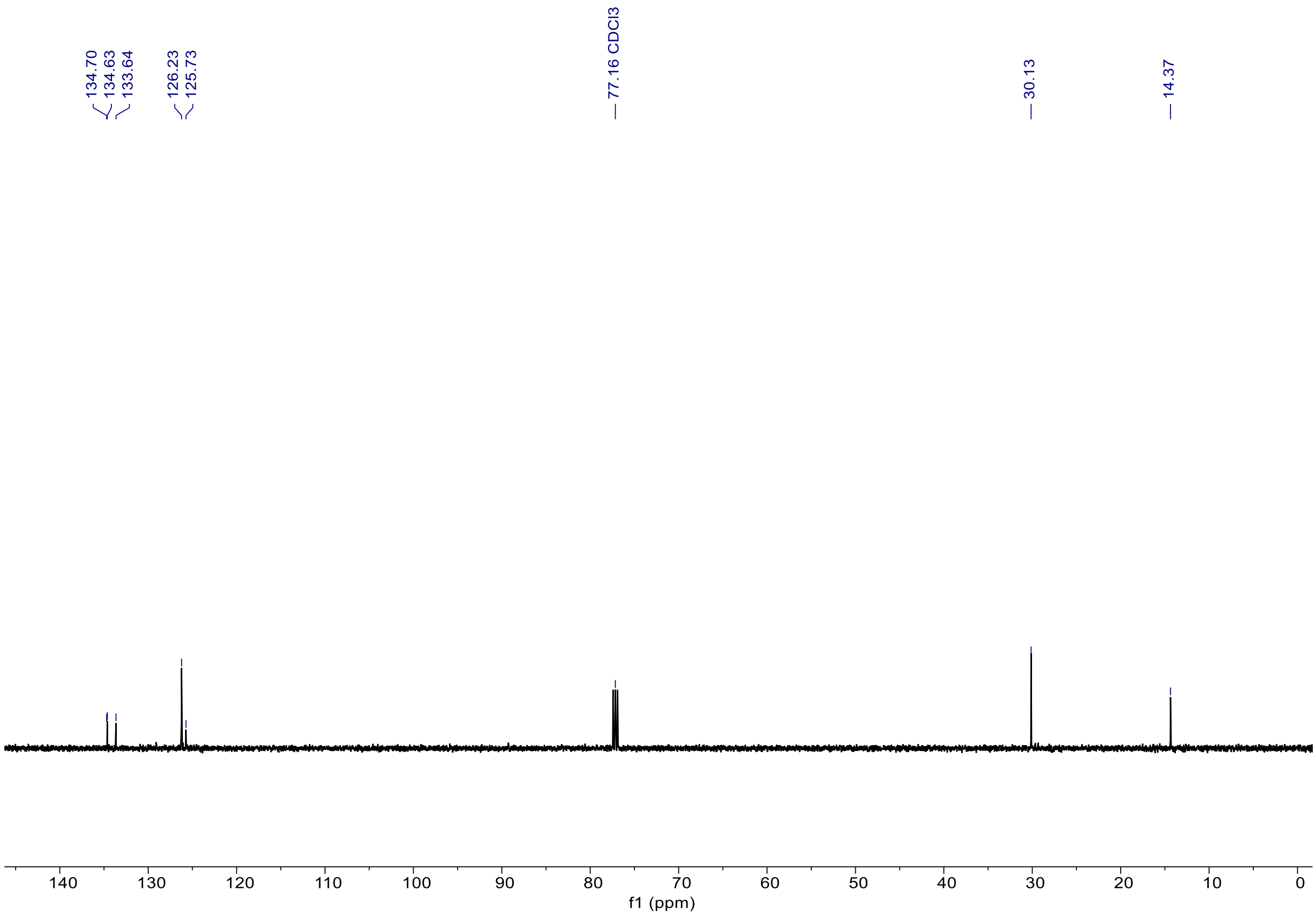

Compound **8** – ^{1}H, $CDCl_3$, 500 MHz (see Procedure)

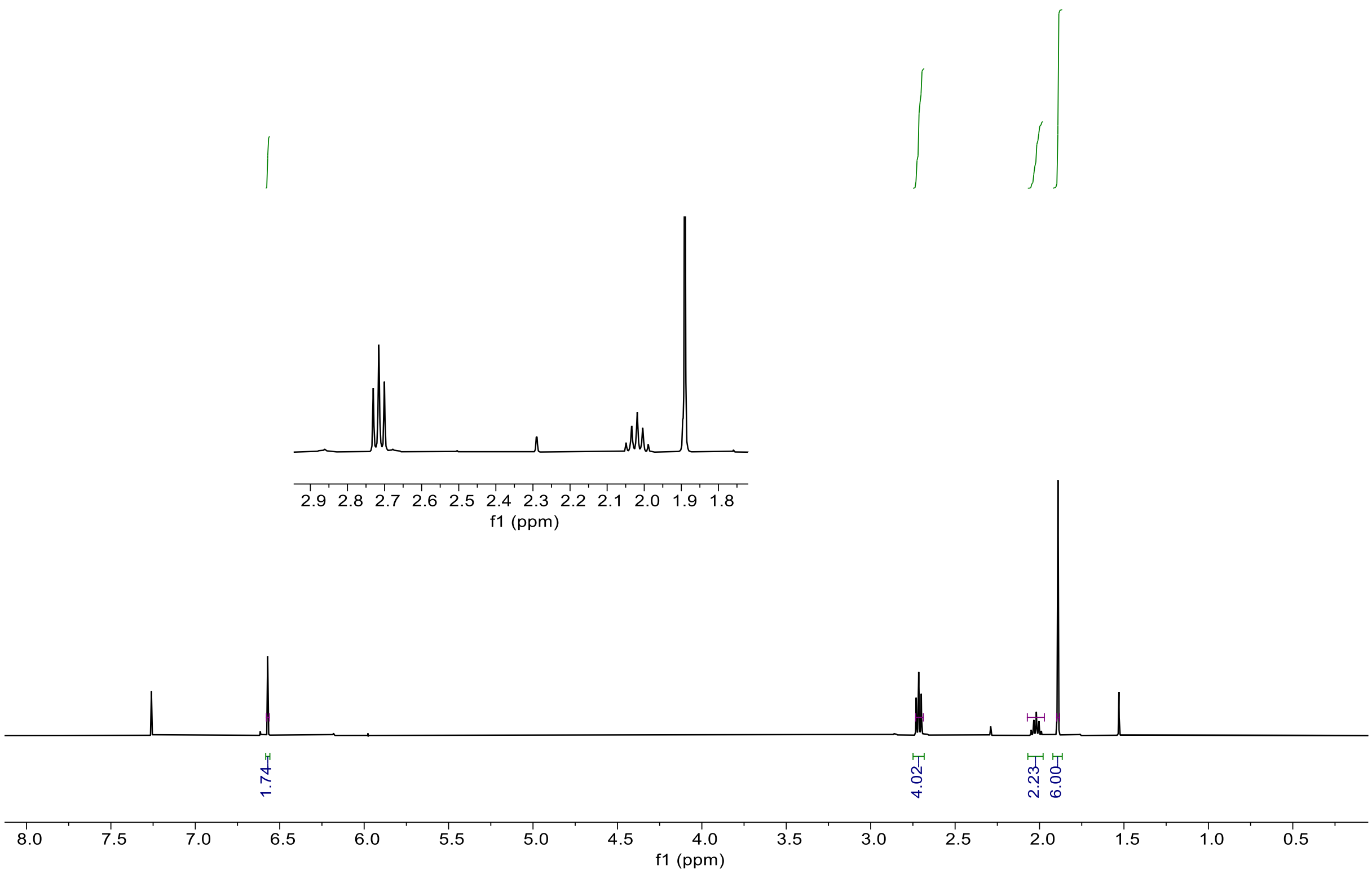

Compound **8** – ^{13}C, $CDCl_3$, 125.5 MHz (see Procedure)

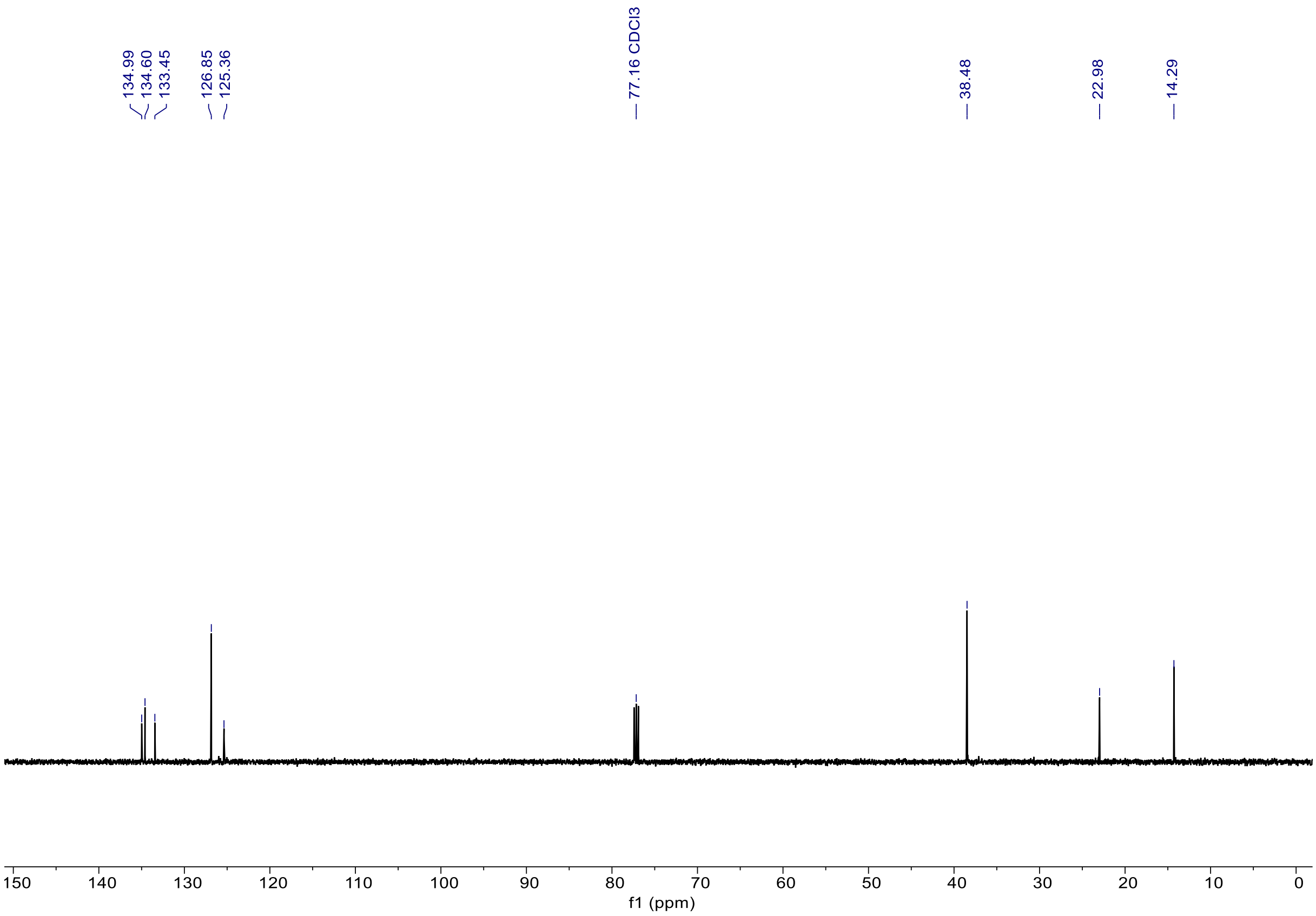

Compound **9** – ^{1}H, $CDCl_3$, 500 MHz (see Procedure)

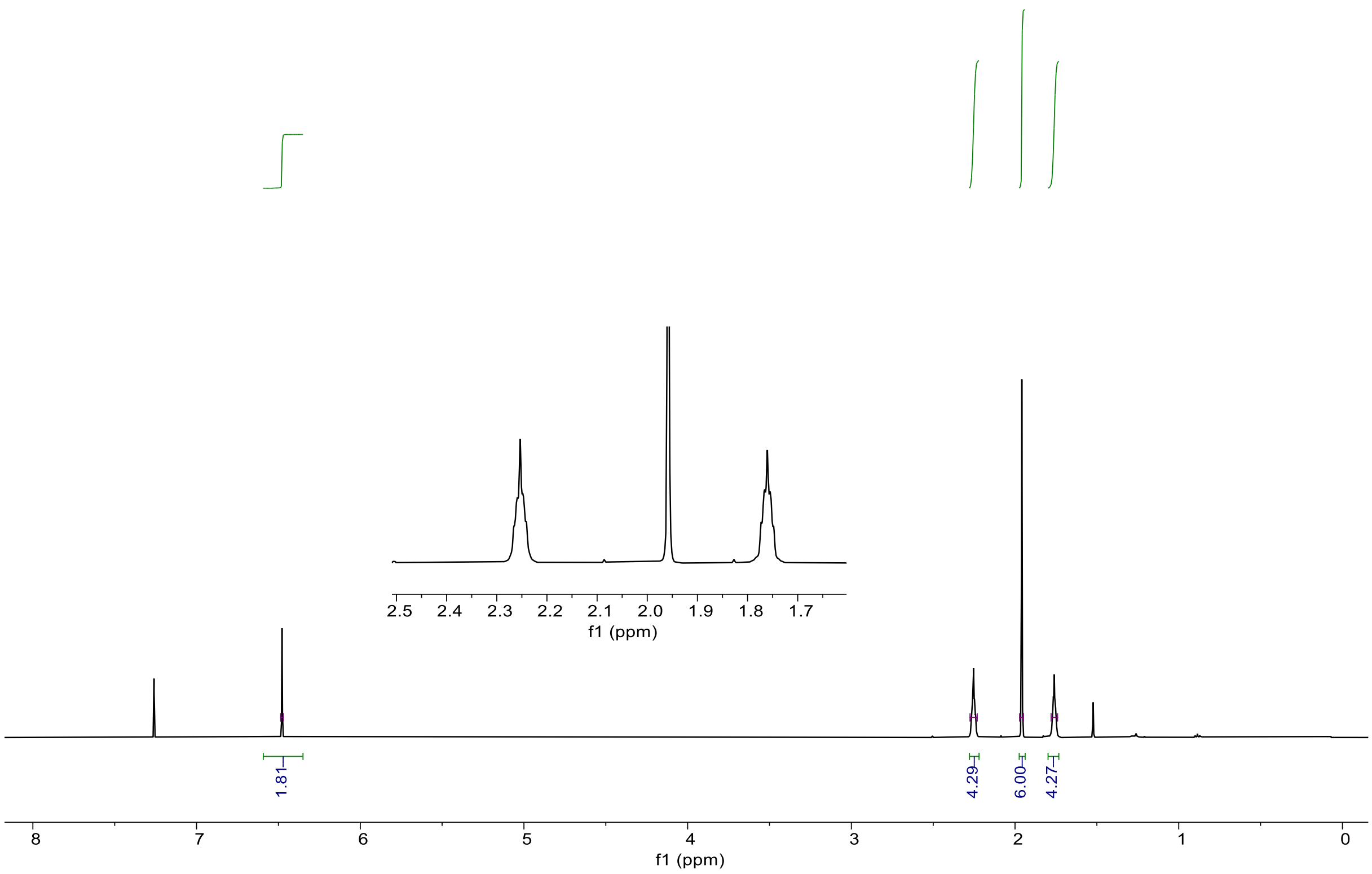

Compound **9** – ^{13}C, $CDCl_3$, 125.5 MHz (see Procedure)

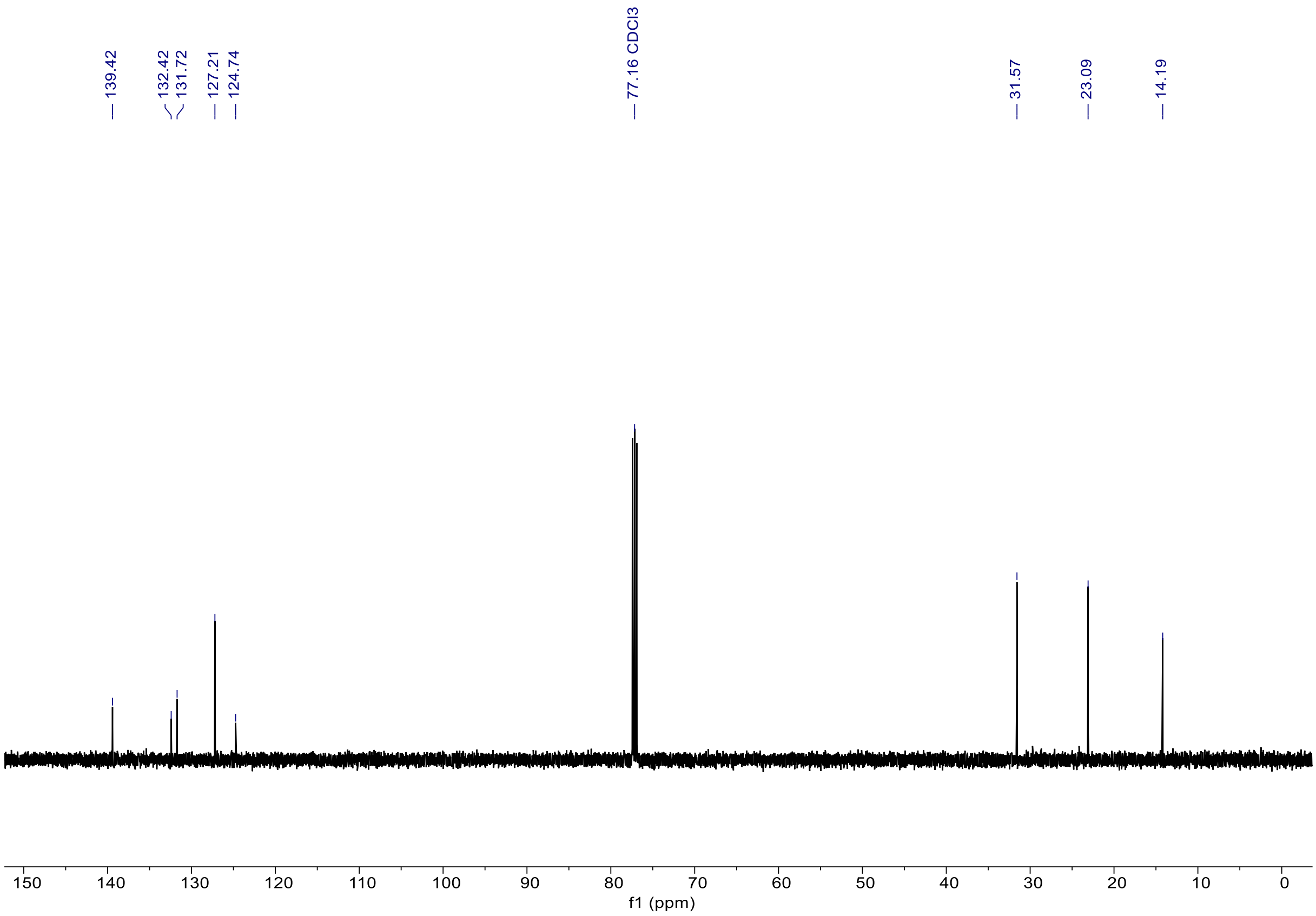

Compound **10** – ^{1}H, $CDCl_3$, 500 MHz (see Procedure)

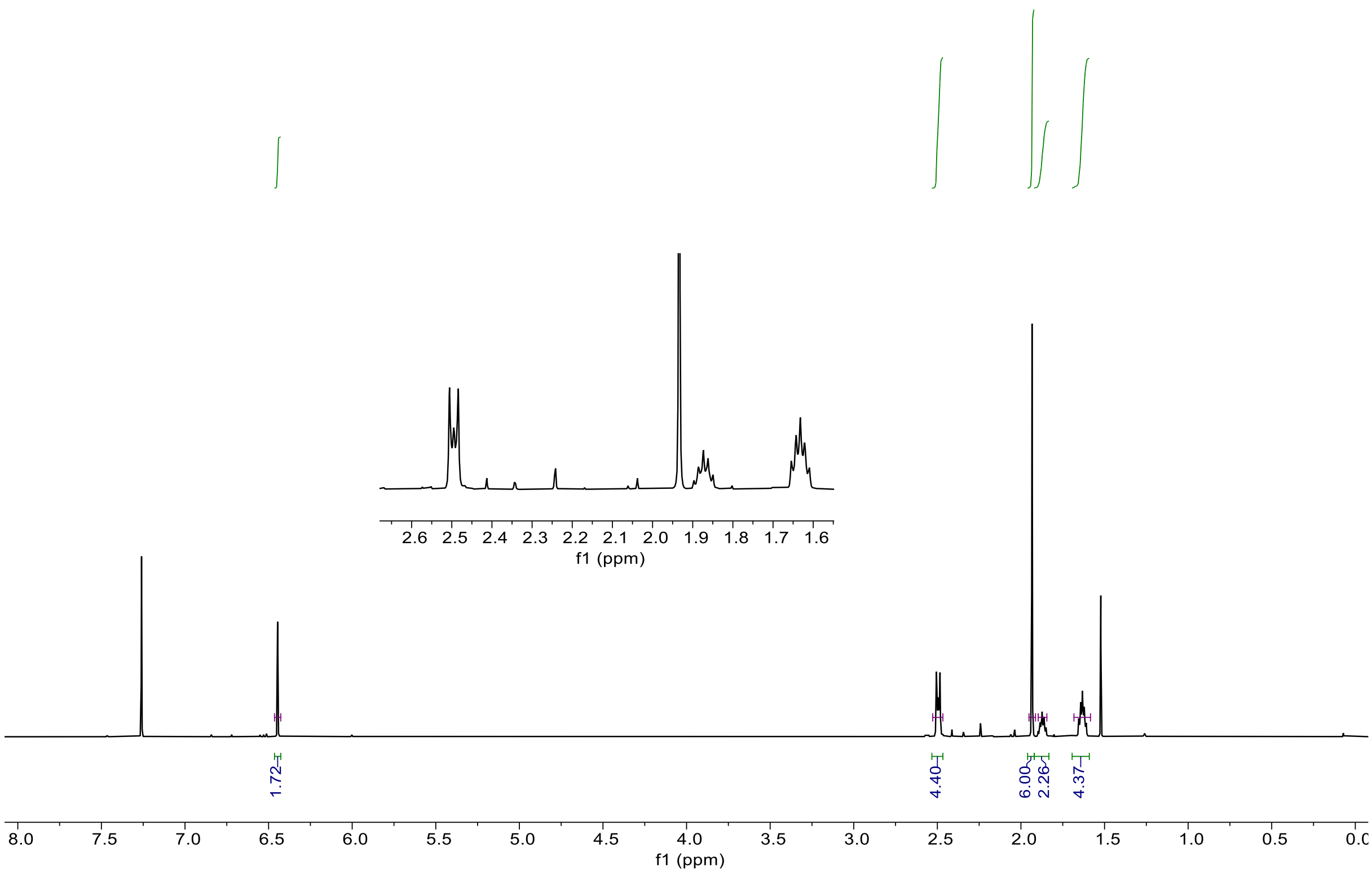

Compound **10** – ^{13}C, $CDCl_3$, 125.5 MHz (see Procedure)

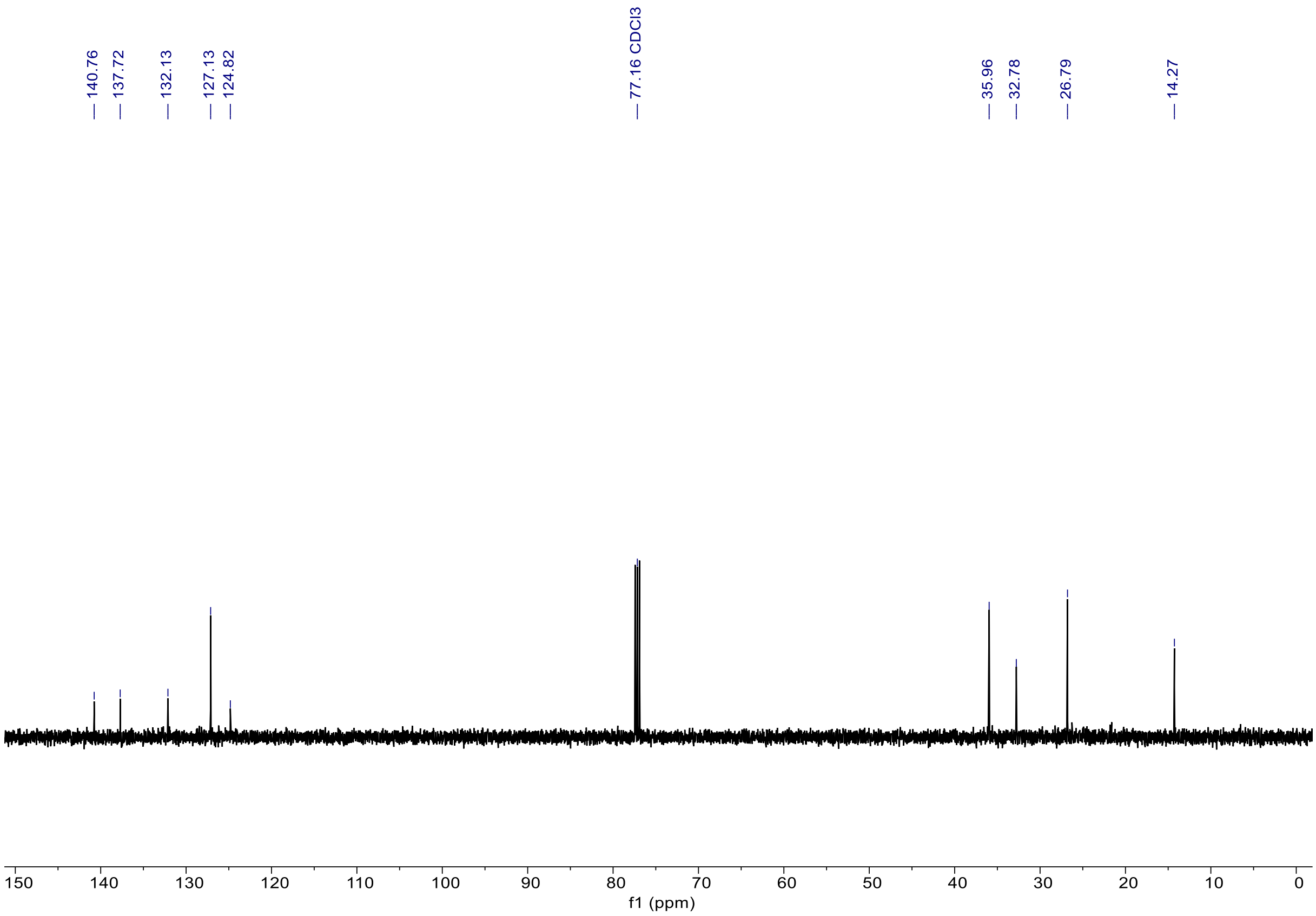

Compound **11** – ^{1}H, $CDCl_3$, 500 MHz (see Procedure)

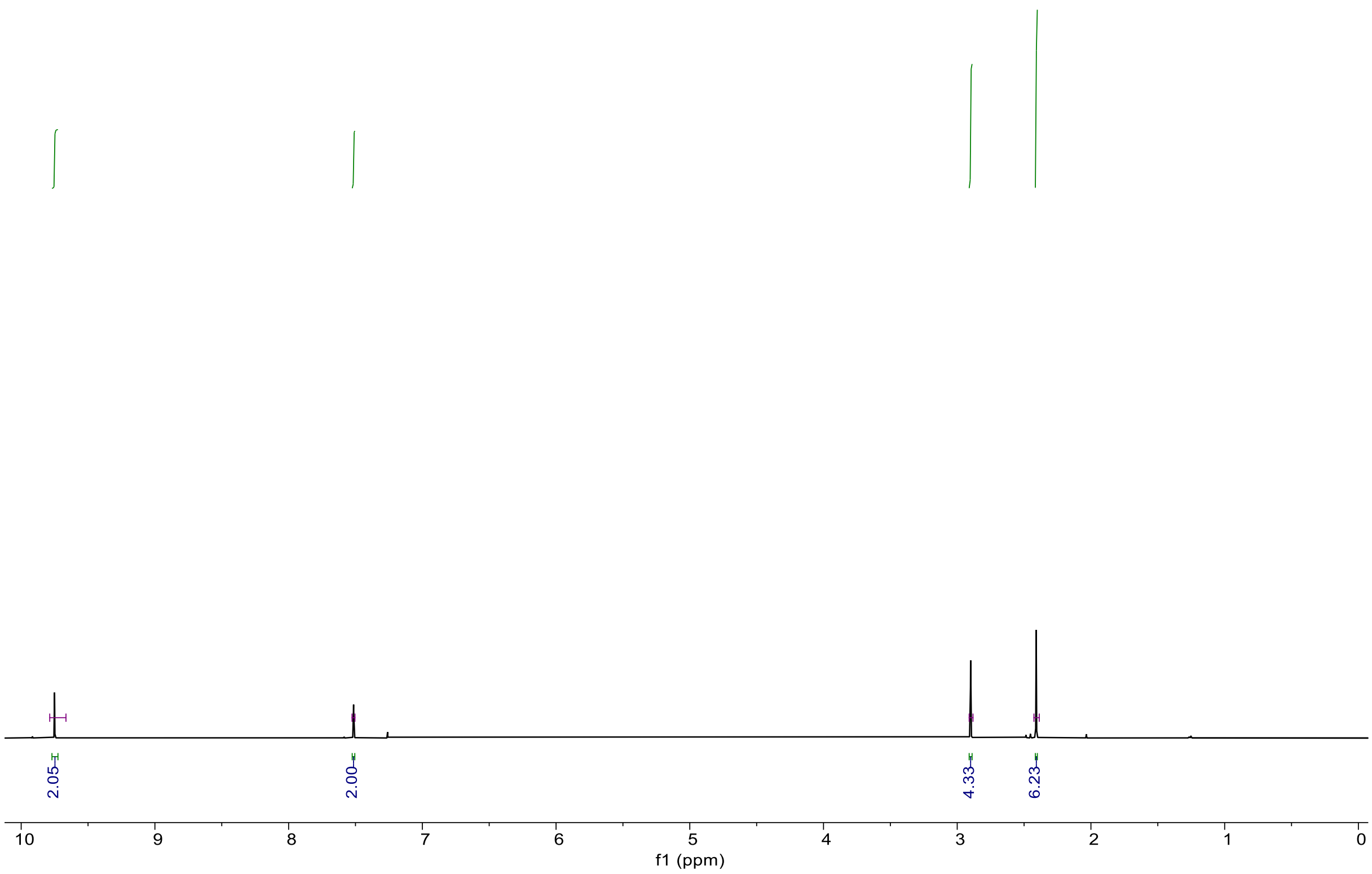

Compound **11** – ^{13}C, $CDCl_3$, 125.5 MHz (see Procedure)

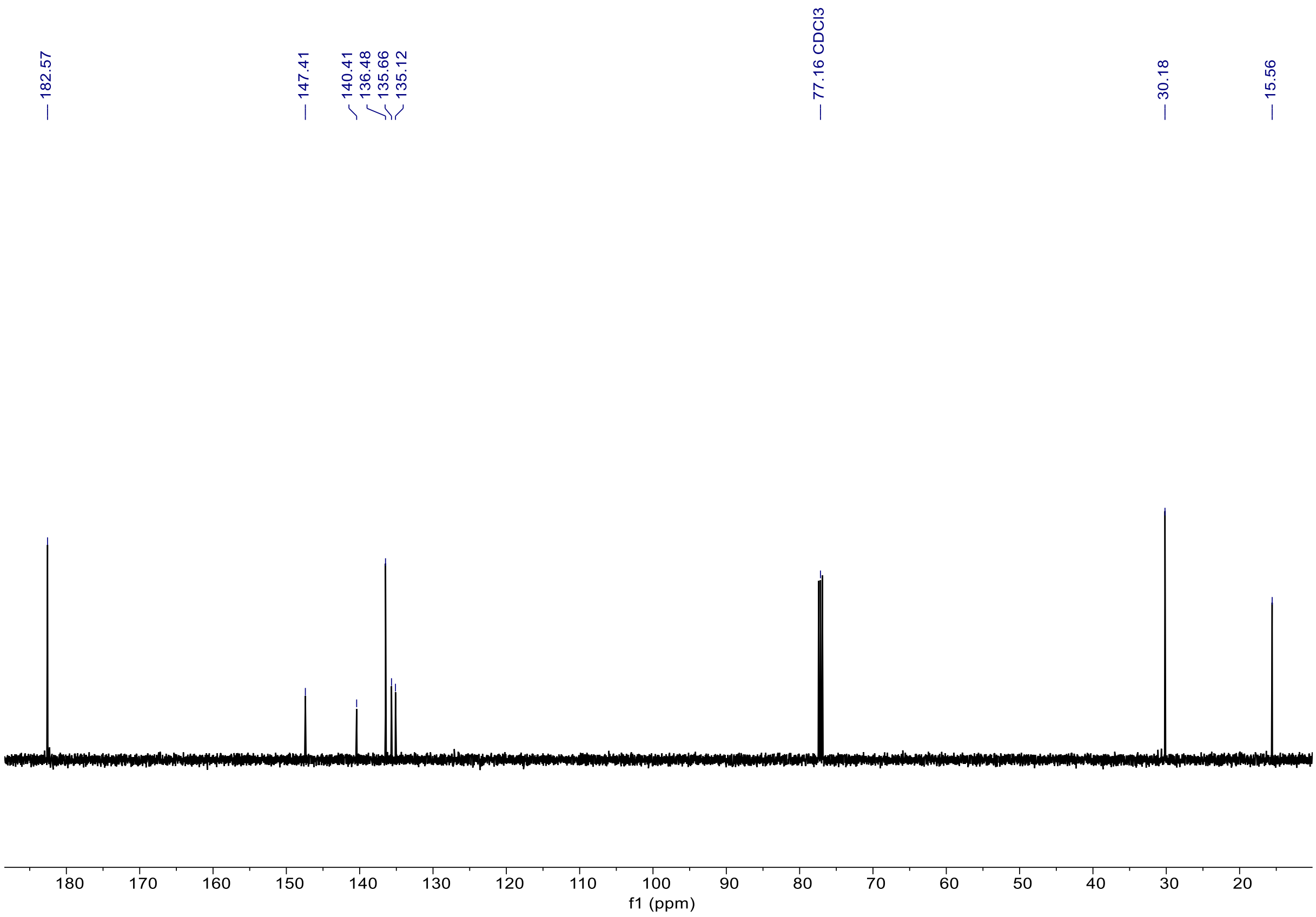

Compound **13** – ^{1}H, $CDCl_3$, 500 MHz (see Procedure)

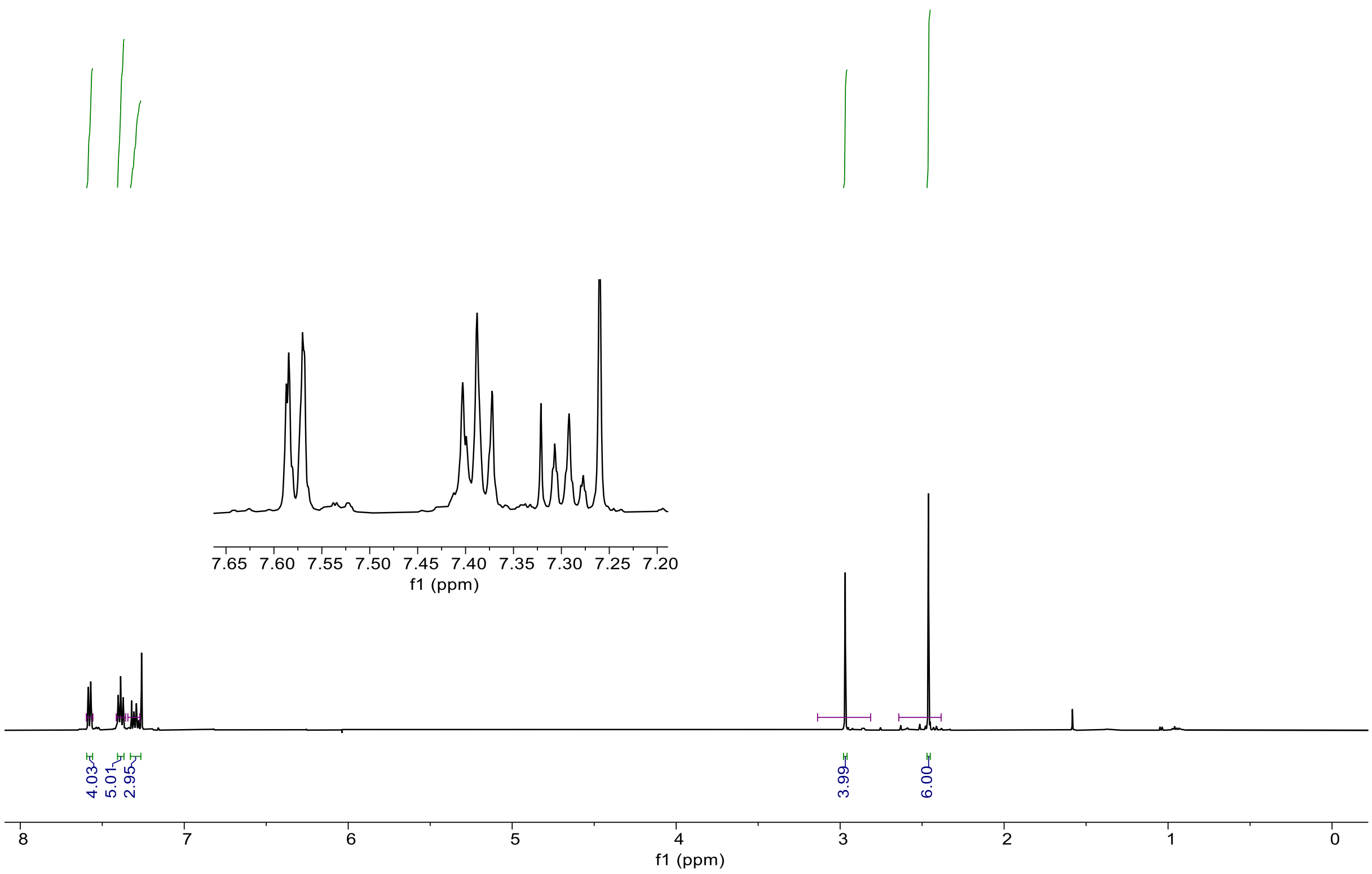

Compound **13** – ^{13}C, $CDCl_3$, 125.5 MHz (see Procedure)

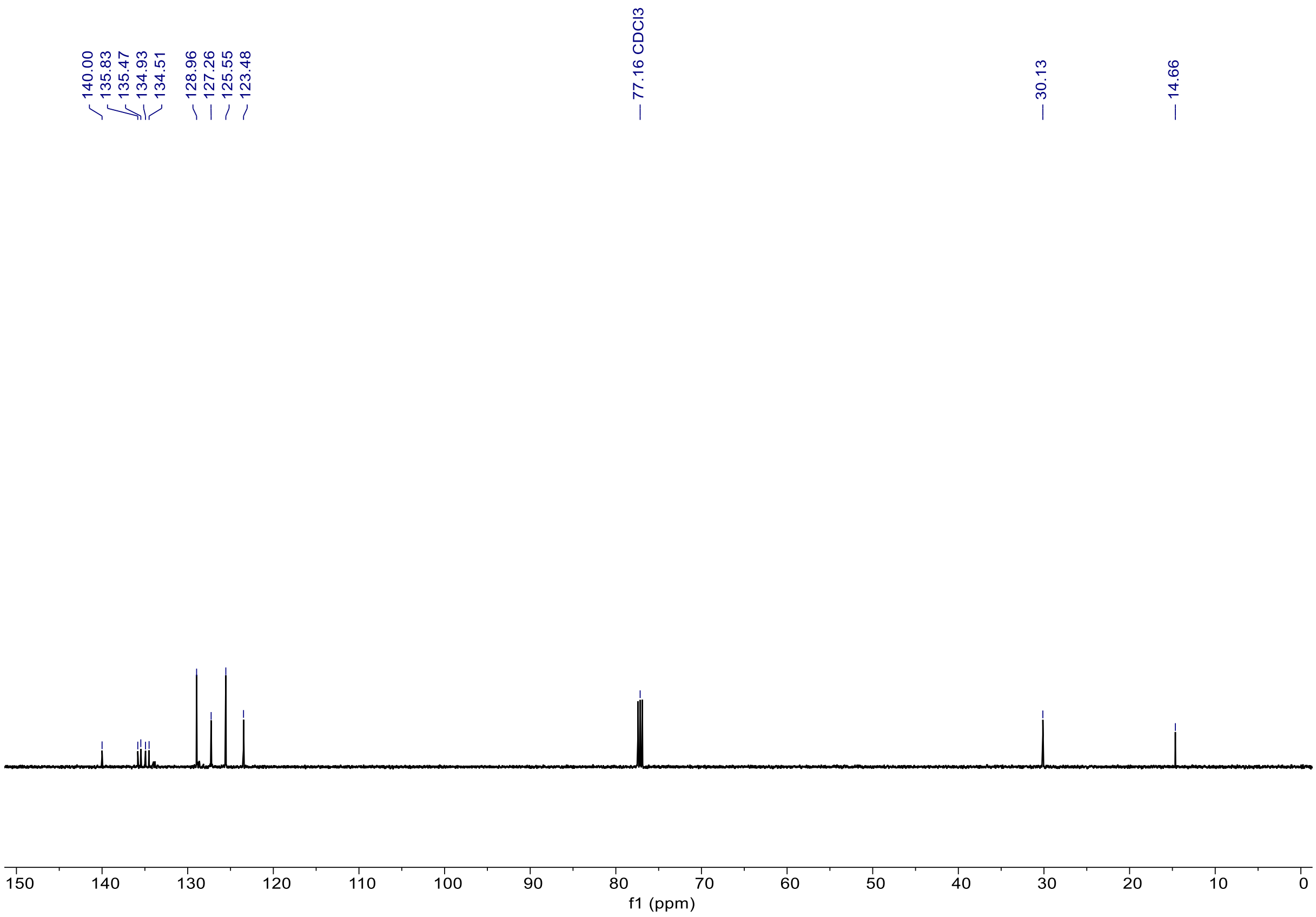

Compound **15** – ^{1}H, $CDCl_3$, 500 MHz (see Procedure)

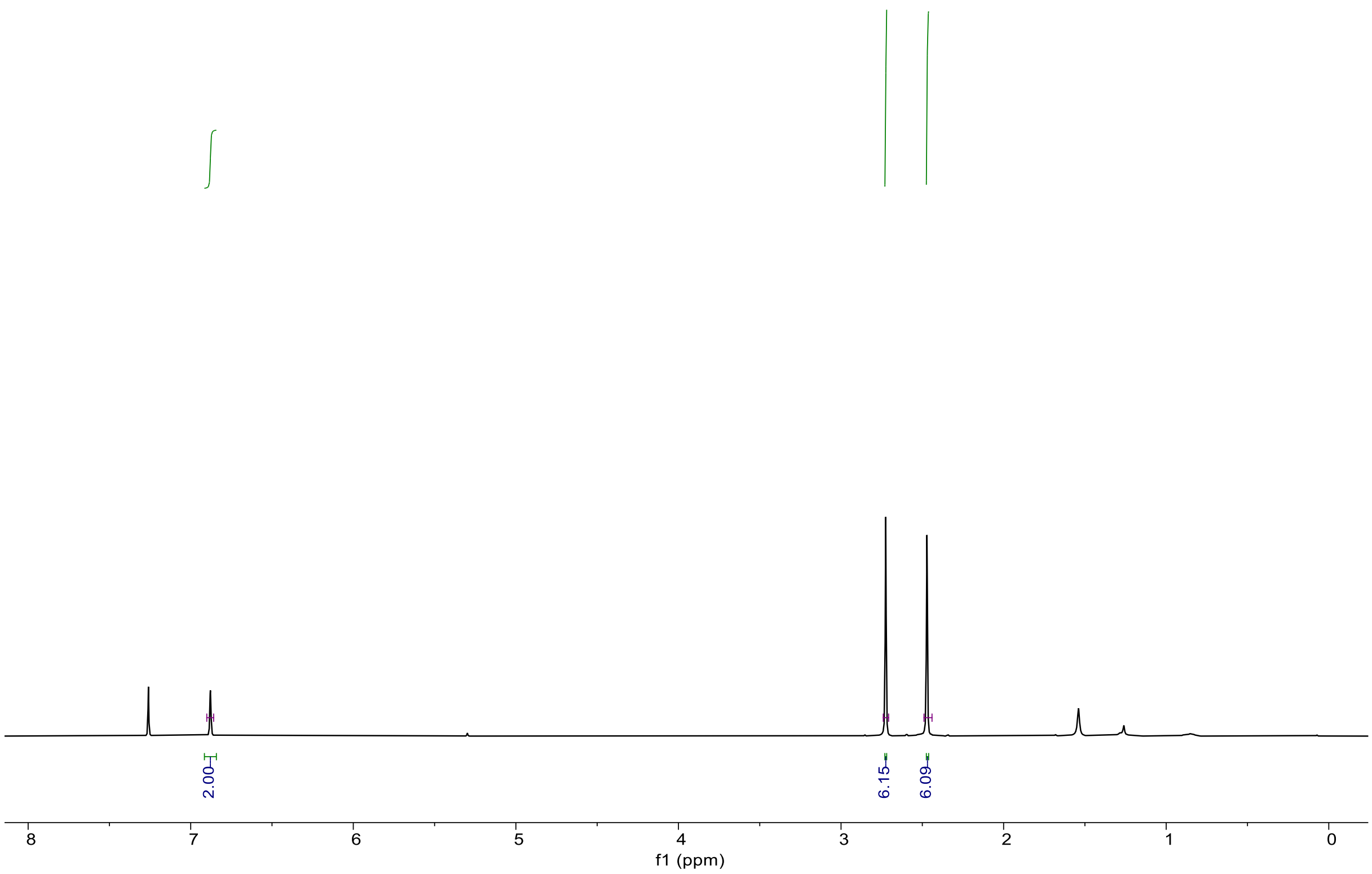

Compound **15** – ^{13}C, $CDCl_3$, 125.5 MHz (see Procedure)

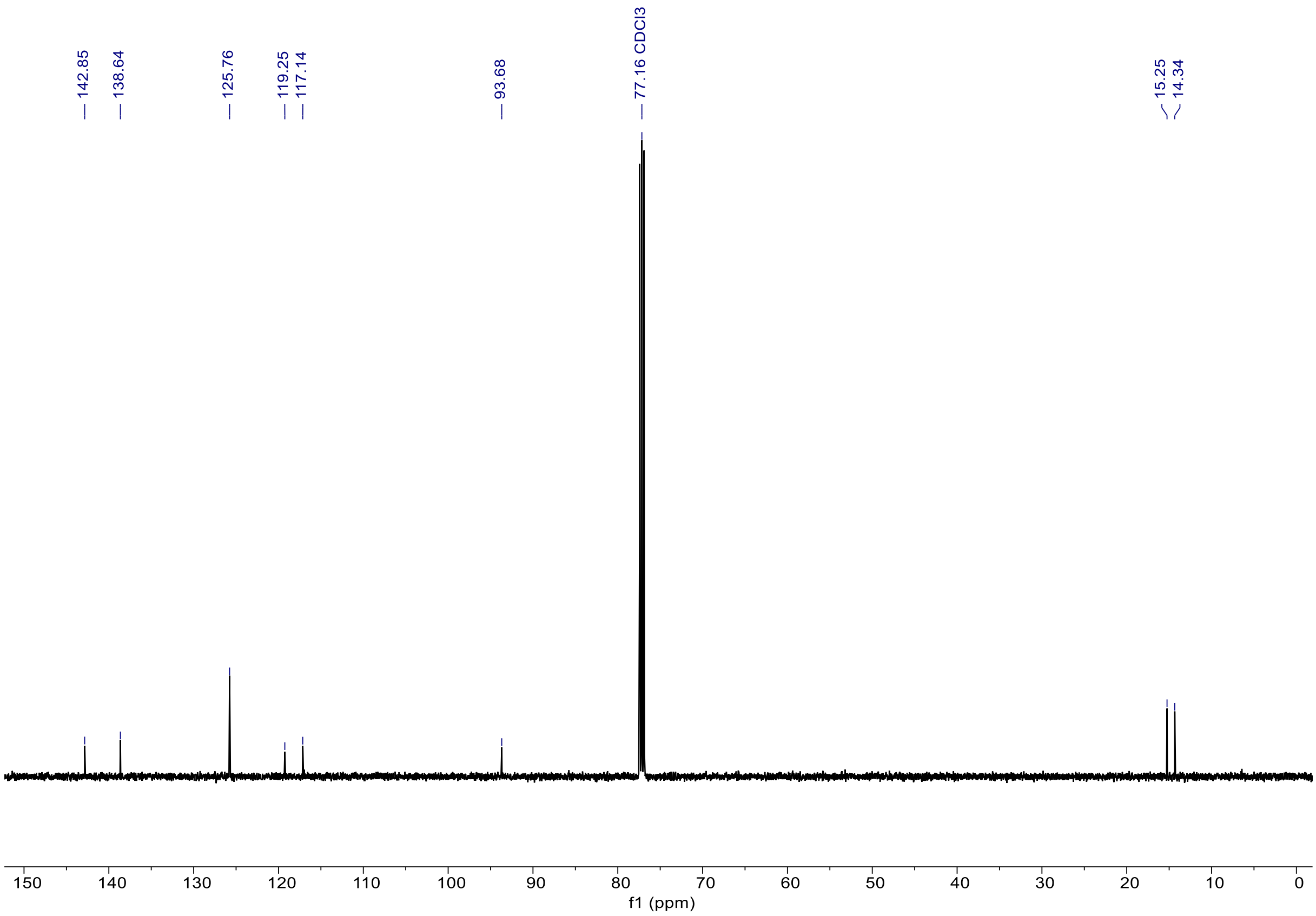

Compound **16** – ^{1}H, $CDCl_3$, 500 MHz (see Procedure)

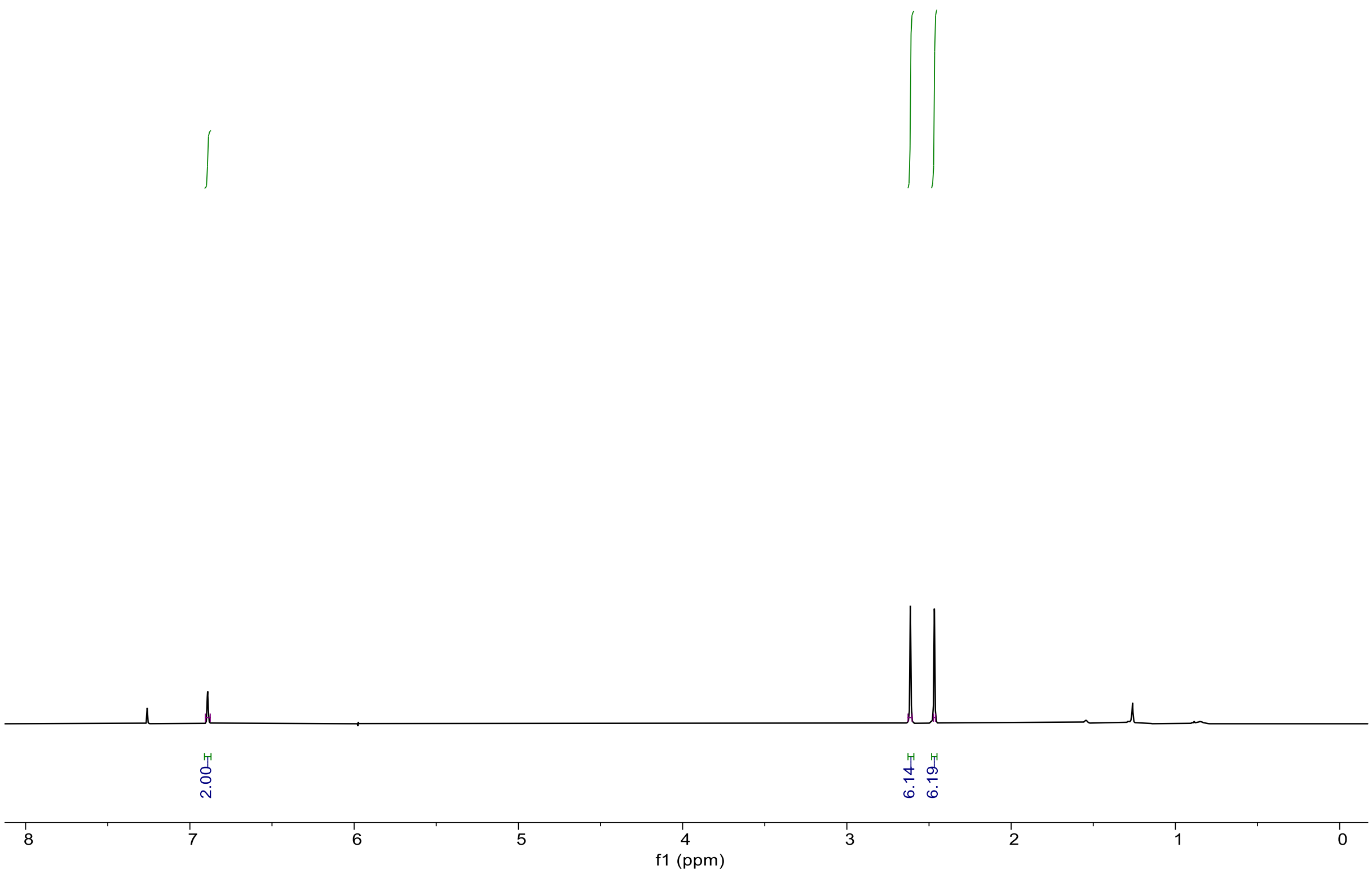

Compound **16** – ^{13}C, $CDCl_3$, 125.5 MHz (see Procedure)

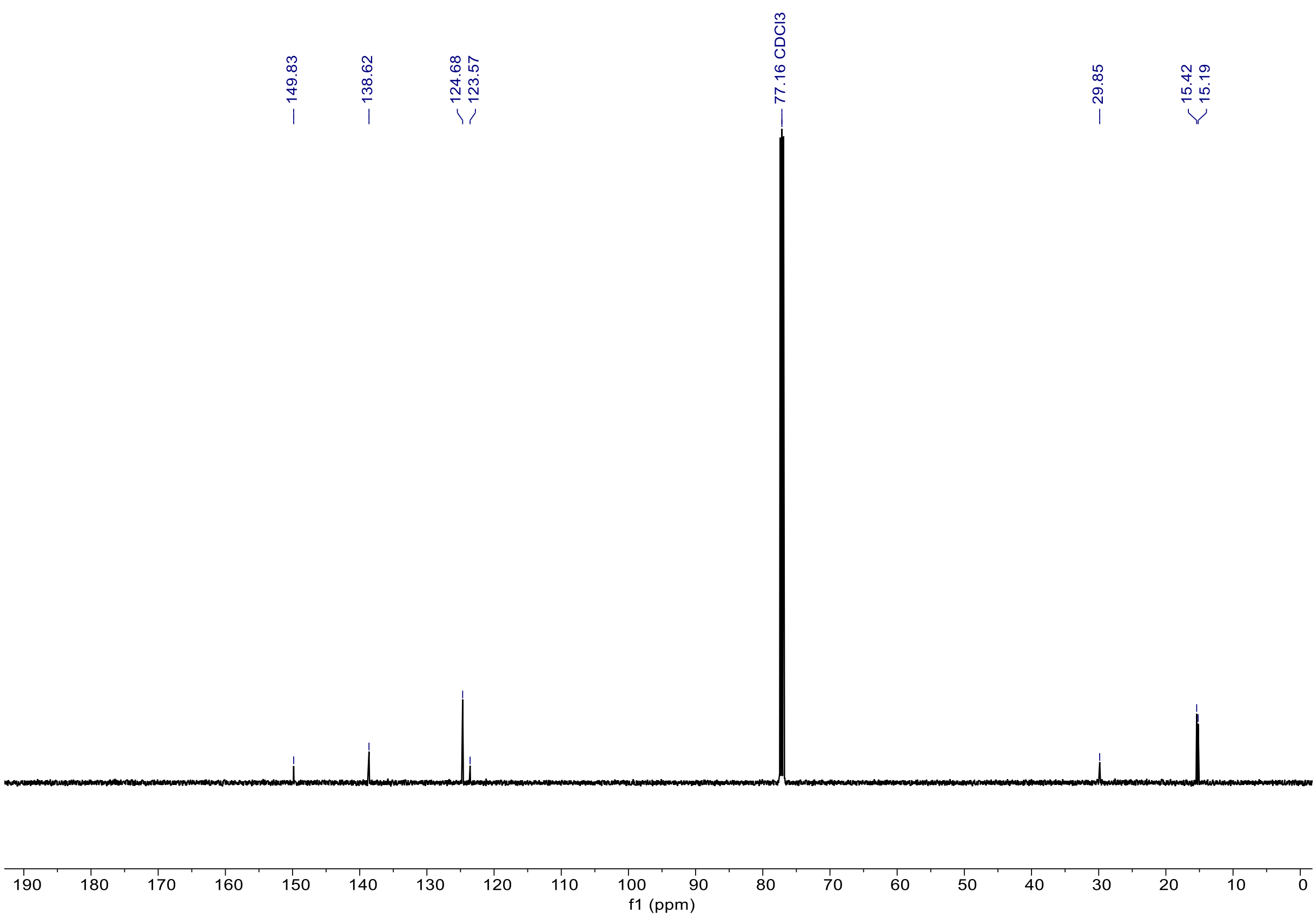

Compound **16** – ^{19}F, $CDCl_3$, 282.4 MHz (see Procedure)

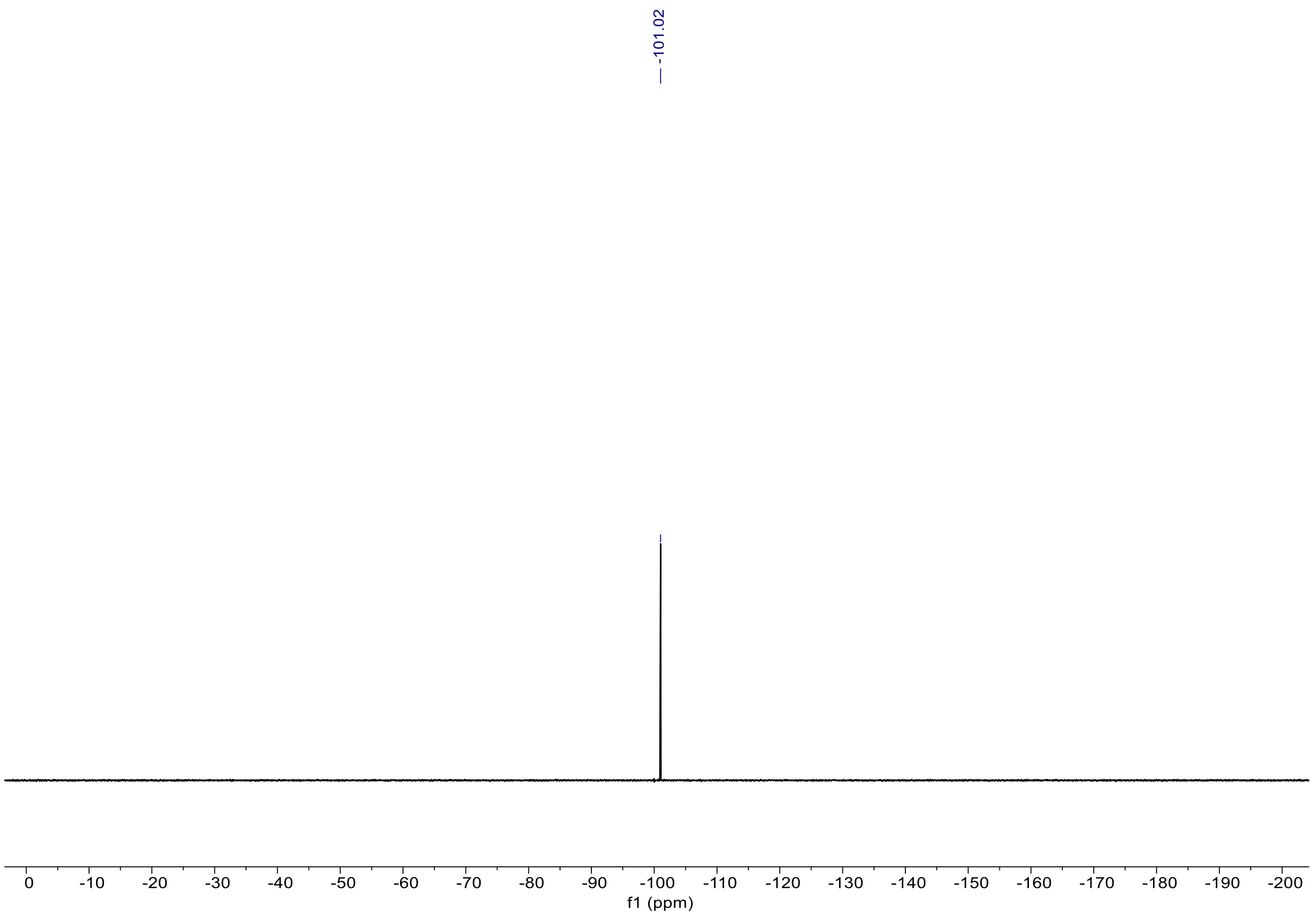

Compound **S13** – ^{1}H, $CDCl_3$, 500 MHz (see Procedure)

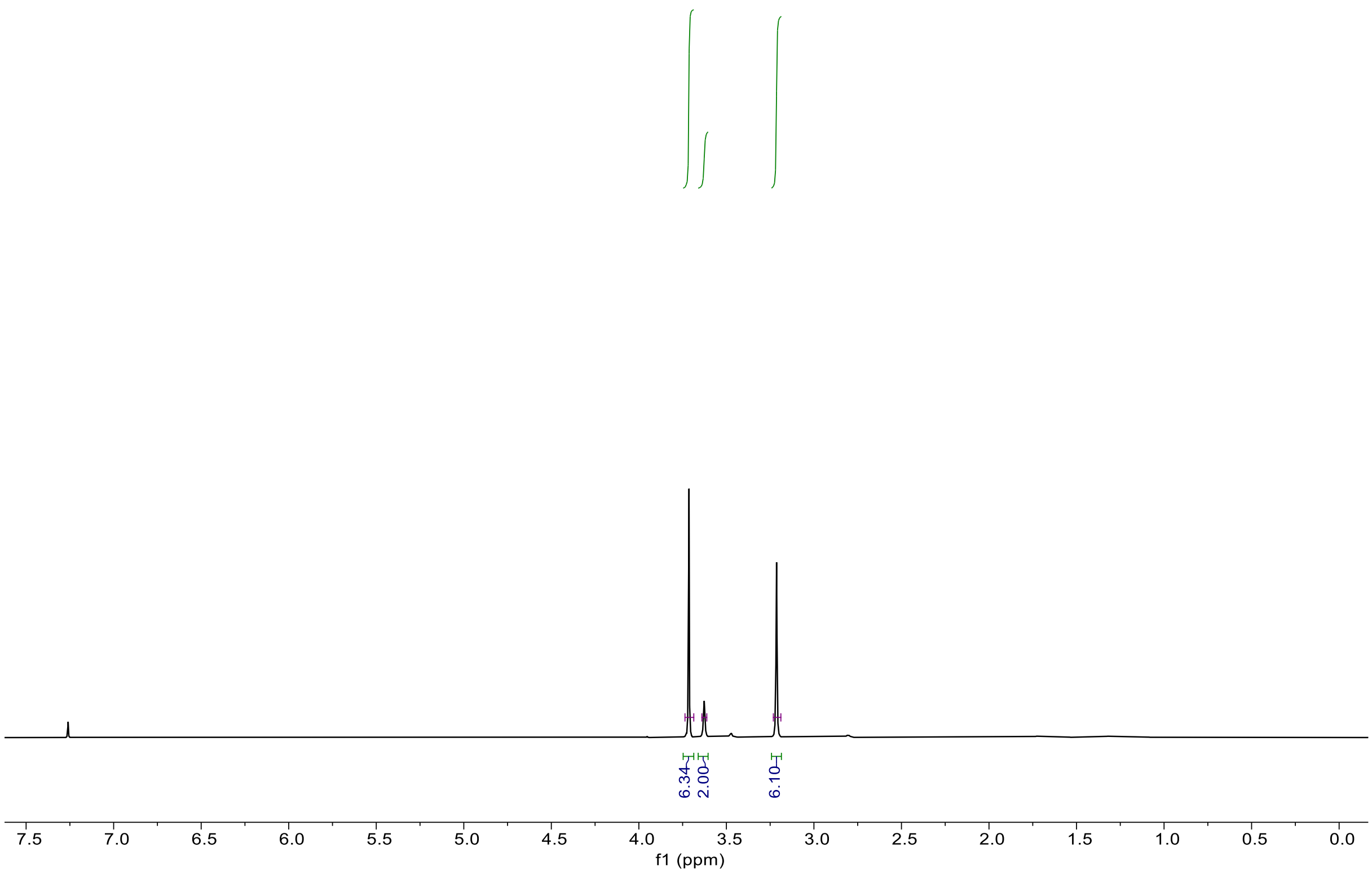

Compound **S13** – ^{13}C, $CDCl_3$, 125.5 MHz (see Procedure)

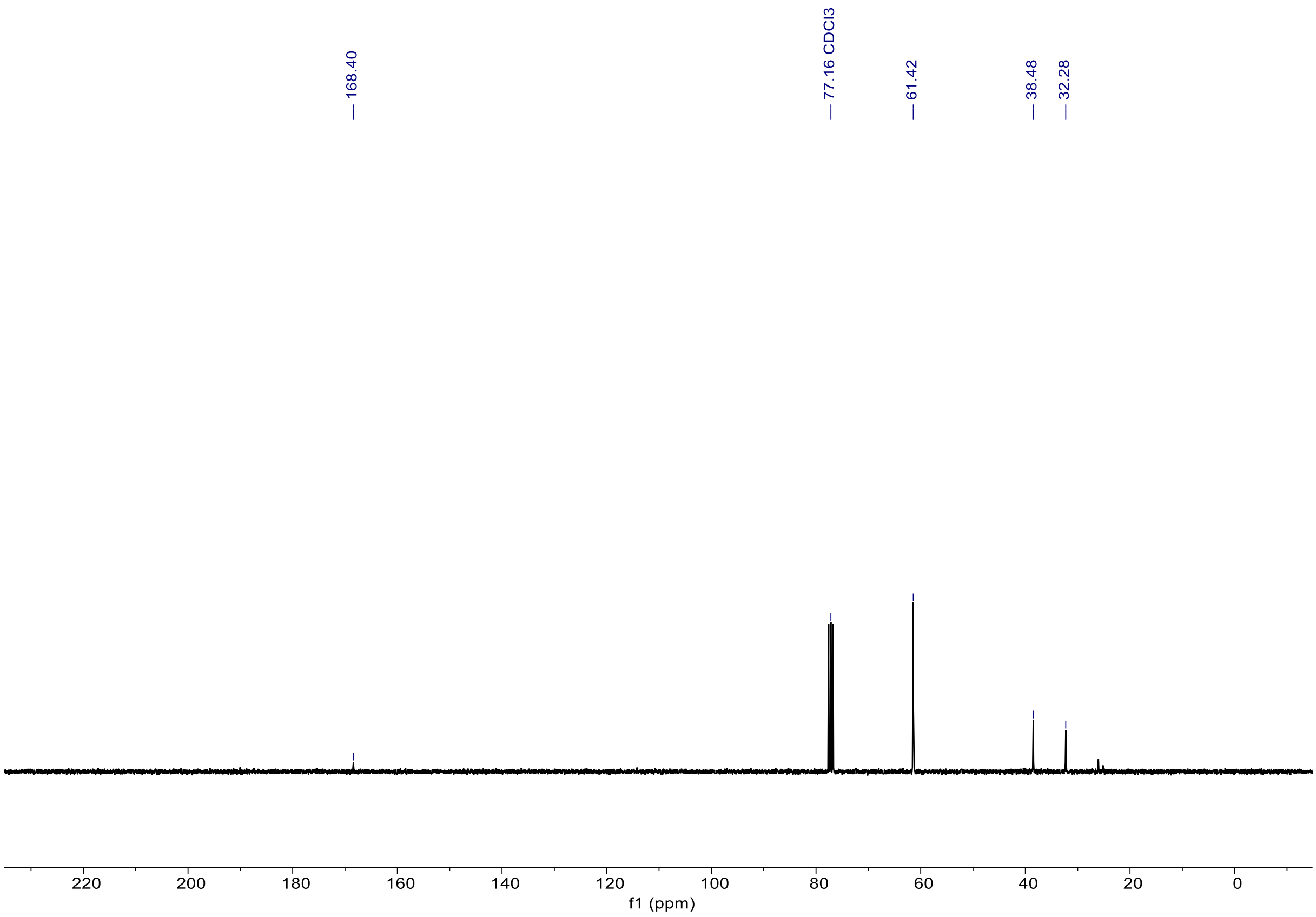

Supplement: Supplementary file 1 [file CHEM-32-e02730-s001.pdf]
